# Inhibition of fatty acid synthesis induces differentiation and reduces tumor burden in childhood neuroblastoma

**DOI:** 10.1016/j.isci.2021.102128

**Published:** 2021-02-01

**Authors:** María Victoria Ruiz-Pérez, Lourdes Sainero-Alcolado, Ganna Oliynyk, Isabell Matuschek, Nicola Balboni, S.J. Kumari A. Ubhayasekera, Marteinn Thor Snaebjornsson, Kamil Makowski, Kristina Aaltonen, Daniel Bexell, Dolors Serra, Roland Nilsson, Jonas Bergquist, Almut Schulze, Marie Arsenian-Henriksson

**Affiliations:** 1 Department of Microbiology, Tumor and Cell Biology (MTC), Biomedicum B7, Karolinska Institutet, 171 65 Stockholm, Sweden; 2 Analytical Chemistry, Department of Chemistry and Science for Life Laboratory, Uppsala University, 751 24 Uppsala, Sweden; 3 Tumor Metabolism and Microenvironment, German Cancer Research Center (DKFZ), 69120 Heidelberg, Germany; 4 Department of Inorganic and Organic Chemistry, Section of Organic Chemistry, Faculty of Chemistry, University of Barcelona, 08028 Barcelona, Spain; 5 Translational Cancer Research, Lund University, 22381 Lund, Sweden; 6 Department of Biochemistry and Physiology, School of Pharmacy, Institute of Biomedicine of the University of Barcelona (IBUB), University of Barcelona, 08028 Barcelona, Spain, and CIBER Physiopathology of Obesity and Nutrition (CIBEROBN), Institute of Health Carlos III, 28029 Madrid, Spain; 7 Cardiovascular Medicine Unit, Department of Medicine, Karolinska Institutet, 17176 Stockholm, Sweden; 8 Division of Cardiovascular Medicine, Karolinska University Hospital, 17176 Stockholm, Sweden; 9 Center for Molecular Medicine, Karolinska Institutet, 17176 Stockholm, Sweden

**Keywords:** biological sciences, molecular biology, cell biology, cancer

## Abstract

Many metabolic pathways, including lipid metabolism, are rewired in tumors to support energy and biomass production and to allow adaptation to stressful environments. Neuroblastoma is the second deadliest solid tumor in children. Genetic aberrations, as the amplification of the *MYCN*-oncogene, correlate strongly with disease progression. Yet, there are only a few molecular targets successfully exploited in the clinic. Here we show that inhibition of fatty acid synthesis led to increased neural differentiation and reduced tumor burden in neuroblastoma xenograft experiments independently of *MYCN*-status. This was accompanied by reduced levels of the MYCN or c-MYC oncoproteins and activation of ERK signaling. Importantly, the expression levels of genes involved in *de novo* fatty acid synthesis showed prognostic value for neuroblastoma patients. Our findings demonstrate that inhibition of *de novo* fatty acid synthesis is a promising pharmacological intervention strategy for the treatment of neuroblastoma independently of *MYCN*-status.

## Highlights

• Fatty acid synthesis inhibition reduces neuroblastoma growth *in vitro* and *in vivo*
• Decreased availability or reduced lipid synthesis downregulates MYC levels
• Impaired fatty acid synthesis induces neural differentiation through ERK activation
• High expression of fatty-acid-synthesis-related genes correlates with bad prognosis

## Graphical Abstract

**Figure:**
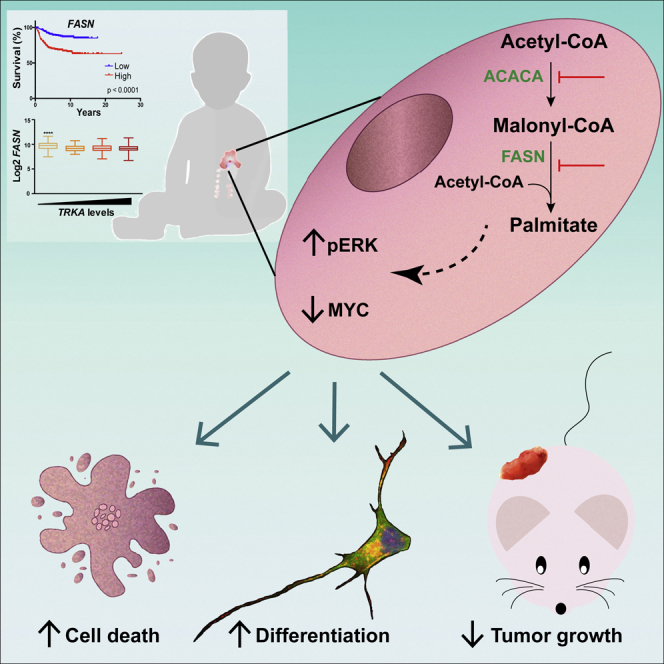

## Introduction

Neuroblastoma is the second most common solid tumor in children and accounts for up to 15% of cancer-related deaths during childhood (Maris, 2010). It develops in the adrenal gland and the peripheral nervous system from neural crest-derived precursors of the sympathetic nervous system. Neuroblastoma presentation is highly heterogeneous, both in terms of biological features and clinical response. According to the International Neuroblastoma Staging System (INSS), patients are classified into five different stages (1–4 and 4S). Although stage 4 patients have the lowest survival probability, 4S tumors frequently undergo spontaneous regression and the children thus have a good prognosis (Ikeda et al., 2002). In addition, neuroblastoma patients are classified into three risk groups: high, medium, and low. Patients with low-risk neuroblastomas undergo little or no clinical intervention, showing a survival probability of 90%–95%. In contrast, high-risk patients have an event-free survival probability of less than 50% (Whittle et al., 2017), suffer tumor recurrence and metastasis, and develop treatment resistance leading to a fatal outcome.

Amplification of *MYCN* is used as a diagnostic parameter to classify neuroblastoma patients into stages and risk groups. *MYCN*-amplified neuroblastomas are always considered as high risk. In fact, *MYCN* amplification, occurring in around 25% of all primary neuroblastomas and in 30%–40% of all high-risk cases (Pugh et al., 2013; Huang and Weiss, 2013), has since long been known to correlate to poor clinical outcome (Brodeur et al., 1984). Both *MYCN* and *c-MYC* belong to the *MYC* oncogene family. It has been shown that MYCN promotes pluripotency and blocks differentiation pathways (Ruiz-Perez et al., 2017). Importantly, induction of differentiation in neuroblastoma cells requires early *MYCN* expression (Guglielmi et al., 2014), followed by downregulation (Huang and Weiss, 2013).

Although c-MYC is involved in the control of multiple metabolic processes, including lipid metabolism (Eberlin et al., 2014; Edmunds et al., 2014; Morrish et al., 2010; Gouw et al., 2019; Carroll et al., 2018; Dejure and Eilers, 2017), the role of MYCN in metabolism is less clear. It has been shown to drive increased glutathione biosynthesis (Carter et al., 2016) and the overexpression of the glutamine transporter ASCT2, whose levels correlate to bad prognosis in neuroblastoma patients (Ren et al., 2015). *MYCN* overexpression, in cooperation with MondoA, led to increased levels of proteins involved in lipid metabolism, whereas MYCN and MondoA inactivation was connected to reduced *de novo* fatty acid synthesis (Carroll et al., 2015). MYCN drives changes in mitochondrial fusion, morphology, and function related to apoptosis resistance and to metabolic processes (Casinelli et al., 2016; Zirath et al., 2013; Oliynyk et al., 2019). Our group recently showed that MYCN promotes glycolysis, oxidative metabolism, and *de novo* glutamine synthesis. Importantly, neuroblastoma highly relies on fatty acid oxidation for energy production, and inhibition of β-oxidation leads to reduced growth of *MYCN*-amplified, but not of non-*MYCN*-amplified, cells and tumors (Oliynyk et al., 2019).

**Figure 1:**
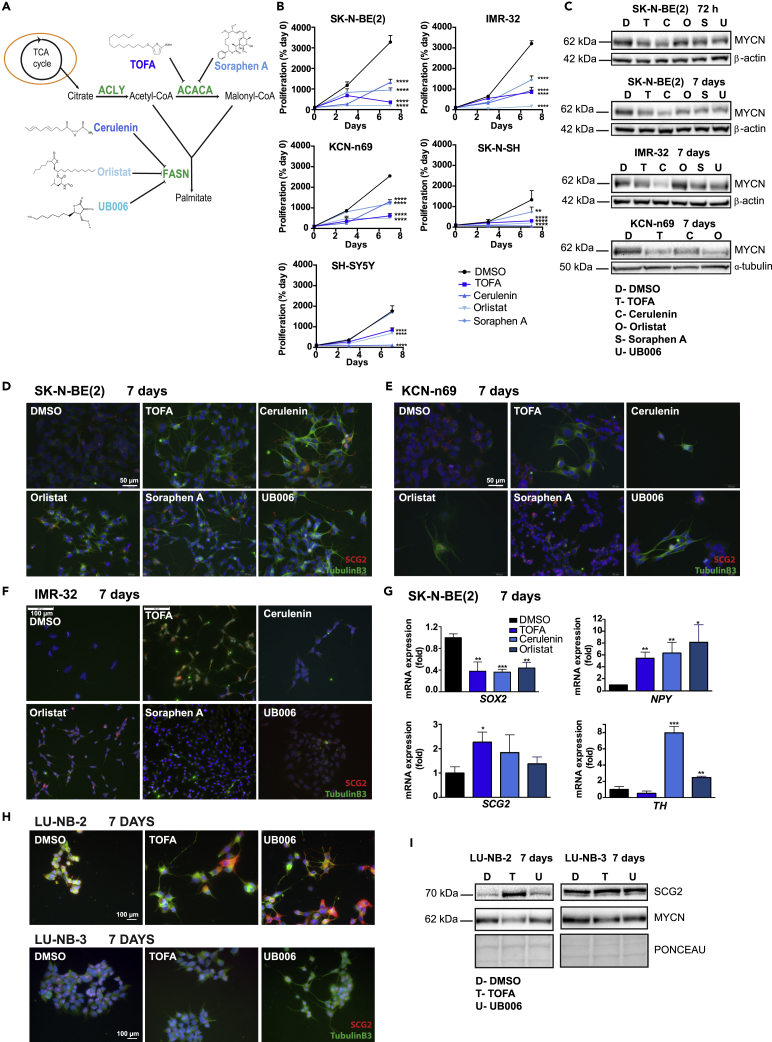
Inhibition of *de novo* fatty acid synthesis reduces cell growth and MYC expression and induces differentiation in neuroblastoma cells, related to Figure S1 and Table S1 (A) Fatty acid synthesis pathway and inhibitors used in this study. See main text for details. TCA cycle: tricarboxylic acid cycle; ACLY: ATP-citrate lyase; ACACA: acetyl-CoA carboxylase; FASN; fatty acid synthase. (B) Cell proliferation curves of the indicated *MYCN*-amplified (SK-N-BE(2), IMR-32, and KCN-n69) and non-*MYCN*-amplified (SH-SY5Y and SK-N-SH) neuroblastoma cell lines as quantified by cell counting at the indicated time points. Data are presented as mean ± SD of three independent experiments, % of day 0. (C) Western blot analysis of MYCN protein expression in the indicated cell lines and time points upon fatty acid synthesis inhibition. D: DMSO; T: TOFA; C: Cerulenin; O: Orlistat; S: Soraphen A; U: UB006. α-tubulin or β-actin were used as loading controls as indicated. (D) Immunofluorescence staining of SK-N-BE(2) cells treated for 7 days with vehicle or the indicated inhibitors. Blue: DAPI; green: Tubulin B3; red: SCG2. Scale bar indicates 50 μm. (E) Immunofluorescence staining of KCN-n69 cells treated for 7 days with vehicle or the indicated inhibitors. Blue: DAPI; green: Tubulin B3; red: SCG2. Scale bar indicates 50 μm. (F) Immunofluorescence staining of IMR-32 cells treated for 7 days with vehicle or the indicated inhibitors. Blue: DAPI; green: Tubulin B3; red: SCG2. Scale bar indicates 100 μm. (G) mRNA fold expression of the indicated genes in SK-N-BE(2) treated for 7 days with vehicle or the indicated inhibitors, as quantified by qPCR. (H) Immunofluorescence staining of PDX-derived cell cultures, LU-NB-2 and LU-NB-3, treated for 7 days with vehicle or the indicated inhibitors. Blue: DAPI; green: Tubulin B3; red: SCG2. Scale bar indicates 100 μm. (I) Western blot analysis of SCG2 and MYCN protein expression in LU-NB-2 and LU-NB-3 after 7 days upon fatty acid synthesis inhibition. (D) DMSO; T: TOFA; U: UB006. β-actin was used as loading control as indicated. Data are presented as mean ± SD of three independent experiments; statistical analysis: t test of each inhibitor compared with vehicle, with ∗, ∗∗, and ∗∗∗ indicating p < 0.05, p < 0.01, and p < 0.001, respectively. Western blot and microscopic images are representative of at least three independent experiments.

Fatty acid synthesis occurs in the cytosol and involves multiple steps (Figure 1A). The two key enzymes involved are acetyl-CoA carboxylase (ACACA) and fatty acid synthase (FASN). In the first step, ACACA synthetizes malonyl-CoA from acetyl-CoA. Malonyl-CoA is further converted into palmitate by FASN. Palmitate is subsequently elongated and desaturated by additional enzymes to produce all the other non-essential fatty acids (Nelson et al., 2017; Maier et al., 2010; Guillou et al., 2010). In the adult human, *de novo* fatty acid synthesis occurs only in the liver, adipose tissue, and lactating mammary gland. However, virtually all tumors reactivate this pathway, as well as other lipid synthesis pathways, in order to support increased proliferation, as they require lipids both as membrane components and as signaling molecules involved in stress response, cell survival, cell death, invasion, metastasis, and symbiotic relationships with the tumor stroma (Santos and Schulze, 2012; Rohrig and Schulze, 2016; Snaebjornsson et al., 2020).

The work presented here sheds light onto the relevance of the *de novo* fatty acid synthesis for neuroblastoma.

## Results

### Inhibition of *de novo* fatty acid synthesis reduces cell growth and MYC expression and results in differentiation in neuroblastoma cells

To study the relevance of fatty acid synthesis for neuroblastoma biology, we inhibited this process with the use of five small molecule inhibitors in a panel of *MYCN*-amplified (MNA) and non-*MYCN-*amplified (NMNA) neuroblastoma cells (Table S1). Two of these inhibitors, TOFA and Soraphen A, target ACACA, whereas the other three, Cerulenin, Orlistat, and UB006, target FASN (Figure 1A).

Inhibition of fatty acid synthesis resulted in reduced cell proliferation in all neuroblastoma cell lines tested (Figure 1B). When comparing the effects of fatty acid synthesis inhibition in neuroblastoma cells and human primary fibroblasts, we observed that all three neuroblastoma cell lines died at concentrations of UB006 equal to the IC_50_ of the fibroblasts (over 20 μM UB006) (Figure S1A). Western blot analysis showed increased cleavage of poly ADP-ribose polymerase (PARP) after treatment with TOFA or Cerulenin, suggesting that fatty synthesis inhibition results in apoptosis in neuroblastoma cells (Figure S1B). Cell cycle analysis by flow cytometry showed that inhibition of both ACACA and FASN induced cell death as indicated by an increase in the sub-G1 population and reduced number of cells in S/G2/M cell cycle phases at different time points (Figure S1C). The decrease in cell proliferation and increased cell death was accompanied by reduced protein expression of MYCN (in MNA cells) or c-MYC (in NMNA cells) upon treatment with all tested inhibitors (Figures 1C and S1D).

One striking effect of inhibition of *de novo* fatty acid synthesis in neuroblastoma cells was the induction of differentiation as indicated by increased neurite outgrowth and expression of neural differentiation markers. All tested inhibitors increased the protein expression of Tubulin B3 and the neurotrophic tyrosine kinase receptor type 1 (TRKA) in SK-N-BE(2) and IMR-32 cells, whereas all inhibitors except Soraphen A led to their expression in KCN-n69 cells (Figures 1D–1F). We analyzed the levels of additional markers of differentiation in SK-N-BE(2) cells treated with TOFA, Cerulenin, or Orlistat using qPCR (Figure 1G). All treatments reduced the expression of the gene encoding the neural stem cell marker SRY-box transcription factor 2 (*SOX2*) and increased the expression of the genes encoding the neural differentiation markers neuropeptide Y (*NPY*), secretogranin 2 (*SCG2*), and thyroxine hydroxylase (*TH*). Importantly, inhibition of fatty acid synthesis with TOFA or UB006 in two *MYCN*-amplified human-patient-derived xenograft (PDX)-derived cell cultures (Persson et al., 2017) also induced neurite outgrowth, expression of the differentiation markers Tubulin B3 and SCG2, and downregulation of MYCN protein (Figures 1H and 1I). In addition, TOFA, Cerulenin, and Orlistat increased protein expression of the neural differentiation marker Tubulin B3 in SH-SY5Y and SK-N-SH cells (Figure S1E). In the case of SK-N-SH and SH-EP cells, which do not undergo neural but glial differentiation, each of the treatments induced morphological changes and also augmented the levels of vimentin (Figure S1E). All neuroblastoma cells tested, except KELLY and SK-N-AS, showed morphological changes indicative of increased differentiation (Figure S1F). Notably, SK-N-AS cells do not differentiate upon treatment with the well-established differentiation agent all-trans retinoic acid (ATRA) (Gaetano et al., 1991) (Figure S1G), whereas KELLY cells to our knowledge have not been described to differentiate in any condition. Furthermore, we found increased expression of the differentiation marker SCG2 in the *ex vivo TH-MYCN* tumor sphere model (Ribeiro et al., 2016) after inhibition of fatty acid synthesis (Figures S1H and S1I).

Together our results show that inhibition of fatty acid synthesis in neuroblastoma cells leads to reduced proliferation, increased cell death, lower levels of MYC(N) proteins, and induction of neural differentiation.

### Neural differentiation is a specific consequence of diminished fatty acid synthesis and lipid withdrawal

**Figure 2:**
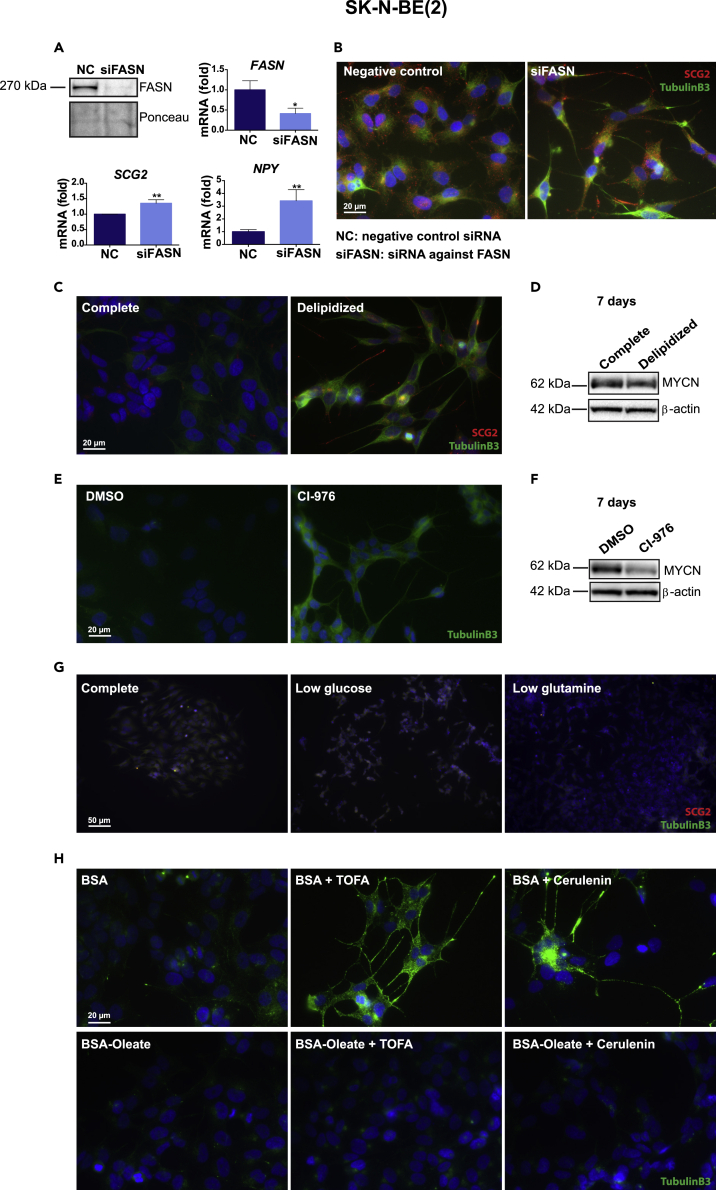
Induction of differentiation is not a general response to nutrient starvation but a specific consequence of diminished fatty acid synthesis and lipid withdrawal, related to Figure S2 (A) Upper row: FASN protein (left) and mRNA (right) levels in SK-N-BE(2) cells transfected for 72 h with negative control siRNA or siRNA against *FASN*. Lower row: quantification of the mRNA expression levels of the differentiation markers *SCG2* and *NPY* in SK-N-BE(2) cells transfected for 72 h with negative control siRNA or siRNA against *FASN*, as quantified by qPCR. (B) Immunofluorescence staining of SK-N-BE(2) cells transfected for 72 h with negative control siRNA or siRNA targeting *FASN*. Blue: DAPI; green: Tubulin B3; red: SCG2. Scale bar indicates 20 μm. (C) Immunofluorescence staining of SK-N-BE(2) cells incubated for 7 days in complete or delipidized medium. Blue: DAPI; green: Tubulin B3; red: SCG2. Scale bar indicates 20 μm. (D) Western blot analysis of MYCN protein in SK-N-BE(2) cells upon incubation in complete or delipidized medium for 7 days. (E) Immunofluorescence staining of SK-N-BE(2) cells treated for 7 days with vehicle or CI-976. Blue: DAPI; green: Tubulin B3. Scale bar indicates 20 μm. (F) Western blot analysis of MYCN protein in SK-N-BE(2) cells upon treatment with vehicle or CI-976 for 7 days. (G) Immunofluorescence staining of SK-N-BE(2) cells incubated for 7 days in complete, low glucose or low glutamine medium. Blue: DAPI; green: Tubulin B3; red: SCG2. Scale bar indicates 50 μm. (H) Immunofluorescence staining of SK-N-BE(2) cells treated for 7 days with vehicle, TOFA, or Cerulenin ± BSA/BSA-bound oleate. Blue: DAPI; green: Tubulin B3. Scale bar indicates 20 μm. Western blot and microscopic images are representative of at least three independent experiments. β-actin was used as loading control in Western blots as indicated. qPCR data are presented as mean ± SD of at least three independent experiments; statistical analysis: t test with ∗ and ∗∗ indicating p < 0.05 and p < 0.01, respectively.

To rule out the existence of off-target effects of the tested fatty acid synthesis inhibitors, we transfected SK-N-BE(2) cells with negative control or specific anti-FASN siRNA, leading to a potent reduction of both FASN protein and mRNA levels (Figure 2A). Already 72 h after transfection, the neuroblastoma cells experienced a marked increase of expression levels of the differentiation markers *SCG2* and *NPY* at mRNA levels (Figure 2A). This was accompanied by prominent neurite outgrowth and increased protein expression of the differentiation markers SCG2 and Tubulin B3 as shown by immunofluorescence (Figure 2B). These results indicate that reduced levels of FASN lead to the same phenotype as upon inhibition of fatty acid synthesis with small compounds and shows that FASN downregulation is even more potent in its ability to induce differentiation, as robust neurite outgrowth is observed after only 3 days.

Next we analyzed if reduced availability of exogenous lipids could lead to induction of differentiation. As shown in Figure 2C, incubation of SK-N-BE(2) cells in growth medium containing delipidized serum led to robust neurite outgrowth and expression of the neural differentiation markers Tubulin B3 and SCG2, together with reduced MYCN protein levels (Figure 2D). Similar to TOFA and Cerulenin, delipidized serum also led to an increase in cleaved PARP (Figure S1D). We next assessed whether interfering with the production of fatty acid-derived lipid species, such as phospholipids, had a similar impact on neuroblastoma cells. To this end, we treated SK-N-BE(2) cells with CI-976, an inhibitor of lysophospholipid acyltransferase (LPAT) and sterol O-acyltransferase (ACAT) (Figure S2A), which induced strong neural differentiation (Figure 2E) concomitant with decreased MYCN levels (Figure 2F).

We wondered whether neuroblastoma cells would undergo differentiation as a consequence of any type of nutrient starvation. To assess this, we incubated SK-N-BE(2) cells up to 7 days in growth medium with reduced glucose (1 mM versus 7.7 mM) or reduced glutamine (0.25 mM versus 2 mM). However, neither of these conditions resulted in morphological changes indicative of differentiation or in increased expression of the differentiation markers Tubulin B3 or SCG2 (Figure 2G).

To rule out the possibility that induction of differentiation upon inhibition of fatty acid synthesis could be a side effect of the chemical inhibitors used, we performed a rescue experiment in which SK-N-BE(2) cells were treated with TOFA or Cerulenin in combination with BSA or BSA-bound oleate. As shown in Figure 2H, exogenous oleate completely prevented the induction of differentiation by TOFA and Cerulenin. In addition, oleate rescued MYCN levels at 72 h of treatment but not after 7 days (Figure S2B). Neuroblastoma differentiation requires an initial increase in MYCN protein expression followed by a reduction (Guglielmi et al., 2014). Exogenous oleate partially prevented MYCN downregulation by TOFA and Cerulenin during the first days of treatment, and this might be enough to prevent differentiation induction even if MYCN levels are reduced later.

We also examined the ability of fatty acid synthesis inhibitors to potentiate all-trans retinoic acid (ATRA) induction of differentiation on NB cells. Morphologically, TOFA+ATRA- and Cerulenin+ATRA-treated cells showed fewer cell bodies and longer neurites compared with control or single-agent-treated cells (Figure S2C). TOFA+ATRA-treated cells had increased levels of Tubulin B3, whereas treatment with Cerulenin+ATRA increased the levels of SCG2 and further reduced the expression of MYCN, compared with single treatments (Figure S2D).

Collectively, our results show that neuroblastoma cells undergo differentiation upon reduced lipid synthesis or limited availability of lipids but not when subjected to restriction of other major nutrients such as glucose and glutamine.

### Inhibition of fatty acid synthesis impacts cellular lipid composition as well as mitochondrial morphology and function

**Figure 3:**
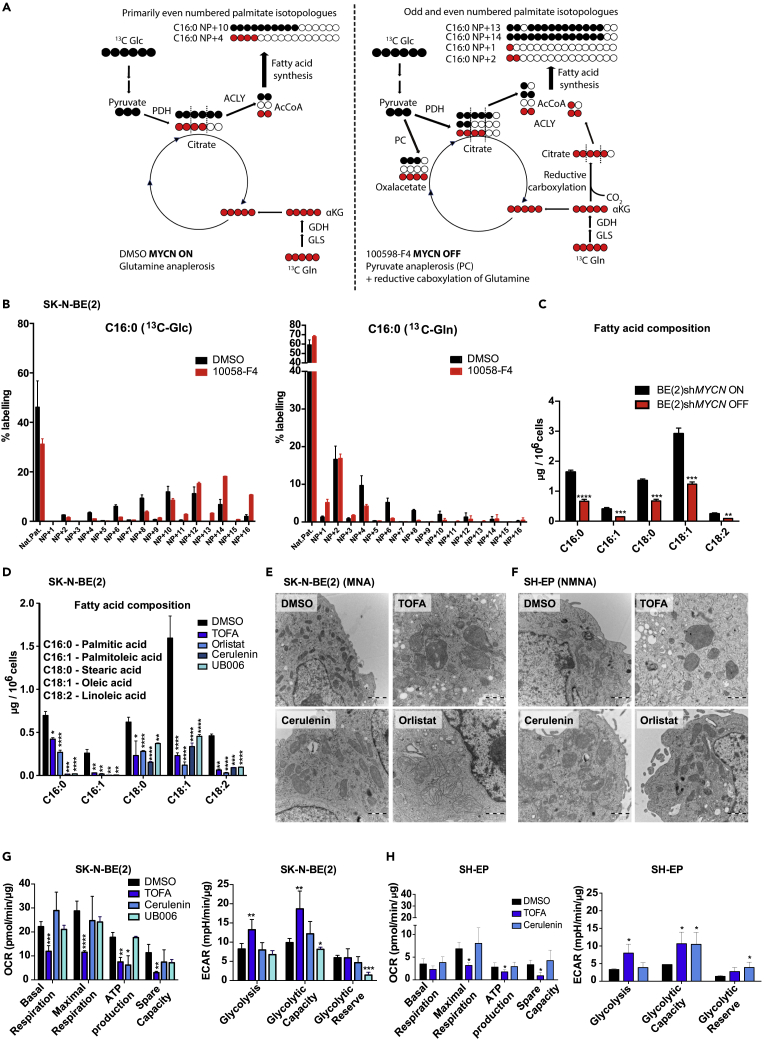
Inhibition of fatty acid synthesis has a broad impact on cellular lipid composition and affects mitochondrial morphology and function, related to Figure S3 (A) Schematic representation labeled carbon distribution from glucose and glutamine in the metabolomics experiment shown in (B). (B) Comparison of mass isotopologue distribution of palmitate was measured in SK-N-BE(2) cells treated with vehicle or the MYCN inhibitor 10058-F4 for 5 days. Glucose- and glutamine-derived carbons incorporated into palmitate were measured by metabolic tracing using U-^13^C_6_-glucose or U-^13^C_5_-glutamine as indicated. (C) Abundance of the indicated fatty acid species in SK-N-BE(2)sh*MYCN* cells treated with vehicle or doxycycline for 72 h, determined by LC. (D) Abundance of the indicated fatty acids in SK-N-BE(2) cells treated for 7 days with vehicle or the indicated fatty acid synthesis inhibitors, determined by LC. (E) Transmission electron microscopy images of representative mitochondria in SK-N-BE(2) cells treated for 7 days with vehicle or the indicated fatty acid synthesis inhibitors. Scale bars indicate 1 μm. (F) Transmission electron microscopy images of representative mitochondria in SH-EP cells treated for 7 days with vehicle or the indicated fatty acid synthesis inhibitors. Scale bars indicate 1 μm. (G) Quantification of oxygen consumption rate (OCR, left panel) and extracellular acidification rate (ECAR, right panel) in SK-N-BE(2) cells treated for 72 h with vehicle or the indicated fatty acid synthesis inhibitors. (H) Quantification of OCR (left panel) and ECAR (right panel) in SH-EP cells treated for 72 h with vehicle or the indicated fatty acid synthesis inhibitors. All data are presented as mean ± SD of at least three independent experiments; statistical analysis: t test with ∗, ∗∗, ∗∗∗, and ∗∗∗∗ indicating p < 0.05, p < 0.01, p < 0.001, and p < 0.0001, respectively. Microscopic images are representative of at least three independent experiments.

We proceeded to evaluate the impact of MYCN levels on fatty acid metabolism in neuroblastoma cells. To this end, we performed tracing experiments to follow the contribution of glucose- and glutamine-derived carbons to fatty acids in SK-N-BE(2) cells with and without inhibition of *MYCN*-expression. Figure 3A shows a schematic depiction of glucose- or glutamine-derived labeled carbon distribution into citrate, an intermediary of the tricarboxylic-acid cycle that acts also as a precursor for cytosolic production of Acetyl-CoA for fatty acid synthesis, or into palmitate. As shown in Figures 3A and 3B, MYCN downregulation by the 10058-F4 MYC-inhibitor led to increased incorporation of glucose into palmitate, whereas the incorporation of glutamine was reduced. Interestingly, there was a marked increase in the M+1 and M+3 isotopologues (NP+1 and NP+3) after MYCN inhibition in the glutamine-labeled samples. This isotopologue pattern most likely indicates that the relative contribution of glutamine to fatty acid synthesis via reductive carboxylation is increased when MYCN is inhibited, which results in odd-numbered acetyl-CoA labeling. Conversely, in the absence of MYCN inhibition, glutamine is used to fuel anaplerosis. Furthermore, odd-numbered palmitate isotopologues labeled from glucose were also increased after MYCN inhibition, indicating a higher contribution of pyruvate anaplerosis via carboxylation. This suggests that MYCN promotes glutamine- but inhibits pyruvate-dependent anaplerosis. In addition, we analyzed fatty acid composition upon *MYCN* downregulation in SK-N-BE(2)sh*MYCN* cells (Henriksen et al., 2011). Palmitic, palmitoleic, stearic, and oleic acids were all reduced to around 50% of the initial amount after 72 h of MYCN downregulation. Importantly, the essential fatty acid linoleic acid was also diminished to a similar extent, suggesting that not only reduced synthesis, but, in addition, decreased uptake of fatty acids may occur as a consequence of lower MYCN expression (Figure 3C).

We were next interested in the changes in lipid composition in neuroblastoma cells following inhibition of fatty acid synthesis. TOFA, Orlistat, Cerulenin, and UB006 led to a reduction in most of both the saturated and unsaturated fatty acids quantified (Figures 3D and S3A). Some exceptions were the long chain fatty acids docosanoic acid (C22:0), which was augmented by Cerulenin and UB006, and tetracosanoic acid (C24:0), which was increased by all treatments used (Figure S3A). TOFA and Orlistat reduced ceramide and cholesterol content (Figure S3B and S3C), whereas some sphingomyelins were unaffected and others reduced or increased (Figure S3D). Because sphingomyelins are synthesized from ceramides, these effects might reflect complex regulatory mechanisms of the sphingomyelin synthesis/degradation balance depending on the availability of substrates. Treatment of SK-N-BE(2) cells with TOFA or Cerulenin led to increased levels of free acetyl-CoA (Figure S3E), in agreement with reduced demand of this substrate for the synthesis of fatty acids. Interestingly, increased levels of acetyl-CoA did not result in higher histone acetylation; instead, inhibition of fatty acid synthesis reduced the levels of acetyl H3K9 and acetyl H3K27 already after 24 h of treatment (Figure S2F). This is in line with previous data showing that reduced histone acetylation is a consequence of the differentiation of neural progenitors into neurons, oligodendrocytes, and astrocytes (Hsieh et al., 2004).

The analysis of cellular ultrastructure upon fatty acid synthesis inhibition showed that TOFA, Cerulenin, and Orlistat treatments resulted in profound changes in mitochondrial size, structure, and/or electron density both in MNA (SK-N-BE(2)) and NMNA (SH-EP and SK-N-SH) neuroblastoma cells (Figures 3E, 3F, and S3G). Because mitochondrial structure is linked to functionality, we studied oxidative phosphorylation (OXPHOS) as well as glycolysis using the Seahorse extracellular flux analyzer after fatty acid synthesis inhibition. TOFA strongly diminished mitochondrial respiration and all associated parameters in SK-N-BE(2), SH-EP, SK-N-SH, KELLY, and IMR-32 cells and induced glycolysis in all the cell lines analyzed, except in IMR-32 cells (Figures 3G, 3H, and S3H–S3J). Cerulenin decreased ATP production in SK-N-BE(2) cells (Figure 3G), reduced all OXPHOS-related parameters in SK-N-SH (Figure S3H), but had no effect on OXPHOS either in SH-EP (Figure 3H) or KELLY cells (Figure S3I). Conversely, it induced glycolysis in both cell lines (Figures 3H and S3I). Treatment of SK-N-BE(2) cells with UB006 did not affect OXPHOS but slightly decreased the glycolytic capacity and reserve (Figure 3G). In IMR-32 cells, UB006 resulted in a reduction of all OXPHOS-related parameters as well as a small reduction in glycolysis (Figure S3J). Notably, the mitochondrial changes resulting after fatty acid inhibition do not seem to be a general feature upon differentiation, as they were not observed in SK-N-BE(2) differentiated with ATRA (Figure S3K). Furthermore, UB006 treatment led both to increased OXPHOS as well as to higher glycolysis in SK-N-BE(2) cells (Figure S3L). In summary, inhibition of fatty acid synthesis resulted in lower OXPHOS functionality with a slight increase in glycolysis, probably as a response to compensate for the reduced mitochondrial energy production, and these effects are not mimicked by ATRA, a well-known differentiation agent in neuroblastoma.

### Induction of differentiation upon fatty acid synthesis inhibition is dependent on ERK signaling

**Figure 4:**
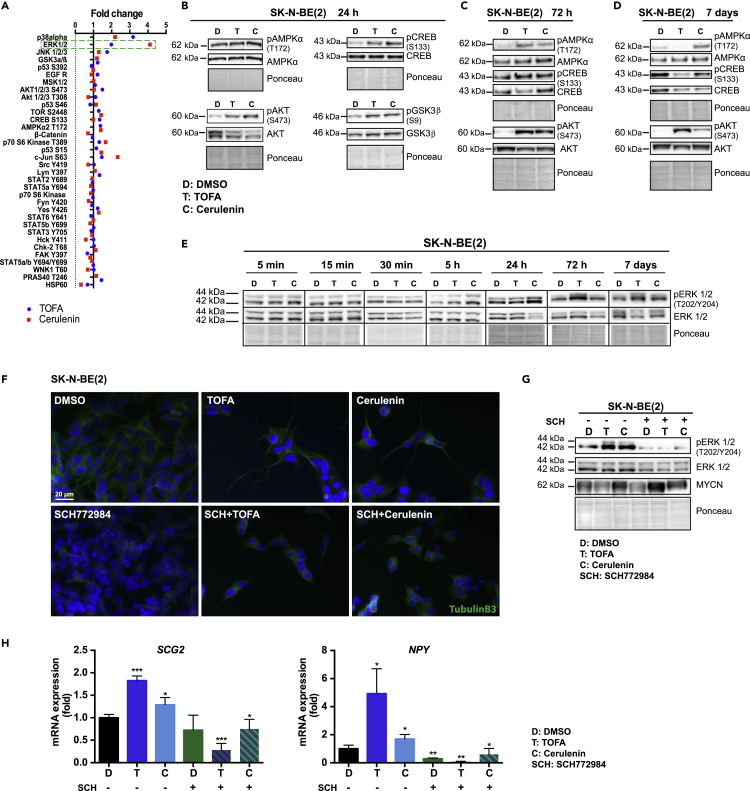
Induction of differentiation upon fatty acid synthesis inhibition is dependent on ERK signaling, related to Figure S4 (A) Fold change in phosphorylation of the indicated proteins in SK-N-BE(2) cells treated for 24 h with TOFA or Cerulenin versus vehicle, as measured by a phospho-antibody microarray. (B) Western blot analysis of the indicated proteins, in their total or phosphorylated forms, in SK-N-BE(2) cells treated for 24 h with DMSO (D), TOFA (T), or Cerulenin (C). (C) Western blot analysis of the indicated proteins, in their total or phosphorylated forms, in SK-N-BE(2) cells treated for 72 h, as indicated. (D) Western blot analysis of the indicated proteins, in their total or phosphorylated forms, in SK-N-BE(2) cells treated for 7 days, as indicated. (E) Western blot analysis at the indicated time points of total ERK or phospho-ERK in SK-N-BE(2) cells treated as indicated. (F) Immunofluorescence staining of SK-N-BE(2) cells treated for 7 days with vehicle, TOFA, or Cerulenin ± SCH772984. Blue: DAPI; green: Tubulin B3. Scale bar indicates 20 μm. (G) Western blot analysis of total and phosphorylated ERK and MYCN in SK-N-BE(2) cells treated for 72 h with DMSO (D), TOFA (T), Cerulenin (C) ± SCH772984 (S). (H) mRNA fold expression of *SCG2* and *NPY* in SK-N-BE(2) treated for 7 days with vehicle or the indicated treatments, as quantified by qPCR. Western blot and microscopic images are representative of at least three independent experiments. Ponceau staining was used as a loading control in the Western blots. qPCR data are presented as mean ± SD of at least three independent experiments; statistical analysis: t test with ∗, ∗∗, and ∗∗∗ indicating p < 0.05, p < 0.01, and p < 0.001, respectively.

To shed light into the signaling pathways activated by inhibition of fatty acid synthesis in neuroblastoma cells, we analyzed extracts of SK-N-BE(2) cells treated for 24 h with either TOFA or Cerulenin using a phospho-antibody array. ERK1/2, CREB, AMPKα, and AKT were among the most upregulated phosphorylated proteins (Figure 4A). Validation by Western blot of some of the proteins in the array using antibodies against the phosphorylated and total forms confirmed that phosphorylation was indeed increased 24 h after fatty acid synthesis inhibition (Figure 4B). At 72 h of treatment the levels of p-AKT, p-AMPKα, and p-CREB were still elevated, whereas at 7 days of inhibition, phosphorylation levels, except p-AMPKα after Cerulenin, and p-AKT after either of the two inhibitors, had descended (Figures 4C and 4D). In addition, ERK1/2 also showed increased phosphorylation at time points ranging from 24 h to 7 days in IMR-32 and SK-N-AS cells (Figures S4A–S4C). Similarly, phospho-AMPKα was increased in SH-SY5Y at both 72 h and 7 days after treatment (Figure S4D and S4E). In contrast, phosphorylation of CREB was not observed in either IMR-32 or SH-SY5Y cells (Figures S4B, S4D, and S4E).

Because of the known involvement of ERK1/2 in cell survival and differentiation (O'Neill and Kolch, 2004) and since it was one of the most strongly phosphorylated proteins in the array as well as consistently phosphorylated in different MNA and NMNA neuroblastoma cell lines treated with fatty acid synthesis inhibitors, we analyzed their phosphorylation and thus activation in more detail. Time course experiments showed that neither TOFA nor Cerulenin induced any changes in ERK1/2 phosphorylation at early time points (5, 15, and 30 min) but increased phosphorylation at around 5 hours after inhibition. The phosphorylation remained elevated in comparison with vehicle-treated cells up to 7 days of treatment (Figure 4E). These results indicate that the effect on the activation of ERK1/2 is not an immediate consequence of the addition of the inhibitors to the cells, instead it requires hours to occur, probably reflecting that this activation is triggered by the metabolic effects of the fatty acid inhibitors and not by some side effects.

To determine the potential involvement of the AKT signaling pathway in the induction of differentiation by inhibition of fatty acid synthesis, we treated SK-N-BE(2) with the AKT1/2 kinase inhibitor VIII trifluoroacetate. We observed that AKT inhibition alone, as expected, led to reduced MYCN protein levels (Ruiz-Pérez et al., 2015) and that it was unable to prevent the MYCN downregulation induced by TOFA (Figure S4F). Furthermore, AKT inhibition did not prevent the induction of neural differentiation by TOFA, evaluated both by SCG2 expression and by neurite outgrowth, whereas VIII trifluoroacetate did not have any impact on differentiation as a single agent (Figures S4F and S4G).

We next evaluated the involvement of ERK1/2 activation in the induction of differentiation. For this purpose, we treated SK-N-BE(2) cells with vehicle, TOFA, or Cerulenin alone or in combination with the ERK1/2 inhibitor SCH772984. We found that ERK1/2 inhibition prevented neural differentiation induced by fatty acid synthesis block, indicating the participation of the ERK signaling pathway in this process (Figure 4F). SCH772984 completely prevented ERK1/2 activation upon TOFA and Cerulenin treatment and rescued MYCN protein levels when combined with the fatty acid synthesis inhibitors, even though it led to a reduction in MYCN levels when used alone (Figure 4G). In agreement with these results, SCH772984 blocked the increase in mRNA levels of the differentiation markers *SCG2* and *NPY*, which are upregulated by TOFA or Cerulenin alone (Figure 4H).

Our results therefore suggest that activation of ERK signaling plays an important role in the induction of differentiation in neuroblastoma cells upon inhibition of fatty acid synthesis.

### Inhibition of fatty acid synthesis reduces neuroblastoma tumor growth and increases differentiation *in vivo*

**Figure 5:**
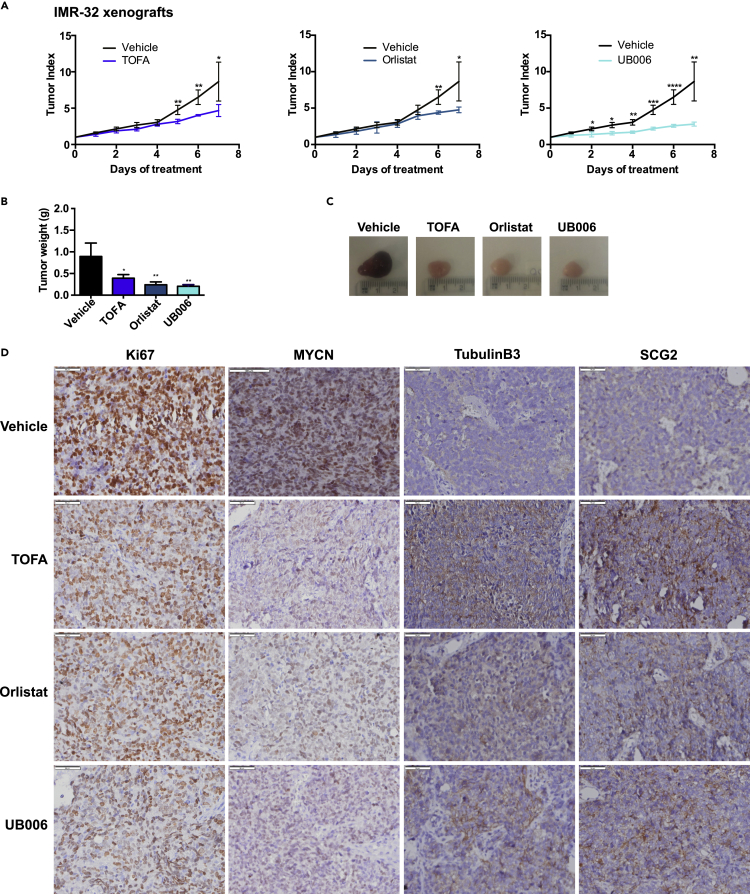
Inhibition of fatty acid synthesis reduces neuroblastoma tumor growth and increases differentiation *in vivo,* related to Figure S5 (A) Tumor index (volume each day/initial volume) of a xenograft model with IMR-32 cells. NMRI nude mice were treated daily with vehicle (10% β-cyclodextrin), 30 mg/kg TOFA, 100 mg/kg Orlistat, or 20 mg/kg UB006. n = 4 per group. (B) Tumor weight at the experiment endpoint for the tumors presented in (A). (C) Representative pictures of the IMR-32 xenograft tumors at the experimental endpoint. (D) Microscopic images of IHC staining of IMR-32 xenograft tumors labeled with anti-Ki67, anti-MYCN, anti-Tubulin B3, and anti-SCG2 anti bodies. Scale bars indicate 50 μm. Data are presented as mean ± SD. Statistical analysis: t test with ∗, ∗∗, ∗∗∗, and ∗∗∗∗ indicating p < 0.05, p < 0.01, p < 0.001, and p < 0.0001, respectively. Microscopic images are representative of at least four independent stainings per condition.

As presented earlier, inhibition of fatty acid synthesis in neuroblastoma cells *in vitro* led to reduced cell growth, increased cell death, downregulation of MYC(N), and neural differentiation. To evaluate the potential of inhibiting fatty acid synthesis *in vivo* we performed xenograft experiments in nude mice. To this end, we injected two MNA cell lines (IMR-32 and SK-N-BE(2)) and one NMNA cell line (SK-N-AS) in the right flank of nude mice. These neuroblastoma cell lines show high aggressiveness *in vivo*, giving rise to tumors that reach the maximum ethically permitted volume of 1 cm^3^ in around 6 to 8 days, thus only allowing a very short therapeutic window. IMR-32 xenografts were treated with either vehicle or 30 mg/kg TOFA (targeting ACACA), 100 mg/kg Orlistat, or 20 mg/kg UB006 (both targeting FASN). All three inhibitors reduced tumor growth as shown both by tumor volume index throughout the experiment, as well as by tumor weights at endpoint (Figures 5A–5C). After resection, paraffin sections of the tumors were stained for markers of proliferation and differentiation. We found that all treatments reduced the levels of Ki67 and MYCN, while increasing the levels of the neural differentiation markers Tubulin B3 and SCG2 (Figure 5D).

Because UB006 gave the most robust reduction on tumor growth even though it was used at lower concentrations than the other two inhibitors, we used this compound to extend the analysis to one additional MNA as well as one NMNA xenograft model. To this end we treated mice carrying tumors generated from SK-N-BE(2) and SK-N-AS cells with either vehicle or 20 mg/kg UB006. Both xenograft models showed reduced tumor volume and weight upon treatment with UB006 (Figures S5A–S5C and S5E–S5G) although not as pronounced as on the IMR-32 tumors (Figures 5A–5C). The SK-N-BE(2) tumors showed a decrease in the levels of both Ki67 and MYCN and increased expression of the differentiation markers Tubulin B3 and SCG2 (Figure S5D). Similarly, Ki67 and c-MYC were reduced in SK-N-AS tumors; however, there was no obvious increase in either Tubulin B3 or SCG2 levels (Figure S5H), in accordance with the lack of differentiation of this cell line *in vitro* upon inhibition of fatty acid synthesis (Figure S1G).

Importantly, none of the treatments generated any observable health problems nor did they affect the total body mass of any of the mice during the course of the experiment (Figure S5I), as an indication of low systemic toxicity.

Our results highlight the potential of fatty acid synthesis inhibition as a therapeutic approach for neuroblastoma irrespective of *MYCN*-status.

### Expression of *FASN* and *ACACA* correlate with poor prognosis in neuroblastoma

**Figure 6:**
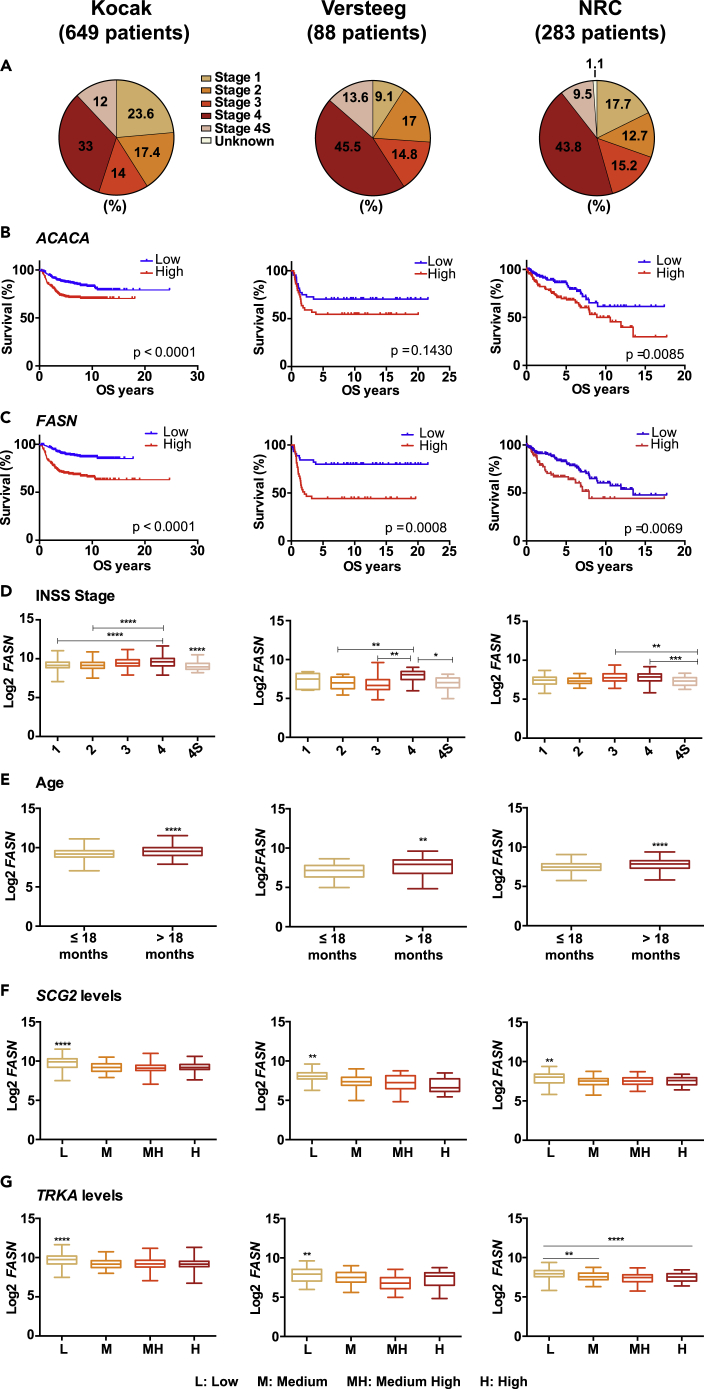
Expression of *FASN* and *ACACA* negatively correlates with good prognosis in neuroblastoma, related to Figure S6 (A) Distribution of patients in the different INSS stages in the three neuroblastoma datasets (Kocak, Versteeg, and NRC) analyzed in this study. (B) Kaplan-Meier overall survival (OS) curves for each dataset based on the mRNA expression of the *ACACA* gene. (C) Kaplan-Meier OS curves for each dataset based on the mRNA expression of the *FASN* gene. (D–G) Boxplots of *FASN* expression related to (D) stage, (E) age, (F) quartiles of *SCG2* expression, and (G) quartiles of *TRKA* expression. Statistics on boxplots for *MYCN* status and age: unpaired t test; statistics on boxplots for *TRKA*, *SCG2*, and stage: one-way ANOVA. ∗, ∗∗, ∗∗∗, and ∗∗∗∗ indicate p < 0.05, p < 0.01, p < 0.001, and p < 0.0001, respectively. p values of Mantel-Cox tests are indicated for each of the OS Figures.

To analyze the clinical relevance, we next analyzed the impact of mRNA expression levels of genes involved in fatty acid synthesis for disease outcome. To this end, we evaluated the overall survival of neuroblastoma patients in three independent datasets, referred to as Kocak (Kocak et al., 2013), Versteeg (Molenaar et al., 2012), and NRC (Neuroblastoma Research Consortium) (Rajbhandari et al., 2018). The clinical parameters of the patients in these datasets, in terms of INSS stage and *MYCN-*amplification, show similar proportions (Figure 6A and Figure S6A). High expression of both *FASN* and *ACACA* correlated to reduced overall survival in all three cohorts (Figures 6B–6C). Because both genes have been described as c-MYC targets (Gouw et al., 2019) and *FASN* also has been suggested to be a target of MYCN (Hsu et al., 2016), we analyzed the correlation of both *ACACA* and *FASN* with survival in the non-*MYCN*-amplified patients. This analysis showed that high expression levels of both genes correlated with reduced overall survival in all three datasets except for *ACACA* in the Versteeg cohort (Figures S6B and S6C). In addition, the levels of both genes were higher in MNA than in NMNA patients in the Kocak dataset (Figures S6D and S6E), whereas only *FASN* expression was elevated in MNA versus NMNA patients in the two other cohorts (Figures S6D and S6E).

We next analyzed the expression of *ACACA* and *FASN* in relation to clinical parameters with prognostic value: disease stage, age at diagnosis (worse when older than 18 months), and expression levels of *TRKA* and *SCG2,* markers of both neural differentiation and good prognosis. *FASN* was expressed at higher levels in stages 3 and 4 and reduced in stage 4S patients in all three cohorts (Figure 6D), whereas *ACACA* expression showed no significant relation to disease stage in any of the datasets (Figure S6F). Patients older than 18 months at the time of diagnosis showed significantly higher levels of expression of *FASN* in all cohorts (Figure 6E), whereas significant differences between age groups were only observed in the Versteeg dataset for *ACACA* (Figure S6G). Notably, high *FASN* expression was found in patients with low *SCG2* or *TRKA* levels in all cohorts (Figures 6F and 6G), whereas elevated *ACACA* levels were only observed in tumors with low *TRKA* in the Kocak dataset (Figure S6H).

Collectively, these data suggest that although high expression of both *ACACA* and *FASN* consistently correlates to reduced survival in tumors independently of their *MYCN* status, *FASN* seems to be a better prognosis marker candidate for neuroblastoma patients.

## Discussion

In spite of the development of more efficient therapies, high-risk neuroblastoma remains a highly deadly disease, with a survival rate of just 50%, and the harsh treatment regimens may result in reduced quality of life (Perwein et al., 2011). It is thus urgent to develop new therapeutic approaches. Although *MYCN*-amplification strongly correlates to poor clinical outcome in neuroblastoma patients, there are no MYC-targeted therapies available in the clinic to date. Targeting processes downstream of the MYC protein, such as metabolism, and most relevant for this study, lipogenesis, has been suggested as a potent strategy to overcome difficulties of directly targeting MYC (Dang et al., 2017; Gouw et al., 2019).

Tumor cells undergo changes in metabolism to sustain cell growth and division (Cantor and Sabatini, 2012). Lipids are important not only as energy sources and structural components of membranes but also as signaling molecules (Snaebjornsson et al., 2020). The activation of *de novo* fatty acid synthesis occurs in multiple cancers (Rohrig and Schulze, 2016), and its inhibition has shown promising antitumor activity in a number of tumor types, such as ovarian (Pizer et al., 1996b; Zhou et al., 2007), breast (Pizer et al., 1996a; Thupari et al., 2001; Vazquez-Martin et al., 2007; Menendez and Lupu, 2017), prostate (Pflug et al., 2003; Pizer et al., 2001), lung (Orita et al., 2007), colon (Murata et al., 2010), and others (Menendez and Lupu, 2007; Gouw et al., 2019). However, except for one study showing reduced viability of SK-N-SH cells upon treatment with Cerulenin (Slade et al., 2003), the relevance of *de novo* fatty acid synthesis for neuroblastoma has not been previously evaluated.

Using five small molecule inhibitors targeting ACACA or FASN and siRNA targeting *FASN*, we show that inhibition of fatty acid synthesis decreases cell proliferation, reduces MYCN or c-MYC protein levels, and results in neural differentiation in neuroblastoma. We have analyzed these effects in ten neuroblastoma cell lines, two neuroblastoma PDX-derived cell cultures, one *ex vivo* model system, and three xenograft models. We further validated the clinical relevance of fatty acid synthesis for neuroblastoma by analyzing gene expression data from primary neuroblastoma tumors from three independent patient datasets.

In contrast to inhibition of fatty acid β-oxidation, which is a vulnerability mainly in MNA neuroblastoma (Oliynyk et al., 2019), fatty acid synthesis can be successfully targeted in both MNA and NMNA neuroblastoma cell lines. We demonstrate that the consequences of fatty acid synthesis inhibition are specific for reduced lipid availability, as they can be rescued by exogenous oleate and because none of these effects are observed upon glucose or glutamine starvation.

Inhibition of fatty acid synthesis *in vivo*, both targeting ACACA with TOFA or FASN with either Orlistat or UB006, not only reduced tumor growth, but importantly led to neural differentiation, as assessed by expression of differentiation markers. These effects were striking especially when taking into account that these neuroblastoma xenograft models are very aggressive and only allow for a very short therapeutic window of 6 to 8 days after treatment initiation.

We found that MYCN levels and activity have a direct effect on fatty acid amount, as well as on glucose as the preferred carbon source for fatty acid synthesis in neuroblastoma, in agreement with our earlier results (Oliynyk et al., 2019) and studies in other MYC-driven tumors (Eberlin et al., 2014; Edmunds et al., 2014; Morrish et al., 2010; Gouw et al., 2019). We previously showed that MYCN downregulation leads to enlarged mitochondria with fewer cristae and reduced OXPHOS capacity (Oliynyk et al., 2019). Here we demonstrate that inhibition of fatty acid synthesis leads to a similar phenotype, most likely due to diminished MYC(N) levels. We also observed changes in cholesterol and ceramide levels. Although ceramides are fatty acid derivatives, and thus, reduced fatty acid content has a direct impact on their synthesis, cholesterol is produced in an independent pathway. However, cholesterol homeostasis is very dynamic, and changes in other lipids can affect cholesterol uptake, stabilization, and content (Mesmin and Maxfield, 2009). Interestingly, increased levels of p-AMPKα observed upon fatty acid synthesis inhibition may reflect a reduction of the energy production in the cells. Active p-AMPKα is known to inhibit biosynthetic processes, including cholesterol synthesis (Srivastava et al., 2012). These extended signaling processes may explain the effects of fatty acid synthesis inhibitors on cholesterol levels.

Our results suggest that the induction of differentiation is mediated by activation of ERK signaling, as this protein was phosphorylated in neuroblastoma cells subjected to fatty acid synthesis block, whereas cells were unable to differentiate when ERK activation was prevented. Although in the context of cancer biology, active ERK is usually associated to increased survival, proliferation, and malignancy (Samatar and Poulikakos, 2014), the RAF-MEK-ERK signaling pathway is also associated with neural differentiation under the control of the receptor for nerve growth factor (NGF), the neurotrophic tyrosine kinase receptor A (TRKA) (Khotskaya et al., 2017). Notably, inhibition of AKT, which lies downstream of the neurotrophin-3 receptor, TRKC (Khotskaya et al., 2017), did not affect the induction of differentiation mediated by fatty acid synthesis inhibition. Remarkably, agents that trigger differentiation of neuroblastoma or pheochromocytoma require the activation of several signaling pathways including ERK (Zogovic et al., 2015; Liu et al., 2015). ERK proteins can in turn phosphorylate more than six hundred different targets, including transcription factors, in a context-dependent manner (Unal et al., 2017), which explains their wide range of effects. Importantly, our *in silico* analysis showed that high *FASN* expression was found in patients with low *TRKA* levels in all three cohorts. Together, these data suggest that the TRKA-signaling pathway through ERK1/2 is activated by inhibition of fatty acid synthesis.

Importantly, we observed downregulation of MYCN/c-MYC protein levels upon inhibition of fatty acid synthesis. Among the pleiotropic functions of MYC, there is a well-stablished role in promoting proliferation and opposing differentiation both in normal physiological and cancer contexts (Dang, 2012; Yoshida, 2018). It has been reported that neuroblastoma cell differentiation requires a timely regulation of *MYCN* expression, with early overexpression (Guglielmi et al., 2014), followed by downregulation (Huang and Weiss, 2013). The downregulation of MYC proteins by inhibition of fatty acid synthesis could in this way be involved in the observed induction of neural differentiation.

Neuroblastoma is thought to originate from cells of embryonic origin blocked in an undifferentiated phenotype (Brodeur, 2003). Among all human cancers, it has the highest rate of spontaneous maturation and regression (Reynolds, 2000). This clinical observation raises the prospect that it may be possible to induce the neuroblastomas that do not spontaneously differentiate into a differentiation process by therapeutic intervention. This hypothesis has led to a search for agents able to promote neuroblastoma cell maturation. Retinoids are used for the maintenance phase of treatment, but their overall benefits are unclear (Peinemann et al., 2016; Matthay et al., 2009). Finding novel agents able to induce differentiation is an important goal in the development of new treatments for neuroblastoma.

Inhibitors of both ACACA and FASN have been available for some time (Buckley et al., 2017; Wang et al., 2015); however, very few have made their way into clinical trials, despite promising effects in preclinical studies (Rohrig and Schulze, 2016). Importantly, of the inhibitors used here, only Orlistat is FDA approved and not for cancer but for the treatment of obesity (Ameer and Weintraub, 2018). All inhibitors in this study (Figure 1A), except UB006, have been employed by others to target fatty acid synthesis *in vitro* and *in vivo* in different tumor types. UB006 is a C75-derived FASN inhibitor devoid of the anorexigenic side effects associated to the parent compound (Makowski et al., 2017). It has previously not been used for treatment of tumor-carrying mice. Here we demonstrate that UB006 efficiently decreases neuroblastoma tumor growth, showing a more potent effect than TOFA and Orlistat, without affecting animal weight. Based on these promising results, we suggest that further studies on UB006 toxicology and bioavailability are justified.

Our analysis showed that high levels of both *ACACA* and *FASN* correlate with reduced survival in three neuroblastoma patient cohorts covering all five INSS stages and with similar proportion of *MYCN-*amplification. Notably high *FASN* expression is related to low levels of neural differentiation markers and to poor prognosis factors, suggesting that it may have a prognostic value in neuroblastoma.

Our results demonstrate that inhibition of fatty acid synthesis induces differentiation in neuroblastoma independently of *MYCN*-status, providing approaches for development of more specific fatty acid synthesis inhibitors that can be used in the clinic.

### Limitations of the study

Our xenograft models are performed in immunocompromised mice. Future studies should analyze the influence of the environment, including the immune compartment, on the effects of fatty acid synthesis inhibition in neuroblastoma tumors. In addition, the cell lines used to perform the xenograft experiments show a very fast and aggressive growth to form tumors, limiting the therapeutic window to a range of 6 to 8 days. Both limitations could be overcome by using the transgenic *TH-MYCN* neuroblastoma mouse model, which possesses an intact immune system and spontaneously develops neuroblastoma tumors that highly resemble the characteristics of human neuroblastoma.

### Resource availability

#### Lead contact

Further information and requests for resources and reagents should be directed to and will be fulfilled by the Lead Contact, Marie Arsenian Henriksson (marie.arsenian.henriksson@ki.se).

#### Materials availability

This study did not generate new unique reagents.

#### Data and code availability

This study did not generate datasets or code.

## Methods

All methods can be found in the accompanying Transparent Methods supplemental file.

## Supplemental Information

Document S1. Transparent methods, Figures S1–S6, and Table S1
