# Supplementary material for: Inhibition of fatty acid synthesis induces differentiation and reduces tumor burden in childhood neuroblastoma
Source: iScience. 2021 Feb 1;24(2):102128. doi: 10.1016/j.isci.2021.102128 (PMC7895756; doi:10.1016/j.isci.2021.102128)
Supplement: Document S1. Transparent methods, Figures S1–S6, and Table S1 [file mmc1.pdf]

iScience

CellPress
OPEN ACCESS

Article

Inhibition of fatty acid synthesis induces differentiation and reduces tumor burden in childhood neuroblastoma

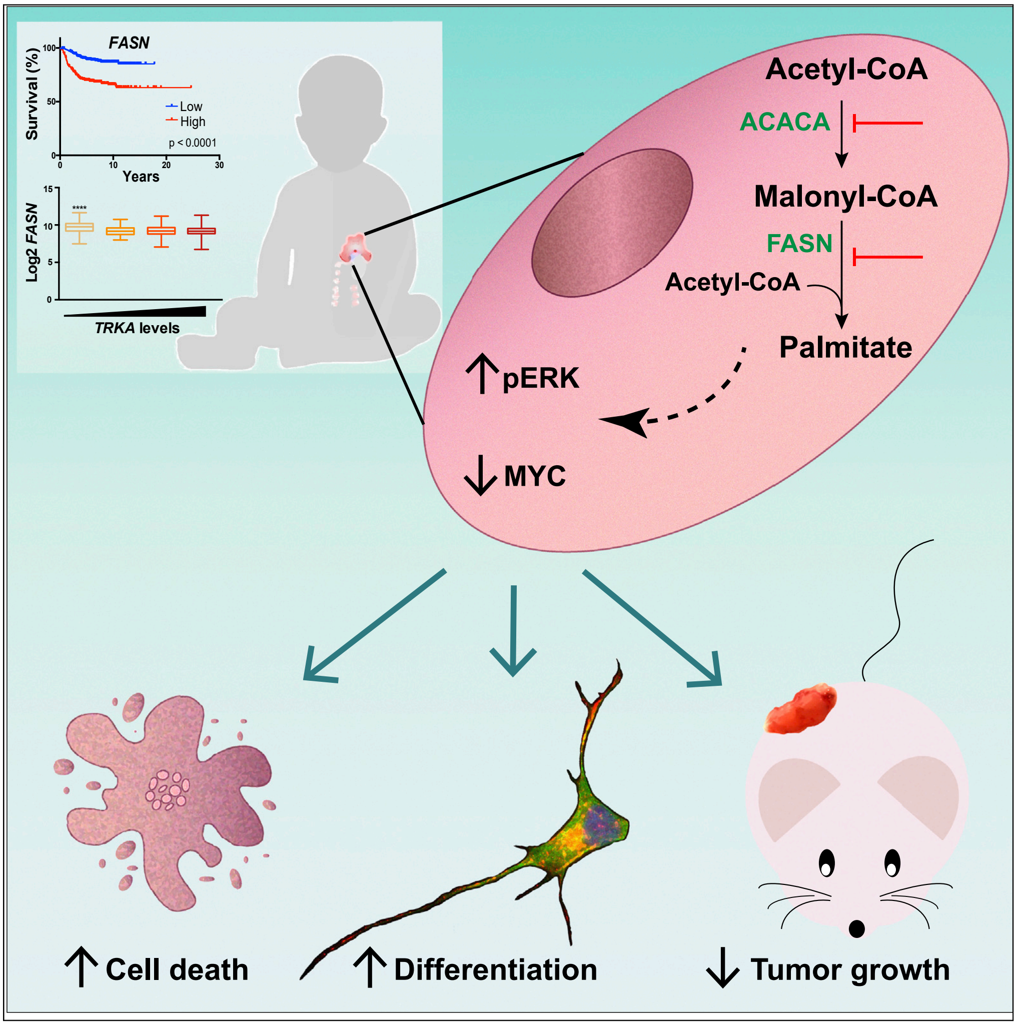

María Victoria Ruiz-Pérez, Lourdes Sainero-Alcolado, Ganna Oliynyk, ..., Jonas Bergquist, Almut Schulze, Marie Arsenian-Henriksson

maria.ruiz.perez@ki.se (M.V.R.-P.)
marie.arsenian.henriksson@ki.se (M.A.-H.)

HIGHLIGHTS

Fatty acid synthesis inhibition reduces neuroblastoma growth *in vitro* and *in vivo*

Decreased availability or reduced lipid synthesis downregulates MYC levels

Impaired fatty acid synthesis induces neural differentiation through ERK activation

High expression of fatty-acid-synthesis-related genes correlates with bad prognosis

Ruiz-Pérez et al., iScience *24*, 102128
February 19, 2021 © 2021 The Author(s).
https://doi.org/10.1016/j.isci.2021.102128

Check for updates

iScience

CellPress
OPEN ACCESS

Article

Inhibition of fatty acid synthesis induces differentiation and reduces tumor burden in childhood neuroblastoma

María Victoria Ruiz-Pérez,[1,*] Lourdes Sainero-Alcolado,[1] Ganna Oliynyk,[1] Isabell Matuschek,[1] Nicola Balboni,[1] S.J. Kumari A. Ubhayasekera,[2] Marteinn Thor Snaebjornsson,[3] Kamil Makowski,[4] Kristina Aaltonen,[5] Daniel Bexell,[5] Dolors Serra,[6] Roland Nilsson,[7,8,9] Jonas Bergquist,[2] Almut Schulze,[3] and Marie Arsenian-Henriksson[1,10,*]

[1]Department of Microbiology, Tumor and Cell Biology (MTC), Biomedicum B7, Karolinska Institutet, 171 65 Stockholm, Sweden

[2]Analytical Chemistry, Department of Chemistry and Science for Life Laboratory, Uppsala University, 751 24 Uppsala, Sweden

[3]Tumor Metabolism and Microenvironment, German Cancer Research Center (DKFZ), 69120 Heidelberg, Germany

[4]Department of Inorganic and Organic Chemistry, Section of Organic Chemistry, Faculty of Chemistry, University of Barcelona, 08028 Barcelona, Spain

[5]Translational Cancer Research, Lund University, 22381 Lund, Sweden

[6]Department of Biochemistry and Physiology, School of Pharmacy, Institute of Biomedicine of the University of Barcelona (IBUB), University of Barcelona, 08028 Barcelona, Spain, and CIBER Physiopathology of Obesity and Nutrition (CIBEROBN), Institute of Health Carlos III, 28029 Madrid, Spain

[7]Cardiovascular Medicine Unit, Department of Medicine, Karolinska Institutet, 17176 Stockholm, Sweden

[8]Division of Cardiovascular Medicine, Karolinska University Hospital, 17176 Stockholm, Sweden

[9]Center for Molecular Medicine, Karolinska Institutet, 17176 Stockholm, Sweden

[10]Lead contact

*Correspondence: maria.ruiz.perez@ki.se (M.V.R.-P.), marie.arsenian.henriksson@ki.se (M.A.-H.)

https://doi.org/10.1016/j.isci.2021.102128

SUMMARY

Many metabolic pathways, including lipid metabolism, are rewired in tumors to support energy and biomass production and to allow adaptation to stressful environments. Neuroblastoma is the second deadliest solid tumor in children. Genetic aberrations, as the amplification of the *MYCN*-oncogene, correlate strongly with disease progression. Yet, there are only a few molecular targets successfully exploited in the clinic. Here we show that inhibition of fatty acid synthesis led to increased neural differentiation and reduced tumor burden in neuroblastoma xenograft experiments independently of *MYCN*-status. This was accompanied by reduced levels of the MYCN or c-MYC oncoproteins and activation of ERK signaling. Importantly, the expression levels of genes involved in *de novo* fatty acid synthesis showed prognostic value for neuroblastoma patients. Our findings demonstrate that inhibition of *de novo* fatty acid synthesis is a promising pharmacological intervention strategy for the treatment of neuroblastoma independently of *MYCN*-status.

INTRODUCTION

Neuroblastoma is the second most common solid tumor in children and accounts for up to 15% of cancer-related deaths during childhood (Maris, 2010). It develops in the adrenal gland and the peripheral nervous system from neural crest-derived precursors of the sympathetic nervous system. Neuroblastoma presentation is highly heterogeneous, both in terms of biological features and clinical response. According to the International Neuroblastoma Staging System (INSS), patients are classified into five different stages (1–4 and 4S). Although stage 4 patients have the lowest survival probability, 4S tumors frequently undergo spontaneous regression and the children thus have a good prognosis (Ikeda et al., 2002). In addition, neuroblastoma patients are classified into three risk groups: high, medium, and low. Patients with low-risk neuroblastomas undergo little or no clinical intervention, showing a survival probability of 90%–95%. In contrast, high-risk patients have an event-free survival probability of less than 50% (Whittle et al., 2017), suffer tumor recurrence and metastasis, and develop treatment resistance leading to a fatal outcome.

Amplification of *MYCN* is used as a diagnostic parameter to classify neuroblastoma patients into stages and risk groups. *MYCN*-amplified neuroblastomas are always considered as high risk. In fact, *MYCN* amplification, occurring in around 25% of all primary neuroblastomas and in 30%–40% of all high-risk cases (Pugh et al., 2013; Huang and Weiss, 2013), has since long been known to correlate to poor clinical outcome (Brodeur et al., 1984). Both *MYCN* and *c-MYC* belong to the *MYC* oncogene family. It has been shown that MYCN promotes pluripotency and blocks differentiation pathways (Ruiz-Perez et al., 2017). Importantly, induction of differentiation in neuroblastoma cells requires early *MYCN* expression (Guglielmi et al., 2014), followed by downregulation (Huang and Weiss, 2013).

Although c-MYC is involved in the control of multiple metabolic processes, including lipid metabolism (Eberlin et al., 2014; Edmunds et al., 2014; Morrish et al., 2010; Gouw et al., 2019; Carroll et al., 2018; Dejure and Eilers, 2017), the role of MYCN in metabolism is less clear. It has been shown to drive increased

This is an open access article under the CC BY license (http://creativecommons.org/licenses/by/4.0/).

glutathione biosynthesis (Carter et al., 2016) and the overexpression of the glutamine transporter ASCT2, whose levels correlate to bad prognosis in neuroblastoma patients (Ren et al., 2015). *MYCN* overexpression, in cooperation with MondoA, led to increased levels of proteins involved in lipid metabolism, whereas MYCN and MondoA inactivation was connected to reduced *de novo* fatty acid synthesis (Carroll et al., 2015). MYCN drives changes in mitochondrial fusion, morphology, and function related to apoptosis resistance and to metabolic processes (Casinelli et al., 2016; Zirath et al., 2013; Oliynyk et al., 2019). Our group recently showed that MYCN promotes glycolysis, oxidative metabolism, and *de novo* glutamine synthesis. Importantly, neuroblastoma highly relies on fatty acid oxidation for energy production, and inhibition of β-oxidation leads to reduced growth of *MYCN*-amplified, but not of non-*MYCN*-amplified, cells and tumors (Oliynyk et al., 2019).

Fatty acid synthesis occurs in the cytosol and involves multiple steps (Figure 1A). The two key enzymes involved are acetyl-CoA carboxylase (ACACA) and fatty acid synthase (FASN). In the first step, ACACA synthetizes malonyl-CoA from acetyl-CoA. Malonyl-CoA is further converted into palmitate by FASN. Palmitate is subsequently elongated and desaturated by additional enzymes to produce all the other non-essential fatty acids (Nelson et al., 2017; Maier et al., 2010; Guillou et al., 2010). In the adult human, *de novo* fatty acid synthesis occurs only in the liver, adipose tissue, and lactating mammary gland. However, virtually all tumors reactivate this pathway, as well as other lipid synthesis pathways, in order to support increased proliferation, as they require lipids both as membrane components and as signaling molecules involved in stress response, cell survival, cell death, invasion, metastasis, and symbiotic relationships with the tumor stroma (Santos and Schulze, 2012; Rohrig and Schulze, 2016; Snaebjornsson et al., 2020).

The work presented here sheds light onto the relevance of the *de novo* fatty acid synthesis for neuroblastoma.

RESULTS

Inhibition of *de novo* fatty acid synthesis reduces cell growth and MYC expression and results in differentiation in neuroblastoma cells

To study the relevance of fatty acid synthesis for neuroblastoma biology, we inhibited this process with the use of five small molecule inhibitors in a panel of *MYCN*-amplified (MNA) and non-*MYCN*-amplified (NMNA) neuroblastoma cells (Table S1). Two of these inhibitors, TOFA and Soraphen A, target ACACA, whereas the other three, Cerulenin, Orlistat, and UB006, target FASN (Figure 1A).

Inhibition of fatty acid synthesis resulted in reduced cell proliferation in all neuroblastoma cell lines tested (Figure 1B). When comparing the effects of fatty acid synthesis inhibition in neuroblastoma cells and human primary fibroblasts, we observed that all three neuroblastoma cell lines died at concentrations of UB006 equal to the IC_{50} of the fibroblasts (over 20 μM UB006) (Figure S1A). Western blot analysis showed increased cleavage of poly ADP-ribose polymerase (PARP) after treatment with TOFA or Cerulenin, suggesting that fatty synthesis inhibition results in apoptosis in neuroblastoma cells (Figure S1B). Cell cycle analysis by flow cytometry showed that inhibition of both ACACA and FASN induced cell death as indicated by an increase in the sub-G1 population and reduced number of cells in S/G2/M cell cycle phases at different time points (Figure S1C). The decrease in cell proliferation and increased cell death was accompanied by reduced protein expression of MYCN (in MNA cells) or c-MYC (in NMNA cells) upon treatment with all tested inhibitors (Figures 1C and S1D).

One striking effect of inhibition of *de novo* fatty acid synthesis in neuroblastoma cells was the induction of differentiation as indicated by increased neurite outgrowth and expression of neural differentiation markers. All tested inhibitors increased the protein expression of Tubulin B3 and the neurotrophic tyrosine kinase receptor type 1 (TRKA) in SK-N-BE(2) and IMR-32 cells, whereas all inhibitors except Soraphen A led to their expression in KCN-n69 cells (Figures 1D–1F). We analyzed the levels of additional markers of differentiation in SK-N-BE(2) cells treated with TOFA, Cerulenin, or Orlistat using qPCR (Figure 1G). All treatments reduced the expression of the gene encoding the neural stem cell marker SRY-box transcription factor 2 (*SOX2*) and increased the expression of the genes encoding the neural differentiation markers neuropeptide Y (*NPY*), secretogranin 2 (*SCG2*), and thyroxine hydroxylase (*TH*). Importantly, inhibition of fatty acid synthesis with TOFA or UB006 in two *MYCN*-amplified human-patient-derived xenograft (PDX)-derived cell cultures (Persson et al., 2017) also induced neurite outgrowth, expression of the differentiation markers Tubulin B3 and SCG2, and downregulation of MYCN protein (Figures 1H and 1I). In

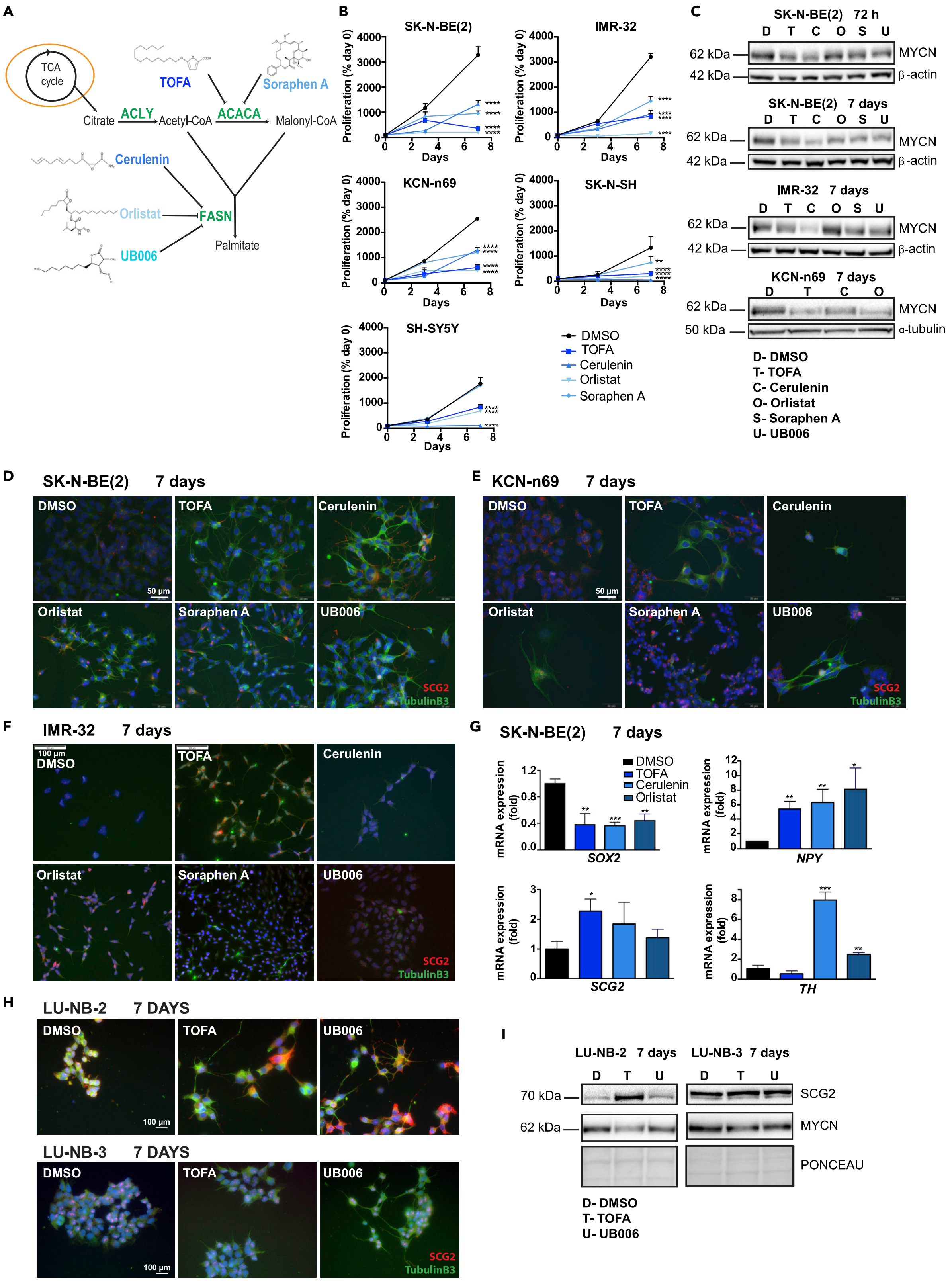
A
TCA cycle
Citrate
ACLY
Acetyl-CoA
ACACA
Malonyl-CoA
TOFA
Soraphen A
Cerulenin
Orlistat
FASN
UB006
Palmitate
B
SK-N-BE(2)
IMR-32
KCN-n69
SK-N-SH
SH-SY5Y
Proliferation (% day 0)
Days
DMSO
TOFA
Cerulenin
Orlistat
Soraphen A
C
SK-N-BE(2) 72 h
SK-N-BE(2) 7 days
IMR-32 7 days
KCN-n69 7 days
D T C O S U
62 kDa
42 kDa
50 kDa
MYCN
β-actin
α-tubulin
D- DMSO
T- TOFA
C- Cerulenin
O- Orlistat
S- Soraphen A
U- UB006
D
SK-N-BE(2) 7 days
E
KCN-n69 7 days
DMSO
TOFA
Cerulenin
Orlistat
Soraphen A
UB006
SCG2
TubulinB3
50 µm
F
IMR-32 7 days
100 µm
G
SK-N-BE(2) 7 days
mRNA expression (fold)
SOX2
NPY
SCG2
TH
H
LU-NB-2 7 DAYS
LU-NB-3 7 DAYS
I
LU-NB-2 7 days
LU-NB-3 7 days
D T U
70 kDa
SCG2
MYCN
PONCEAU
D- DMSO
T- TOFA
U- UB006

Figure 1. Inhibition of *de novo* fatty acid synthesis reduces cell growth and MYC expression and induces differentiation in neuroblastoma cells, related to Figure S1 and Table S1

(A) Fatty acid synthesis pathway and inhibitors used in this study. See main text for details. TCA cycle: tricarboxylic acid cycle; ACLY: ATP-citrate lyase; ACACA: acetyl-CoA carboxylase; FASN; fatty acid synthase.

(B) Cell proliferation curves of the indicated *MYCN*-amplified (SK-N-BE(2), IMR-32, and KCN-n69) and non-*MYCN*-amplified (SH-SY5Y and SK-N-SH) neuroblastoma cell lines as quantified by cell counting at the indicated time points. Data are presented as mean ± SD of three independent experiments, % of day 0.

(C) Western blot analysis of MYCN protein expression in the indicated cell lines and time points upon fatty acid synthesis inhibition. D: DMSO; T: TOFA; C: Cerulenin; O: Orlistat; S: Soraphen A; U: UB006. α-tubulin or β-actin were used as loading controls as indicated.

(D) Immunofluorescence staining of SK-N-BE(2) cells treated for 7 days with vehicle or the indicated inhibitors. Blue: DAPI; green: Tubulin B3; red: SCG2. Scale bar indicates 50 μm.

(E) Immunofluorescence staining of KCN-n69 cells treated for 7 days with vehicle or the indicated inhibitors. Blue: DAPI; green: Tubulin B3; red: SCG2. Scale bar indicates 50 μm.

(F) Immunofluorescence staining of IMR-32 cells treated for 7 days with vehicle or the indicated inhibitors. Blue: DAPI; green: Tubulin B3; red: SCG2. Scale bar indicates 100 μm.

(G) mRNA fold expression of the indicated genes in SK-N-BE(2) treated for 7 days with vehicle or the indicated inhibitors, as quantified by qPCR.

(H) Immunofluorescence staining of PDX-derived cell cultures, LU-NB-2 and LU-NB-3, treated for 7 days with vehicle or the indicated inhibitors. Blue: DAPI; green: Tubulin B3; red: SCG2. Scale bar indicates 100 μm.

(I) Western blot analysis of SCG2 and MYCN protein expression in LU-NB-2 and LU-NB-3 after 7 days upon fatty acid synthesis inhibition. (D) DMSO; T: TOFA; U: UB006. β-actin was used as loading control as indicated.

Data are presented as mean ± SD of three independent experiments; statistical analysis: t test of each inhibitor compared with vehicle, with *, **, and *** indicating $p < 0.05$, $p < 0.01$, and $p < 0.001$, respectively. Western blot and microscopic images are representative of at least three independent experiments.

addition, TOFA, Cerulenin, and Orlistat increased protein expression of the neural differentiation marker Tubulin B3 in SH-SY5Y and SK-N-SH cells (Figure S1E). In the case of SK-N-SH and SH-EP cells, which do not undergo neural but glial differentiation, each of the treatments induced morphological changes and also augmented the levels of vimentin (Figure S1E). All neuroblastoma cells tested, except KELLY and SK-N-AS, showed morphological changes indicative of increased differentiation (Figure S1F). Notably, SK-N-AS cells do not differentiate upon treatment with the well-established differentiation agent all-trans retinoic acid (ATRA) (Gaetano et al., 1991) (Figure S1G), whereas KELLY cells to our knowledge have not been described to differentiate in any condition. Furthermore, we found increased expression of the differentiation marker SCG2 in the *ex vivo TH-MYCN* tumor sphere model (Ribeiro et al., 2016) after inhibition of fatty acid synthesis (Figures S1H and S1I).

Together our results show that inhibition of fatty acid synthesis in neuroblastoma cells leads to reduced proliferation, increased cell death, lower levels of MYC(N) proteins, and induction of neural differentiation.

Neural differentiation is a specific consequence of diminished fatty acid synthesis and lipid withdrawal

To rule out the existence of off-target effects of the tested fatty acid synthesis inhibitors, we transfected SK-N-BE(2) cells with negative control or specific anti-FASN siRNA, leading to a potent reduction of both FASN protein and mRNA levels (Figure 2A). Already 72 h after transfection, the neuroblastoma cells experienced a marked increase of expression levels of the differentiation markers *SCG2* and *NPY* at mRNA levels (Figure 2A). This was accompanied by prominent neurite outgrowth and increased protein expression of the differentiation markers SCG2 and Tubulin B3 as shown by immunofluorescence (Figure 2B). These results indicate that reduced levels of FASN lead to the same phenotype as upon inhibition of fatty acid synthesis with small compounds and shows that FASN downregulation is even more potent in its ability to induce differentiation, as robust neurite outgrowth is observed after only 3 days.

Next we analyzed if reduced availability of exogenous lipids could lead to induction of differentiation. As shown in Figure 2C, incubation of SK-N-BE(2) cells in growth medium containing delipidized serum led to robust neurite outgrowth and expression of the neural differentiation markers Tubulin B3 and SCG2, together with reduced MYCN protein levels (Figure 2D). Similar to TOFA and Cerulenin, delipidized serum also led to an increase in cleaved PARP (Figure S1D). We next assessed whether interfering with the production of fatty acid-derived lipid species, such as phospholipids, had a similar impact on neuroblastoma cells. To this end, we treated SK-N-BE(2) cells with CI-976, an inhibitor of lysophospholipid acyltransferase (LPAT) and sterol O-acyltransferase (ACAT) (Figure S2A), which induced strong neural differentiation (Figure 2E) concomitant with decreased MYCN levels (Figure 2F).

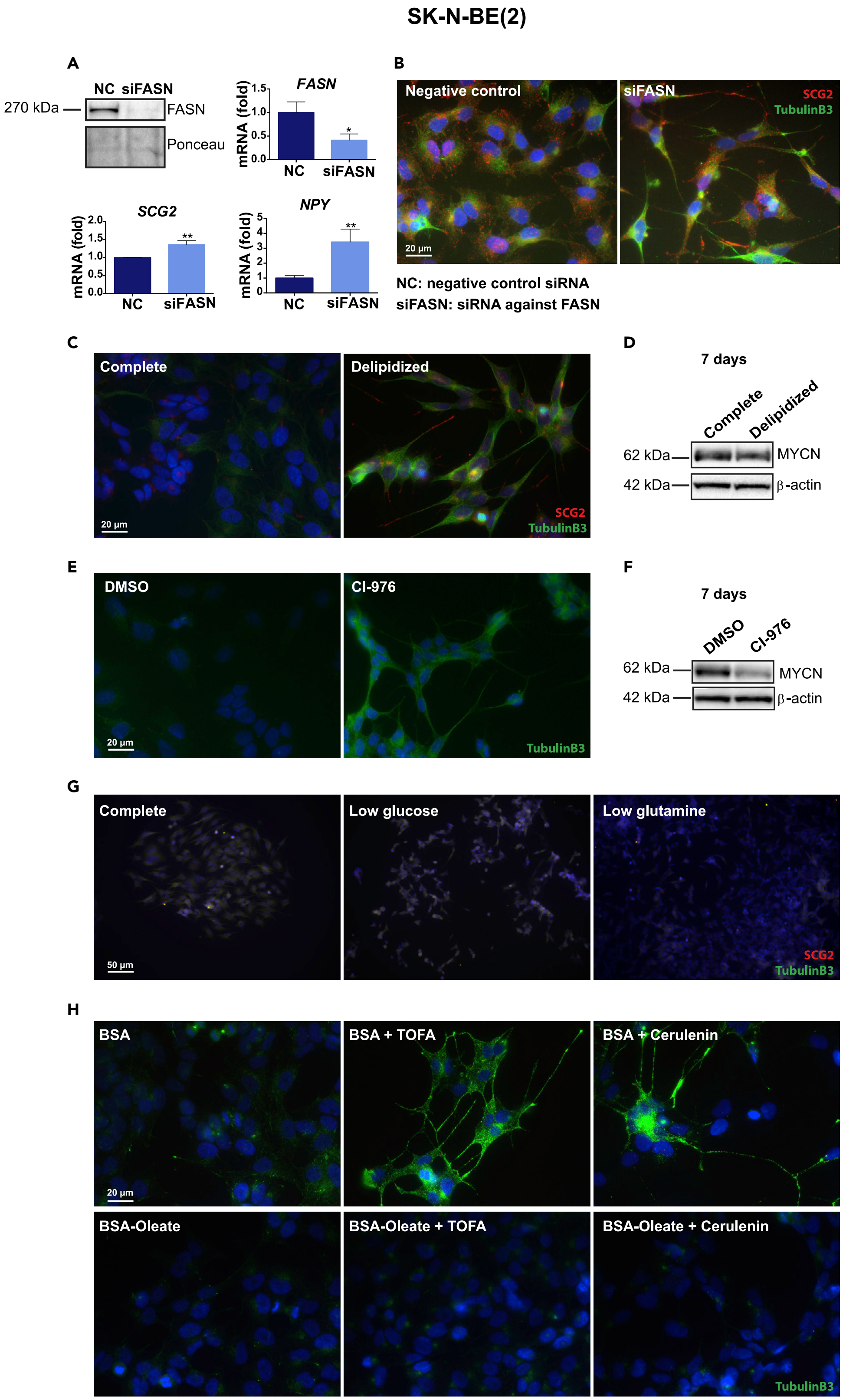
SK-N-BE(2)
A
NC siFASN
270 kDa
FASN
Ponceau
FASN
mRNA (fold)
NC siFASN
SCG2
NPY
B
Negative control
siFASN
SCG2
TubulinB3
20 µm
NC: negative control siRNA
siFASN: siRNA against FASN
C
Complete
Delipidized
D
7 days
62 kDa
MYCN
42 kDa
β-actin
E
DMSO
CI-976
F
G
Low glucose
Low glutamine
50 µm
H
BSA
BSA + TOFA
BSA + Cerulenin
BSA-Oleate
BSA-Oleate + TOFA
BSA-Oleate + Cerulenin

Figure 2. Induction of differentiation is not a general response to nutrient starvation but a specific consequence of diminished fatty acid synthesis and lipid withdrawal, related to Figure S2

(A) Upper row: FASN protein (left) and mRNA (right) levels in SK-N-BE(2) cells transfected for 72 h with negative control siRNA or siRNA against *FASN*. Lower row: quantification of the mRNA expression levels of the differentiation markers *SCG2* and *NPY* in SK-N-BE(2) cells transfected for 72 h with negative control siRNA or siRNA against *FASN*, as quantified by qPCR.

(B) Immunofluorescence staining of SK-N-BE(2) cells transfected for 72 h with negative control siRNA or siRNA targeting *FASN*. Blue: DAPI; green: Tubulin B3; red: SCG2. Scale bar indicates 20 μm.

(C) Immunofluorescence staining of SK-N-BE(2) cells incubated for 7 days in complete or delipidized medium. Blue: DAPI; green: Tubulin B3; red: SCG2. Scale bar indicates 20 μm.

(D) Western blot analysis of MYCN protein in SK-N-BE(2) cells upon incubation in complete or delipidized medium for 7 days.

(E) Immunofluorescence staining of SK-N-BE(2) cells treated for 7 days with vehicle or CI-976. Blue: DAPI; green: Tubulin B3. Scale bar indicates 20 μm.

(F) Western blot analysis of MYCN protein in SK-N-BE(2) cells upon treatment with vehicle or CI-976 for 7 days.

(G) Immunofluorescence staining of SK-N-BE(2) cells incubated for 7 days in complete, low glucose or low glutamine medium. Blue: DAPI; green: Tubulin B3; red: SCG2. Scale bar indicates 50 μm.

(H) Immunofluorescence staining of SK-N-BE(2) cells treated for 7 days with vehicle, TOFA, or Cerulenin ± BSA/BSA-bound oleate. Blue: DAPI; green: Tubulin B3. Scale bar indicates 20 μm.

Western blot and microscopic images are representative of at least three independent experiments. β-actin was used as loading control in Western blots as indicated. qPCR data are presented as mean ± SD of at least three independent experiments; statistical analysis: t test with * and ** indicating $p < 0.05$ and $p < 0.01$, respectively.

We wondered whether neuroblastoma cells would undergo differentiation as a consequence of any type of nutrient starvation. To assess this, we incubated SK-N-BE(2) cells up to 7 days in growth medium with reduced glucose (1 mM versus 7.7 mM) or reduced glutamine (0.25 mM versus 2 mM). However, neither of these conditions resulted in morphological changes indicative of differentiation or in increased expression of the differentiation markers Tubulin B3 or SCG2 (Figure 2G).

To rule out the possibility that induction of differentiation upon inhibition of fatty acid synthesis could be a side effect of the chemical inhibitors used, we performed a rescue experiment in which SK-N-BE(2) cells were treated with TOFA or Cerulenin in combination with BSA or BSA-bound oleate. As shown in Figure 2H, exogenous oleate completely prevented the induction of differentiation by TOFA and Cerulenin. In addition, oleate rescued MYCN levels at 72 h of treatment but not after 7 days (Figure S2B). Neuroblastoma differentiation requires an initial increase in MYCN protein expression followed by a reduction (Guglielmi et al., 2014). Exogenous oleate partially prevented MYCN downregulation by TOFA and Cerulenin during the first days of treatment, and this might be enough to prevent differentiation induction even if MYCN levels are reduced later.

We also examined the ability of fatty acid synthesis inhibitors to potentiate all-trans retinoic acid (ATRA) induction of differentiation on NB cells. Morphologically, TOFA+ATRA- and Cerulenin+ATRA-treated cells showed fewer cell bodies and longer neurites compared with control or single-agent-treated cells (Figure S2C). TOFA+ATRA-treated cells had increased levels of Tubulin B3, whereas treatment with Cerulenin+ATRA increased the levels of SCG2 and further reduced the expression of MYCN, compared with single treatments (Figure S2D).

Collectively, our results show that neuroblastoma cells undergo differentiation upon reduced lipid synthesis or limited availability of lipids but not when subjected to restriction of other major nutrients such as glucose and glutamine.

Inhibition of fatty acid synthesis impacts cellular lipid composition as well as mitochondrial morphology and function

We proceeded to evaluate the impact of MYCN levels on fatty acid metabolism in neuroblastoma cells. To this end, we performed tracing experiments to follow the contribution of glucose- and glutamine-derived carbons to fatty acids in SK-N-BE(2) cells with and without inhibition of *MYCN*-expression. Figure 3A shows a schematic depiction of glucose- or glutamine-derived labeled carbon distribution into citrate, an intermediary of the tricarboxylic-acid cycle that acts also as a precursor for cytosolic production of Acetyl-CoA for fatty acid synthesis, or into palmitate. As shown in Figures 3A and 3B, MYCN downregulation by the 10058-F4 MYC-inhibitor led to increased incorporation of glucose into palmitate, whereas the

CellPress
OPEN ACCESS

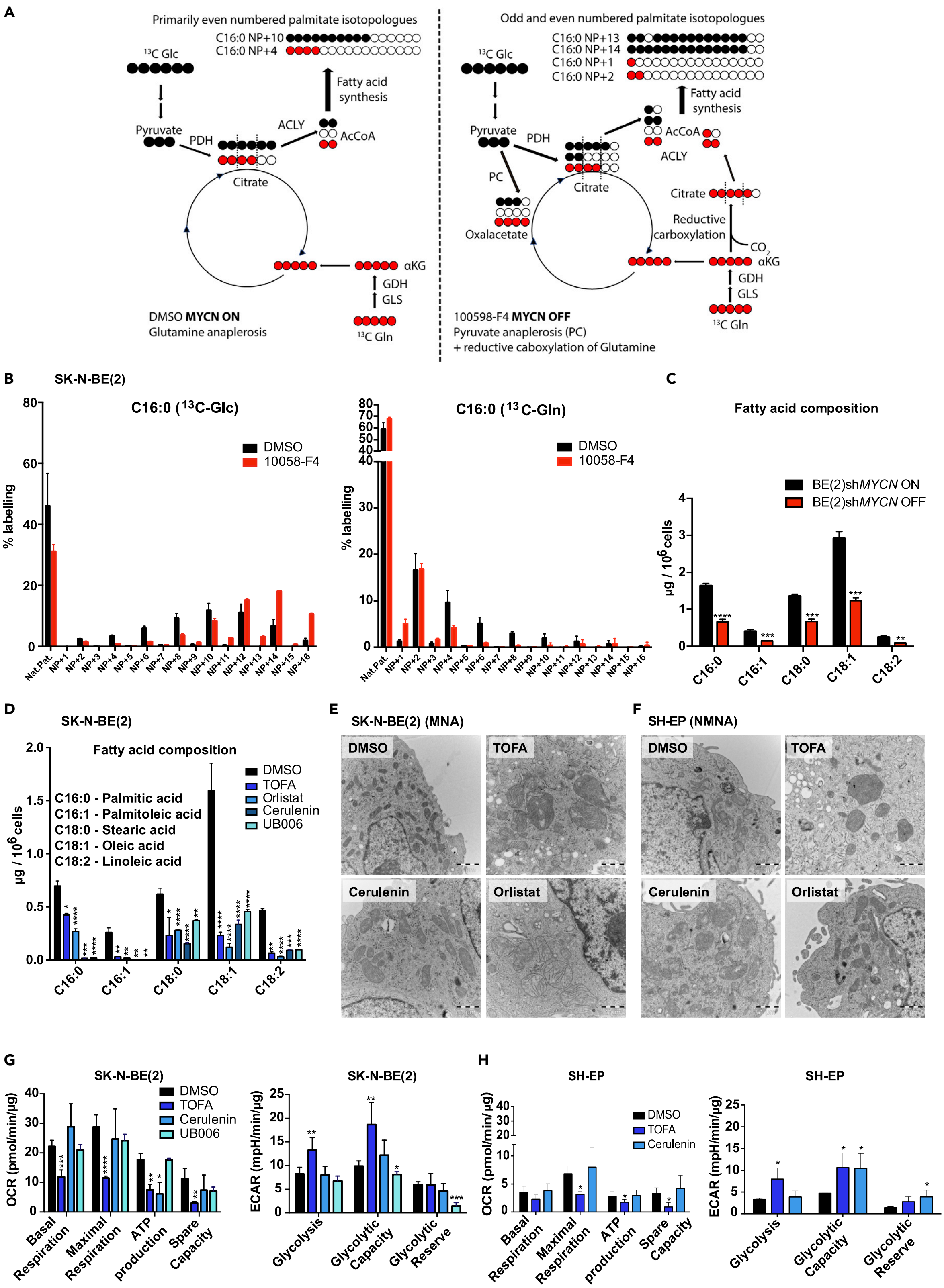

Figure 3. Inhibition of fatty acid synthesis has a broad impact on cellular lipid composition and affects mitochondrial morphology and function, related to Figure S3

(A) Schematic representation labeled carbon distribution from glucose and glutamine in the metabolomics experiment shown in (B).

(B) Comparison of mass isotopologue distribution of palmitate was measured in SK-N-BE(2) cells treated with vehicle or the MYCN inhibitor 10058-F4 for 5 days. Glucose- and glutamine-derived carbons incorporated into palmitate were measured by metabolic tracing using U-$^{13}C_6$-glucose or U-$^{13}C_5$-glutamine as indicated.

(C) Abundance of the indicated fatty acid species in SK-N-BE(2)sh*MYCN* cells treated with vehicle or doxycycline for 72 h, determined by LC.

(D) Abundance of the indicated fatty acids in SK-N-BE(2) cells treated for 7 days with vehicle or the indicated fatty acid synthesis inhibitors, determined by LC.

(E) Transmission electron microscopy images of representative mitochondria in SK-N-BE(2) cells treated for 7 days with vehicle or the indicated fatty acid synthesis inhibitors. Scale bars indicate 1 μm.

(F) Transmission electron microscopy images of representative mitochondria in SH-EP cells treated for 7 days with vehicle or the indicated fatty acid synthesis inhibitors. Scale bars indicate 1 μm.

(G) Quantification of oxygen consumption rate (OCR, left panel) and extracellular acidification rate (ECAR, right panel) in SK-N-BE(2) cells treated for 72 h with vehicle or the indicated fatty acid synthesis inhibitors.

(H) Quantification of OCR (left panel) and ECAR (right panel) in SH-EP cells treated for 72 h with vehicle or the indicated fatty acid synthesis inhibitors.

All data are presented as mean ± SD of at least three independent experiments; statistical analysis: t test with *, **, ***, and **** indicating $p < 0.05$, $p < 0.01$, $p < 0.001$, and $p < 0.0001$, respectively. Microscopic images are representative of at least three independent experiments.

incorporation of glutamine was reduced. Interestingly, there was a marked increase in the M+1 and M+3 isotopologues (NP+1 and NP+3) after MYCN inhibition in the glutamine-labeled samples. This isotopologue pattern most likely indicates that the relative contribution of glutamine to fatty acid synthesis via reductive carboxylation is increased when MYCN is inhibited, which results in odd-numbered acetyl-CoA labeling. Conversely, in the absence of MYCN inhibition, glutamine is used to fuel anaplerosis. Furthermore, odd-numbered palmitate isotopologues labeled from glucose were also increased after MYCN inhibition, indicating a higher contribution of pyruvate anaplerosis via carboxylation. This suggests that MYCN promotes glutamine- but inhibits pyruvate-dependent anaplerosis. In addition, we analyzed fatty acid composition upon *MYCN* downregulation in SK-N-BE(2)sh*MYCN* cells (Henriksen et al., 2011). Palmitic, palmitoleic, stearic, and oleic acids were all reduced to around 50% of the initial amount after 72 h of MYCN downregulation. Importantly, the essential fatty acid linoleic acid was also diminished to a similar extent, suggesting that not only reduced synthesis, but, in addition, decreased uptake of fatty acids may occur as a consequence of lower MYCN expression (Figure 3C).

We were next interested in the changes in lipid composition in neuroblastoma cells following inhibition of fatty acid synthesis. TOFA, Orlistat, Cerulenin, and UB006 led to a reduction in most of both the saturated and unsaturated fatty acids quantified (Figures 3D and S3A). Some exceptions were the long chain fatty acids docosanoic acid (C22:0), which was augmented by Cerulenin and UB006, and tetracosanoic acid (C24:0), which was increased by all treatments used (Figure S3A). TOFA and Orlistat reduced ceramide and cholesterol content (Figure S3B and S3C), whereas some sphingomyelins were unaffected and others reduced or increased (Figure S3D). Because sphingomyelins are synthesized from ceramides, these effects might reflect complex regulatory mechanisms of the sphingomyelin synthesis/degradation balance depending on the availability of substrates. Treatment of SK-N-BE(2) cells with TOFA or Cerulenin led to increased levels of free acetyl-CoA (Figure S3E), in agreement with reduced demand of this substrate for the synthesis of fatty acids. Interestingly, increased levels of acetyl-CoA did not result in higher histone acetylation; instead, inhibition of fatty acid synthesis reduced the levels of acetyl H3K9 and acetyl H3K27 already after 24 h of treatment (Figure S2F). This is in line with previous data showing that reduced histone acetylation is a consequence of the differentiation of neural progenitors into neurons, oligodendrocytes, and astrocytes (Hsieh et al., 2004).

The analysis of cellular ultrastructure upon fatty acid synthesis inhibition showed that TOFA, Cerulenin, and Orlistat treatments resulted in profound changes in mitochondrial size, structure, and/or electron density both in MNA (SK-N-BE(2)) and NMNA (SH-EP and SK-N-SH) neuroblastoma cells (Figures 3E, 3F, and S3G). Because mitochondrial structure is linked to functionality, we studied oxidative phosphorylation (OXPHOS) as well as glycolysis using the Seahorse extracellular flux analyzer after fatty acid synthesis inhibition. TOFA strongly diminished mitochondrial respiration and all associated parameters in SK-N-BE(2), SH-EP, SK-N-SH, KELLY, and IMR-32 cells and induced glycolysis in all the cell lines analyzed, except in IMR-32 cells (Figures 3G, 3H, and S3H–S3J). Cerulenin decreased ATP production in SK-N-BE(2) cells (Figure 3G), reduced all OXPHOS-related parameters in SK-N-SH (Figure S3H), but had no effect on OXPHOS either in SH-EP (Figure 3H) or KELLY cells (Figure S3I). Conversely, it induced glycolysis in both cell lines (Figures 3H and S3I). Treatment of SK-N-BE(2) cells with UB006 did not affect OXPHOS but slightly decreased the glycolytic

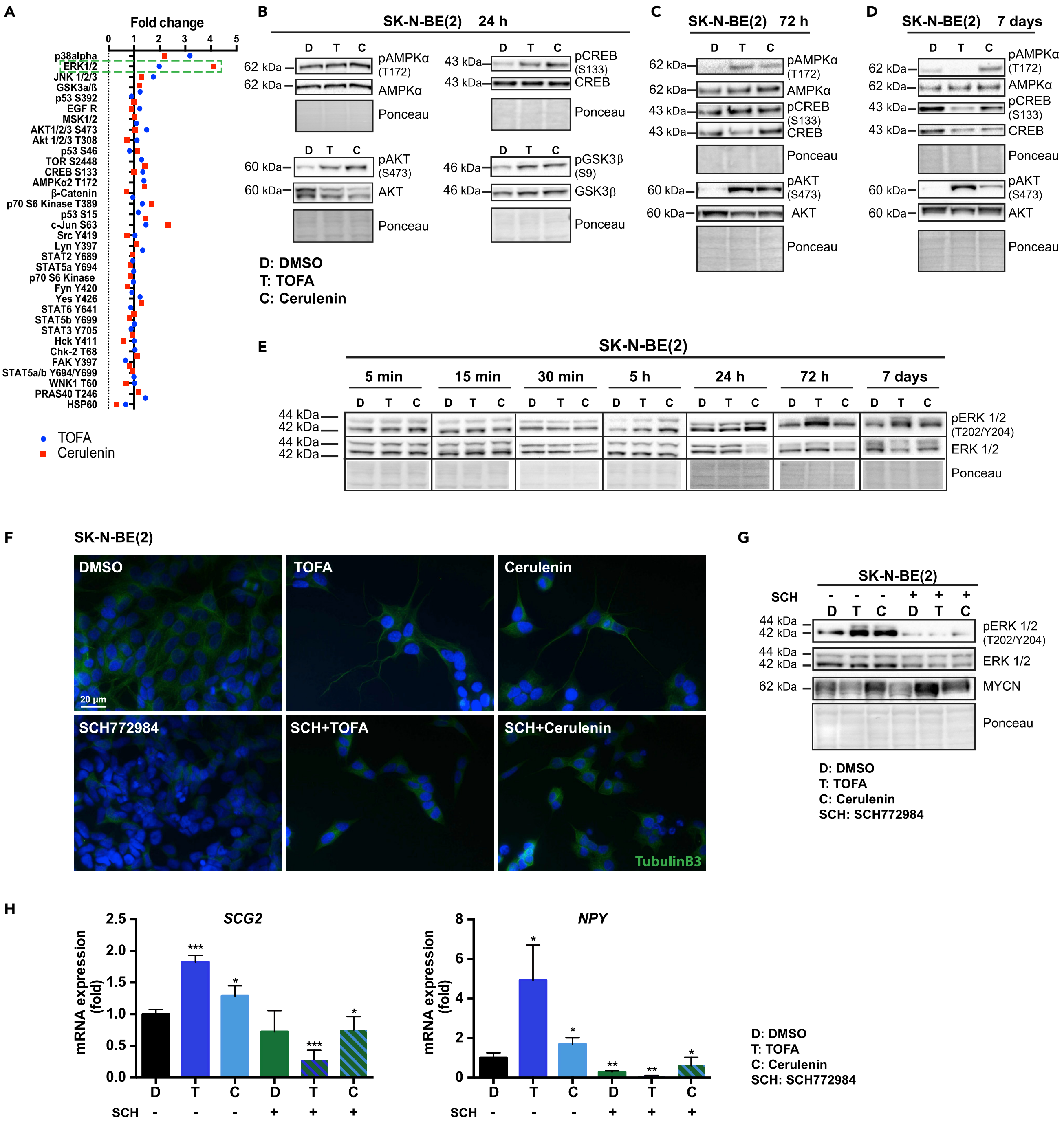

Figure 4. Induction of differentiation upon fatty acid synthesis inhibition is dependent on ERK signaling, related to Figure S4

(A) Fold change in phosphorylation of the indicated proteins in SK-N-BE(2) cells treated for 24 h with TOFA or Cerulenin versus vehicle, as measured by a phospho-antibody microarray.

(B) Western blot analysis of the indicated proteins, in their total or phosphorylated forms, in SK-N-BE(2) cells treated for 24 h with DMSO (D), TOFA (T), or Cerulenin (C).

(C) Western blot analysis of the indicated proteins, in their total or phosphorylated forms, in SK-N-BE(2) cells treated for 72 h, as indicated.

(D) Western blot analysis of the indicated proteins, in their total or phosphorylated forms, in SK-N-BE(2) cells treated for 7 days, as indicated.

(E) Western blot analysis at the indicated time points of total ERK or phospho-ERK in SK-N-BE(2) cells treated as indicated.

(F) Immunofluorescence staining of SK-N-BE(2) cells treated for 7 days with vehicle, TOFA, or Cerulenin ± SCH772984. Blue: DAPI; green: Tubulin B3. Scale bar indicates 20 μm.

Figure 4. *Continued*

(G) Western blot analysis of total and phosphorylated ERK and MYCN in SK-N-BE(2) cells treated for 72 h with DMSO (D), TOFA (T), Cerulenin (C) ± SCH772984 (S).

(H) mRNA fold expression of *SCG2* and *NPY* in SK-N-BE(2) treated for 7 days with vehicle or the indicated treatments, as quantified by qPCR. Western blot and microscopic images are representative of at least three independent experiments. Ponceau staining was used as a loading control in the Western blots. qPCR data are presented as mean ± SD of at least three independent experiments; statistical analysis: t test with *, **, and *** indicating $p < 0.05$, $p < 0.01$, and $p < 0.001$, respectively.

capacity and reserve (Figure 3G). In IMR-32 cells, UB006 resulted in a reduction of all OXPHOS-related parameters as well as a small reduction in glycolysis (Figure S3J). Notably, the mitochondrial changes resulting after fatty acid inhibition do not seem to be a general feature upon differentiation, as they were not observed in SK-N-BE(2) differentiated with ATRA (Figure S3K). Furthermore, UB006 treatment led both to increased OXPHOS as well as to higher glycolysis in SK-N-BE(2) cells (Figure S3L). In summary, inhibition of fatty acid synthesis resulted in lower OXPHOS functionality with a slight increase in glycolysis, probably as a response to compensate for the reduced mitochondrial energy production, and these effects are not mimicked by ATRA, a well-known differentiation agent in neuroblastoma.

Induction of differentiation upon fatty acid synthesis inhibition is dependent on ERK signaling

To shed light into the signaling pathways activated by inhibition of fatty acid synthesis in neuroblastoma cells, we analyzed extracts of SK-N-BE(2) cells treated for 24 h with either TOFA or Cerulenin using a phospho-antibody array. ERK1/2, CREB, AMPKα, and AKT were among the most upregulated phosphorylated proteins (Figure 4A). Validation by Western blot of some of the proteins in the array using antibodies against the phosphorylated and total forms confirmed that phosphorylation was indeed increased 24 h after fatty acid synthesis inhibition (Figure 4B). At 72 h of treatment the levels of p-AKT, p-AMPKα, and p-CREB were still elevated, whereas at 7 days of inhibition, phosphorylation levels, except p-AMPKα after Cerulenin, and p-AKT after either of the two inhibitors, had descended (Figures 4C and 4D). In addition, ERK1/2 also showed increased phosphorylation at time points ranging from 24 h to 7 days in IMR-32 and SK-N-AS cells (Figures S4A–S4C). Similarly, phospho-AMPKα was increased in SH-SY5Y at both 72 h and 7 days after treatment (Figure S4D and S4E). In contrast, phosphorylation of CREB was not observed in either IMR-32 or SH-SY5Y cells (Figures S4B, S4D, and S4E).

Because of the known involvement of ERK1/2 in cell survival and differentiation (O'Neill and Kolch, 2004) and since it was one of the most strongly phosphorylated proteins in the array as well as consistently phosphorylated in different MNA and NMNA neuroblastoma cell lines treated with fatty acid synthesis inhibitors, we analyzed their phosphorylation and thus activation in more detail. Time course experiments showed that neither TOFA nor Cerulenin induced any changes in ERK1/2 phosphorylation at early time points (5, 15, and 30 min) but increased phosphorylation at around 5 hours after inhibition. The phosphorylation remained elevated in comparison with vehicle-treated cells up to 7 days of treatment (Figure 4E). These results indicate that the effect on the activation of ERK1/2 is not an immediate consequence of the addition of the inhibitors to the cells, instead it requires hours to occur, probably reflecting that this activation is triggered by the metabolic effects of the fatty acid inhibitors and not by some side effects.

To determine the potential involvement of the AKT signaling pathway in the induction of differentiation by inhibition of fatty acid synthesis, we treated SK-N-BE(2) with the AKT1/2 kinase inhibitor VIII trifluoroacetate. We observed that AKT inhibition alone, as expected, led to reduced MYCN protein levels (Ruiz-Pérez et al., 2015) and that it was unable to prevent the MYCN downregulation induced by TOFA (Figure S4F). Furthermore, AKT inhibition did not prevent the induction of neural differentiation by TOFA, evaluated both by SCG2 expression and by neurite outgrowth, whereas VIII trifluoroacetate did not have any impact on differentiation as a single agent (Figures S4F and S4G).

We next evaluated the involvement of ERK1/2 activation in the induction of differentiation. For this purpose, we treated SK-N-BE(2) cells with vehicle, TOFA, or Cerulenin alone or in combination with the ERK1/2 inhibitor SCH772984. We found that ERK1/2 inhibition prevented neural differentiation induced by fatty acid synthesis block, indicating the participation of the ERK signaling pathway in this process (Figure 4F). SCH772984 completely prevented ERK1/2 activation upon TOFA and Cerulenin treatment and rescued MYCN protein levels when combined with the fatty acid synthesis inhibitors, even though it led to a reduction in MYCN levels when used alone (Figure 4G). In agreement with these results,

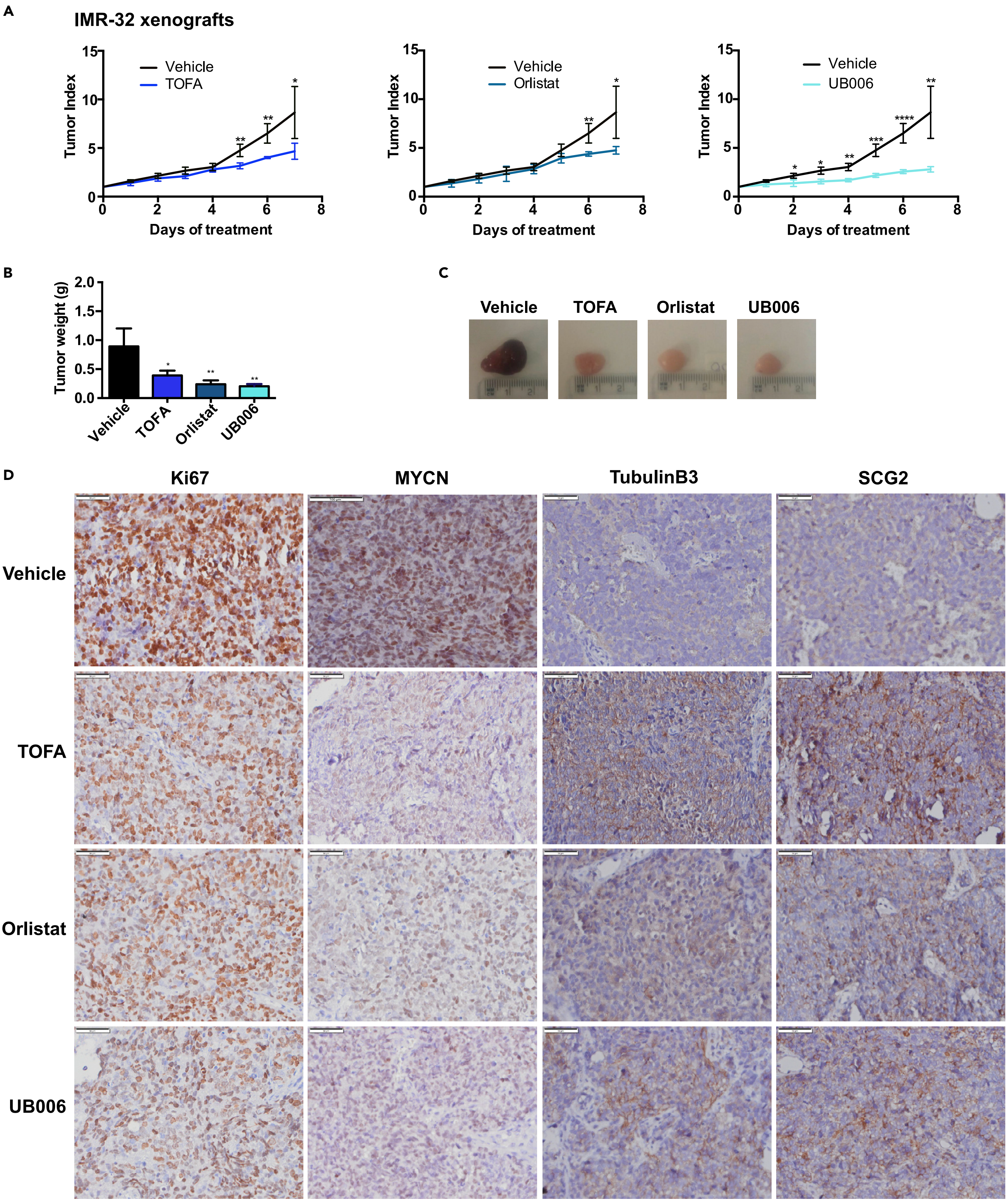

Figure 5. Inhibition of fatty acid synthesis reduces neuroblastoma tumor growth and increases differentiation *in vivo*, related to Figure S5

(A) Tumor index (volume each day/initial volume) of a xenograft model with IMR-32 cells. NMRI nude mice were treated daily with vehicle (10% β-cyclodextrin), 30 mg/kg TOFA, 100 mg/kg Orlistat, or 20 mg/kg UB006. n = 4 per group.

(B) Tumor weight at the experiment endpoint for the tumors presented in (A).

Figure 5. *Continued*

(C) Representative pictures of the IMR-32 xenograft tumors at the experimental endpoint.

(D) Microscopic images of IHC staining of IMR-32 xenograft tumors labeled with anti-Ki67, anti-MYCN, anti-Tubulin B3, and anti-SCG2 anti bodies. Scale bars indicate 50 μm.

Data are presented as mean ± SD. Statistical analysis: t test with *, **, ***, and **** indicating $p < 0.05$, $p < 0.01$, $p < 0.001$, and $p < 0.0001$, respectively. Microscopic images are representative of at least four independent stainings per condition.

SCH772984 blocked the increase in mRNA levels of the differentiation markers *SCG2* and *NPY*, which are upregulated by TOFA or Cerulenin alone (Figure 4H).

Our results therefore suggest that activation of ERK signaling plays an important role in the induction of differentiation in neuroblastoma cells upon inhibition of fatty acid synthesis.

Inhibition of fatty acid synthesis reduces neuroblastoma tumor growth and increases differentiation *in vivo*

As presented earlier, inhibition of fatty acid synthesis in neuroblastoma cells *in vitro* led to reduced cell growth, increased cell death, downregulation of MYC(N), and neural differentiation. To evaluate the potential of inhibiting fatty acid synthesis *in vivo* we performed xenograft experiments in nude mice. To this end, we injected two MNA cell lines (IMR-32 and SK-N-BE(2)) and one NMNA cell line (SK-N-AS) in the right flank of nude mice. These neuroblastoma cell lines show high aggressiveness *in vivo*, giving rise to tumors that reach the maximum ethically permitted volume of 1 cm^3 in around 6 to 8 days, thus only allowing a very short therapeutic window. IMR-32 xenografts were treated with either vehicle or 30 mg/kg TOFA (targeting ACACA), 100 mg/kg Orlistat, or 20 mg/kg UB006 (both targeting FASN). All three inhibitors reduced tumor growth as shown both by tumor volume index throughout the experiment, as well as by tumor weights at endpoint (Figures 5A–5C). After resection, paraffin sections of the tumors were stained for markers of proliferation and differentiation. We found that all treatments reduced the levels of Ki67 and MYCN, while increasing the levels of the neural differentiation markers Tubulin B3 and SCG2 (Figure 5D).

Because UB006 gave the most robust reduction on tumor growth even though it was used at lower concentrations than the other two inhibitors, we used this compound to extend the analysis to one additional MNA as well as one NMNA xenograft model. To this end we treated mice carrying tumors generated from SK-N-BE(2) and SK-N-AS cells with either vehicle or 20 mg/kg UB006. Both xenograft models showed reduced tumor volume and weight upon treatment with UB006 (Figures S5A–S5C and S5E–S5G) although not as pronounced as on the IMR-32 tumors (Figures 5A–5C). The SK-N-BE(2) tumors showed a decrease in the levels of both Ki67 and MYCN and increased expression of the differentiation markers Tubulin B3 and SCG2 (Figure S5D). Similarly, Ki67 and c-MYC were reduced in SK-N-AS tumors; however, there was no obvious increase in either Tubulin B3 or SCG2 levels (Figure S5H), in accordance with the lack of differentiation of this cell line *in vitro* upon inhibition of fatty acid synthesis (Figure S1G).

Importantly, none of the treatments generated any observable health problems nor did they affect the total body mass of any of the mice during the course of the experiment (Figure S5I), as an indication of low systemic toxicity.

Our results highlight the potential of fatty acid synthesis inhibition as a therapeutic approach for neuroblastoma irrespective of *MYCN*-status.

Expression of *FASN* and *ACACA* correlate with poor prognosis in neuroblastoma

To analyze the clinical relevance, we next analyzed the impact of mRNA expression levels of genes involved in fatty acid synthesis for disease outcome. To this end, we evaluated the overall survival of neuroblastoma patients in three independent datasets, referred to as Kocak (Kocak et al., 2013), Versteeg (Molenaar et al., 2012), and NRC (Neuroblastoma Research Consortium) (Rajbhandari et al., 2018). The clinical parameters of the patients in these datasets, in terms of INSS stage and *MYCN*-amplification, show similar proportions (Figure 6A and Figure S6A). High expression of both *FASN* and *ACACA* correlated to reduced overall survival in all three cohorts (Figures 6B–6C). Because both genes have been described as c-MYC targets (Gouw et al., 2019) and *FASN* also has been suggested to be a target of MYCN (Hsu et al., 2016), we analyzed the correlation of both *ACACA* and *FASN* with survival in the non-*MYCN*-amplified patients. This analysis showed that high expression levels of both genes correlated with reduced overall survival in all three

CellPress
OPEN ACCESS

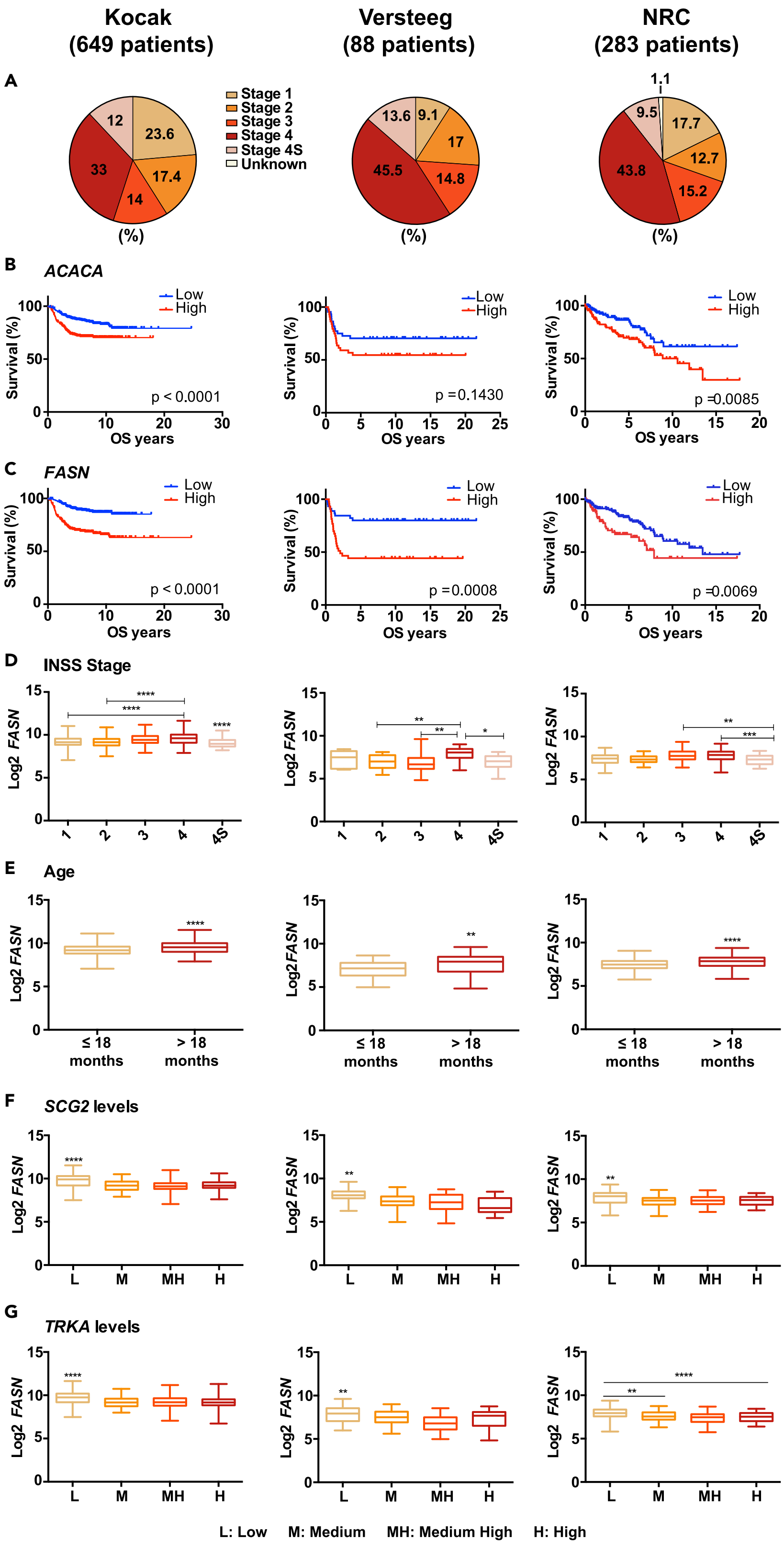

Figure 6. Expression of *FASN* and *ACACA* negatively correlates with good prognosis in neuroblastoma, related to Figure S6

(A) Distribution of patients in the different INSS stages in the three neuroblastoma datasets (Kocak, Versteeg, and NRC) analyzed in this study.

(B) Kaplan-Meier overall survival (OS) curves for each dataset based on the mRNA expression of the *ACACA* gene.

(C) Kaplan-Meier OS curves for each dataset based on the mRNA expression of the *FASN* gene.

(D–G) Boxplots of *FASN* expression related to (D) stage, (E) age, (F) quartiles of *SCG2* expression, and (G) quartiles of *TRKA* expression.

Statistics on boxplots for *MYCN* status and age: unpaired t test; statistics on boxplots for *TRKA*, *SCG2*, and stage: one-way ANOVA. *, **, ***, and **** indicate $p < 0.05$, $p < 0.01$, $p < 0.001$, and $p < 0.0001$, respectively. p values of Mantel-Cox tests are indicated for each of the OS Figures.

datasets except for *ACACA* in the Versteeg cohort (Figures S6B and S6C). In addition, the levels of both genes were higher in MNA than in NMNA patients in the Kocak dataset (Figures S6D and S6E), whereas only *FASN* expression was elevated in MNA versus NMNA patients in the two other cohorts (Figures S6D and S6E).

We next analyzed the expression of *ACACA* and *FASN* in relation to clinical parameters with prognostic value: disease stage, age at diagnosis (worse when older than 18 months), and expression levels of *TRKA* and *SCG2,* markers of both neural differentiation and good prognosis. *FASN* was expressed at higher levels in stages 3 and 4 and reduced in stage 4S patients in all three cohorts (Figure 6D), whereas *ACACA* expression showed no significant relation to disease stage in any of the datasets (Figure S6F). Patients older than 18 months at the time of diagnosis showed significantly higher levels of expression of *FASN* in all cohorts (Figure 6E), whereas significant differences between age groups were only observed in the Versteeg dataset for *ACACA* (Figure S6G). Notably, high *FASN* expression was found in patients with low *SCG2* or *TRKA* levels in all cohorts (Figures 6F and 6G), whereas elevated *ACACA* levels were only observed in tumors with low *TRKA* in the Kocak dataset (Figure S6H).

Collectively, these data suggest that although high expression of both *ACACA* and *FASN* consistently correlates to reduced survival in tumors independently of their *MYCN* status, *FASN* seems to be a better prognosis marker candidate for neuroblastoma patients.

DISCUSSION

In spite of the development of more efficient therapies, high-risk neuroblastoma remains a highly deadly disease, with a survival rate of just 50%, and the harsh treatment regimens may result in reduced quality of life (Perwein et al., 2011). It is thus urgent to develop new therapeutic approaches. Although *MYCN*-amplification strongly correlates to poor clinical outcome in neuroblastoma patients, there are no MYC-targeted therapies available in the clinic to date. Targeting processes downstream of the MYC protein, such as metabolism, and most relevant for this study, lipogenesis, has been suggested as a potent strategy to overcome difficulties of directly targeting MYC (Dang et al., 2017; Gouw et al., 2019).

Tumor cells undergo changes in metabolism to sustain cell growth and division (Cantor and Sabatini, 2012). Lipids are important not only as energy sources and structural components of membranes but also as signaling molecules (Snaebjornsson et al., 2020). The activation of *de novo* fatty acid synthesis occurs in multiple cancers (Rohrig and Schulze, 2016), and its inhibition has shown promising antitumor activity in a number of tumor types, such as ovarian (Pizer et al., 1996b; Zhou et al., 2007), breast (Pizer et al., 1996a; Thupari et al., 2001; Vazquez-Martin et al., 2007; Menendez and Lupu, 2017), prostate (Pflug et al., 2003; Pizer et al., 2001), lung (Orita et al., 2007), colon (Murata et al., 2010), and others (Menendez and Lupu, 2007; Gouw et al., 2019). However, except for one study showing reduced viability of SK-N-SH cells upon treatment with Cerulenin (Slade et al., 2003), the relevance of *de novo* fatty acid synthesis for neuroblastoma has not been previously evaluated.

Using five small molecule inhibitors targeting ACACA or FASN and siRNA targeting *FASN*, we show that inhibition of fatty acid synthesis decreases cell proliferation, reduces MYCN or c-MYC protein levels, and results in neural differentiation in neuroblastoma. We have analyzed these effects in ten neuroblastoma cell lines, two neuroblastoma PDX-derived cell cultures, one *ex vivo* model system, and three xenograft models. We further validated the clinical relevance of fatty acid synthesis for neuroblastoma by

analyzing gene expression data from primary neuroblastoma tumors from three independent patient datasets.

In contrast to inhibition of fatty acid β-oxidation, which is a vulnerability mainly in MNA neuroblastoma (Oliynyk et al., 2019), fatty acid synthesis can be successfully targeted in both MNA and NMNA neuroblastoma cell lines. We demonstrate that the consequences of fatty acid synthesis inhibition are specific for reduced lipid availability, as they can be rescued by exogenous oleate and because none of these effects are observed upon glucose or glutamine starvation.

Inhibition of fatty acid synthesis *in vivo*, both targeting ACACA with TOFA or FASN with either Orlistat or UB006, not only reduced tumor growth, but importantly led to neural differentiation, as assessed by expression of differentiation markers. These effects were striking especially when taking into account that these neuroblastoma xenograft models are very aggressive and only allow for a very short therapeutic window of 6 to 8 days after treatment initiation.

We found that MYCN levels and activity have a direct effect on fatty acid amount, as well as on glucose as the preferred carbon source for fatty acid synthesis in neuroblastoma, in agreement with our earlier results (Oliynyk et al., 2019) and studies in other MYC-driven tumors (Eberlin et al., 2014; Edmunds et al., 2014; Morrish et al., 2010; Gouw et al., 2019). We previously showed that MYCN downregulation leads to enlarged mitochondria with fewer cristae and reduced OXPHOS capacity (Oliynyk et al., 2019). Here we demonstrate that inhibition of fatty acid synthesis leads to a similar phenotype, most likely due to diminished MYC(N) levels. We also observed changes in cholesterol and ceramide levels. Although ceramides are fatty acid derivatives, and thus, reduced fatty acid content has a direct impact on their synthesis, cholesterol is produced in an independent pathway. However, cholesterol homeostasis is very dynamic, and changes in other lipids can affect cholesterol uptake, stabilization, and content (Mesmin and Maxfield, 2009). Interestingly, increased levels of p-AMPKα observed upon fatty acid synthesis inhibition may reflect a reduction of the energy production in the cells. Active p-AMPKα is known to inhibit biosynthetic processes, including cholesterol synthesis (Srivastava et al., 2012). These extended signaling processes may explain the effects of fatty acid synthesis inhibitors on cholesterol levels.

Our results suggest that the induction of differentiation is mediated by activation of ERK signaling, as this protein was phosphorylated in neuroblastoma cells subjected to fatty acid synthesis block, whereas cells were unable to differentiate when ERK activation was prevented. Although in the context of cancer biology, active ERK is usually associated to increased survival, proliferation, and malignancy (Samatar and Poulikakos, 2014), the RAF-MEK-ERK signaling pathway is also associated with neural differentiation under the control of the receptor for nerve growth factor (NGF), the neurotrophic tyrosine kinase receptor A (TRKA) (Khotskaya et al., 2017). Notably, inhibition of AKT, which lies downstream of the neurotrophin-3 receptor, TRKC (Khotskaya et al., 2017), did not affect the induction of differentiation mediated by fatty acid synthesis inhibition. Remarkably, agents that trigger differentiation of neuroblastoma or pheochromocytoma require the activation of several signaling pathways including ERK (Zogovic et al., 2015; Liu et al., 2015). ERK proteins can in turn phosphorylate more than six hundred different targets, including transcription factors, in a context-dependent manner (Unal et al., 2017), which explains their wide range of effects. Importantly, our *in silico* analysis showed that high *FASN* expression was found in patients with low *TRKA* levels in all three cohorts. Together, these data suggest that the TRKA-signaling pathway through ERK1/2 is activated by inhibition of fatty acid synthesis.

Importantly, we observed downregulation of MYCN/c-MYC protein levels upon inhibition of fatty acid synthesis. Among the pleiotropic functions of MYC, there is a well-stablished role in promoting proliferation and opposing differentiation both in normal physiological and cancer contexts (Dang, 2012; Yoshida, 2018). It has been reported that neuroblastoma cell differentiation requires a timely regulation of *MYCN* expression, with early overexpression (Guglielmi et al., 2014), followed by downregulation (Huang and Weiss, 2013). The downregulation of MYC proteins by inhibition of fatty acid synthesis could in this way be involved in the observed induction of neural differentiation.

Neuroblastoma is thought to originate from cells of embryonic origin blocked in an undifferentiated phenotype (Brodeur, 2003). Among all human cancers, it has the highest rate of spontaneous maturation and regression (Reynolds, 2000). This clinical observation raises the prospect that it may be possible to

induce the neuroblastomas that do not spontaneously differentiate into a differentiation process by therapeutic intervention. This hypothesis has led to a search for agents able to promote neuroblastoma cell maturation. Retinoids are used for the maintenance phase of treatment, but their overall benefits are unclear (Peinemann et al., 2016; Matthay et al., 2009). Finding novel agents able to induce differentiation is an important goal in the development of new treatments for neuroblastoma.

Inhibitors of both ACACA and FASN have been available for some time (Buckley et al., 2017; Wang et al., 2015); however, very few have made their way into clinical trials, despite promising effects in preclinical studies (Rohrig and Schulze, 2016). Importantly, of the inhibitors used here, only Orlistat is FDA approved and not for cancer but for the treatment of obesity (Ameer and Weintraub, 2018). All inhibitors in this study (Figure 1A), except UB006, have been employed by others to target fatty acid synthesis *in vitro* and *in vivo* in different tumor types. UB006 is a C75-derived FASN inhibitor devoid of the anorexigenic side effects associated to the parent compound (Makowski et al., 2017). It has previously not been used for treatment of tumor-carrying mice. Here we demonstrate that UB006 efficiently decreases neuroblastoma tumor growth, showing a more potent effect than TOFA and Orlistat, without affecting animal weight. Based on these promising results, we suggest that further studies on UB006 toxicology and bioavailability are justified.

Our analysis showed that high levels of both *ACACA* and *FASN* correlate with reduced survival in three neuroblastoma patient cohorts covering all five INSS stages and with similar proportion of *MYCN*-amplification. Notably high *FASN* expression is related to low levels of neural differentiation markers and to poor prognosis factors, suggesting that it may have a prognostic value in neuroblastoma.

Our results demonstrate that inhibition of fatty acid synthesis induces differentiation in neuroblastoma independently of *MYCN*-status, providing approaches for development of more specific fatty acid synthesis inhibitors that can be used in the clinic.

Limitations of the study

Our xenograft models are performed in immunocompromised mice. Future studies should analyze the influence of the environment, including the immune compartment, on the effects of fatty acid synthesis inhibition in neuroblastoma tumors. In addition, the cell lines used to perform the xenograft experiments show a very fast and aggressive growth to form tumors, limiting the therapeutic window to a range of 6 to 8 days. Both limitations could be overcome by using the transgenic *TH-MYCN* neuroblastoma mouse model, which possesses an intact immune system and spontaneously develops neuroblastoma tumors that highly resemble the characteristics of human neuroblastoma.

Resource availability

Lead contact

Further information and requests for resources and reagents should be directed to and will be fulfilled by the Lead Contact, Marie Arsenian Henriksson (marie.arsenian.henriksson@ki.se).

Materials availability

This study did not generate new unique reagents.

Data and code availability

This study did not generate datasets or code.

METHODS

All methods can be found in the accompanying Transparent Methods supplemental file.

SUPPLEMENTAL INFORMATION

Supplemental Information can be found online at https://doi.org/10.1016/j.isci.2021.102128.

ACKNOWLEDGMENTS

We are indebted to Professor C. Einvik (Artic University of Norway, Tromsö, Norway) for the BE(2)sh *MYCN* cell line, to Professor M. Schwab (German Cancer Research Center, Heidelberg, Germany) for the Tet-21/N

cells, to Professor Rolf Müller (Helmholtz Institute for Pharmaceutical Research Saarland, Saarland, Germany) for kindly providing Soraphen A, and to Dr. X. Ariza for participating in the synthesis of UB006. We are grateful to Dr. K. Hultenby and the KI TEM core facility for excellent analysis, Professor H. Hertz and K. Andersson (Royal Institute of Technology, Stockholm) for providing infrastructure for mouse experiments and for expert assistance with animal work, respectively. We acknowledge Dr. J. Milosevic (Massachusetts General Hospital, Boston, United States) for kindly providing the drawing of a child with neuroblastoma. We are thankful to Drs. C. Lökke (Arctic University of Norway, Tromsö, Norway) and L. Herrero (University of Barcelona, Barcelona, Spain) for advice, to Professor S. Lain, Dr. N. Baryawno, Dr. M. Wilhelm, and members of the Wilhelm and Arsenian-Henriksson laboratories (all KI) for fruitful discussions, and to Dr. L. Herrero for critical reading of the manuscript. This work was supported by grants to MAH from the Swedish Cancer Society (19-0510), the Swedish Childhood Cancer Fund (PR-2018-0026), the Swedish Research Council (2018-02580), the King Gustaf V Jubilee Fund, and Karolinska Institutet Foundations and to MVRP by grants from the Anna-Brita and Bo Castegren's Memorial Fund and KI Foundations. MVRP was the recipient of a postdoctoral position from the Swedish Childhood Cancer Fund and MAH of a Senior Investigator Award from the Swedish Cancer Society. IM and NB received ERASMUS funding. DS and KM were founded by the CIBER Physiopathology of Obesity and Nutrition (CB06/03/001), the Government of Catalonia (2017SGR278), and the TV3 Marató Foundation (201627-30). MTS was funded by the German Research Foundation (DFG, FOR2314).

AUTHOR CONTRIBUTIONS

MVRP and MAH designed the study. MVRP performed cell and molecular biology assays and patient data analysis. MVRP and LSA performed western blots, functional metabolic assays, mouse studies, and tumor stainings. GO carried out functional metabolic experiments, and IM and NB performed western blots and qPCRs. DS and KM synthesized and provided UB006. KA and DB contributed the PDX cells and expertise. MVRP, MAH, MTS, and AS designed, performed, and analyzed tracing and metabolomics experiments. RN provided metabolic tracers and participated in the analysis of the results. MVRP, MAH, KU, and JB designed, performed, and analyzed lipid species quantification experiments. MVRP and MAH wrote the manuscript. All authors read and commented on the text.

DECLARATION OF INTERESTS

The authors declare no competing interests.

Received: May 29, 2020
Revised: December 28, 2020
Accepted: January 27, 2021
Published: February 19, 2021

REFERENCES

Ameer, B., and Weintraub, M.A. (2018). Pediatric obesity: influence on drug dosing and therapeutics. J. Clin. Pharmacol. *58*, S94–S107.

Brodeur, G.M. (2003). Neuroblastoma: biological insights into a clinical enigma. Nat. Rev. Cancer *3*, 203–216.

Brodeur, G.M., Seeger, R.C., Schwab, M., Varmus, H.E., and Bishop, J.M. (1984). Amplification of N-myc in untreated human neuroblastomas correlates with advanced disease stage. Science *224*, 1121–1124.

Buckley, D., Duke, G., Heuer, T.S., O'farrell, M., Wagman, A.S., Mcculloch, W., and Kemble, G. (2017). Fatty acid synthase - modern tumor cell biology insights into a classical oncology target. Pharmacol. Ther. *177*, 23–31.

Cantor, J.R., and Sabatini, D.M. (2012). Cancer cell metabolism: one hallmark, many faces. Cancer Discov. *2*, 881–898.

Carroll, P.A., Diolaiti, D., Mcferrin, L., Gu, H., Djukovic, D., Du, J., Cheng, P.F., Anderson, S., Ulrich, M., Hurley, J.B., et al. (2015). Deregulated Myc requires MondoA/Mlx for metabolic reprogramming and tumorigenesis. Cancer Cell *27*, 271–285.

Carroll, P.A., Freie, B.W., Mathsyaraja, H., and Eisenman, R.N. (2018). The MYC transcription factor network: balancing metabolism, proliferation and oncogenesis. Front. Med. *12*, 412–425.

Carter, D.R., Sutton, S.K., Pajic, M., Murray, J., Sekyere, E.O., Fletcher, J., Beckers, A., De Preter, K., Speleman, F., George, R.E., et al. (2016). Glutathione biosynthesis is upregulated at the initiation of MYCN-driven neuroblastoma tumorigenesis. Mol. Oncol. *10*, 866–878.

Casinelli, G., Larosa, J., Sharma, M., Cherok, E., Banerjee, S., Branca, M., Edmunds, L., Wang, Y., Sims-Lucas, S., Churley, L., et al. (2016). N-Myc overexpression increases cisplatin resistance in neuroblastoma via deregulation of mitochondrial dynamics. Cell Death Discov. *2*, 16082.

Dang, C.V. (2012). MYC on the path to cancer. Cell *149*, 22–35.

Dang, C.V., Reddy, E.P., Shokat, K.M., and Soucek, L. (2017). Drugging the 'undruggable' cancer targets. Nat. Rev. Cancer *17*, 502–508.

Dejure, F.R., and Eilers, M. (2017). MYC and tumor metabolism: chicken and egg. EMBO J. *36*, 3409–3420.

Eberlin, L.S., Gabay, M., Fan, A.C., Gouw, A.M., Tibshirani, R.J., Felsher, D.W., and Zare, R.N. (2014). Alteration of the lipid profile in lymphomas induced by MYC overexpression. Proc. Natl. Acad. Sci. U S A *111*, 10450–10455.

Edmunds, L.R., Sharma, L., Kang, A., Lu, J., Vockley, J., Basu, S., Uppala, R., Goetzman, E.S., Beck, M.E., Scott, D., and Prochownik, E.V. (2014). c-Myc programs fatty acid metabolism and

dictates acetyl-CoA abundance and fate. J. Biol. Chem. *289*, 25382–25392.

Gaetano, C., Matsumoto, K., and Thiele, C.J. (1991). Retinoic acid negatively regulates p34cdc2 expression during human neuroblastoma differentiation. Cell Growth Differ. *2*, 487–493.

Gouw, A.M., Margulis, K., Liu, N.S., Raman, S.J., Mancuso, A., Toal, G.G., Tong, L., Mosley, A., Hsieh, A.L., Sullivan, D.K., et al. (2019). The MYC oncogene cooperates with sterol-regulated element-binding protein to regulate lipogenesis essential for neoplastic growth. Cell Metab. *30*, 556–572 e5.

Guglielmi, L., Cinnella, C., Nardella, M., Maresca, G., Valentini, A., Mercanti, D., Felsani, A., and D'Agnano, I. (2014). MYCN gene expression is required for the onset of the differentiation programme in neuroblastoma cells. Cell Death Dis. *5*, e1081.

Guillou, H., Zadravec, D., Martin, P.G., and Jacobsson, A. (2010). The key roles of elongases and desaturases in mammalian fatty acid metabolism: insights from transgenic mice. Prog. Lipid Res. *49*, 186–199.

Henriksen, J.R., Haug, B.H., Buechner, J., Tomte, E., Lokke, C., Flaegstad, T., and Einvik, C. (2011). Conditional expression of retrovirally delivered anti-MYCN shRNA as an in vitro model system to study neuronal differentiation in MYCN-amplified neuroblastoma. BMC Dev. Biol. *11*, 1.

Hsieh, J., Nakashima, K., Kuwabara, T., Mejia, E., and Gage, F.H. (2004). Histone deacetylase inhibition-mediated neuronal differentiation of multipotent adult neural progenitor cells. Proc. Natl. Acad. Sci. U S A *101*, 16659–16664.

Hsu, C.L., Chang, H.Y., Chang, J.Y., Hsu, W.M., Huang, H.C., and Juan, H.F. (2016). Unveiling MYCN regulatory networks in neuroblastoma via integrative analysis of heterogeneous genomics data. Oncotarget *7*, 36293–36310.

Huang, M., and Weiss, W.A. (2013). Neuroblastoma and MYCN. Cold Spring Harb. Perspect. Med. *3*, a014415.

Ikeda, H., Iehara, T., Tsuchida, Y., Kaneko, M., Hata, J., Naito, H., Iwafuchi, M., Ohnuma, N., Mugishima, H., Toyoda, Y., et al. (2002). Experience with international neuroblastoma staging system and pathology classification. Br. J. Cancer *86*, 1110–1116.

Khotskaya, Y.B., Holla, V.R., Farago, A.F., Mills Shaw, K.R., Meric-Bernstam, F., and Hong, D.S. (2017). Targeting TRK family proteins in cancer. Pharmacol. Ther. *173*, 58–66.

Kocak, H., Ackermann, S., Hero, B., Kahlert, Y., Oberthuer, A., Juraeva, D., Roels, F., Theissen, J., Westermann, F., Deubzer, H., et al. (2013). Hox-C9 activates the intrinsic pathway of apoptosis and is associated with spontaneous regression in neuroblastoma. Cell Death Dis. *4*, e586.

Liu, X., Wang, X., and Lu, J. (2015). Tenuifoliside A promotes neurite outgrowth in PC12 cells via the PI3K/AKT and MEK/ERK/CREB signaling pathways. Mol. Med. Rep. *12*, 7637–7642.

Maier, T., Leibundgut, M., Boehringer, D., and Ban, N. (2010). Structure and function of eukaryotic fatty acid synthases. Q. Rev. Biophys. *43*, 373–422.

Makowski, K., Mir, J.F., Mera, P., Ariza, X., Asins, G., Hegardt, F.G., Herrero, L., Garcia, J., and Serra, D. (2017). (-)-UB006: a new fatty acid synthase inhibitor and cytotoxic agent without anorexic side effects. Eur. J. Med. Chem. *131*, 207–221.

Maris, J.M. (2010). Recent advances in neuroblastoma. N. Engl. J. Med. *362*, 2202–2211.

Matthay, K.K., Reynolds, C.P., Seeger, R.C., Shimada, H., Adkins, E.S., Haas-Kogan, D., Gerbing, R.B., London, W.B., and Villablanca, J.G. (2009). Long-term results for children with high-risk neuroblastoma treated on a randomized trial of myeloablative therapy followed by 13-cis-retinoic acid: a children's oncology group study. J. Clin. Oncol. *27*, 1007–1013.

Menendez, J.A., and Lupu, R. (2007). Fatty acid synthase and the lipogenic phenotype in cancer pathogenesis. Nat. Rev. Cancer *7*, 763–777.

Menendez, J.A., and Lupu, R. (2017). Fatty acid synthase (FASN) as a therapeutic target in breast cancer. Expert Opin. Ther. Targets *21*, 1001–1016.

Mesmin, B., and Maxfield, F.R. (2009). Intracellular sterol dynamics. Biochim. Biophys. Acta *1791*, 636–645.

Molenaar, J.J., Koster, J., Zwijnenburg, D.A., Van Sluis, P., Valentijn, L.J., Van Der Ploeg, I., Hamdi, M., Van Nes, J., Westerman, B.A., Van Arkel, J., et al. (2012). Sequencing of neuroblastoma identifies chromothripsis and defects in neuritogenesis genes. Nature *483*, 589–593.

Morrish, F., Noonan, J., Perez-Olsen, C., Gafken, P.R., Fitzgibbon, M., Kelleher, J., Vangilst, M., and Hockenbery, D. (2010). Myc-dependent mitochondrial generation of acetyl-CoA contributes to fatty acid biosynthesis and histone acetylation during cell cycle entry. J. Biol. Chem. *285*, 36267–36274.

Murata, S., Yanagisawa, K., Fukunaga, K., Oda, T., Kobayashi, A., Sasaki, R., and Ohkohchi, N. (2010). Fatty acid synthase inhibitor cerulenin suppresses liver metastasis of colon cancer in mice. Cancer Sci. *101*, 1861–1865.

Nelson, D.L., Cox, M.M., and Lehninger, A.L. (2017). Lehninger Principles of Biochemistry (W.H. Freeman and Company; Macmillan Higher Education).

O'neill, E., and Kolch, W. (2004). Conferring specificity on the ubiquitous Raf/MEK signalling pathway. Br. J. Cancer *90*, 283–288.

Oliynyk, G., Ruiz-Perez, M.V., Sainero-Alcolado, L., Dzieran, J., Zirath, H., Gallart-Ayala, H., Wheelock, C.E., Johansson, H.J., Nilsson, R., Lehtio, J., and Arsenian-Henriksson, M. (2019). MYCN-enhanced oxidative and glycolytic metabolism Reveals vulnerabilities for targeting neuroblastoma. iScience *21*, 188–204.

Orita, H., Coulter, J., Lemmon, C., Tully, E., Vadlamudi, A., Medghalchi, S.M., Kuhajda, F.P., and Gabrielson, E. (2007). Selective inhibition of fatty acid synthase for lung cancer treatment. Clin. Cancer Res. *13*, 7139–7145.

Peinemann, F., Van Dalen, E.C., and Berthold, F. (2016). Retinoic acid for high-risk neuroblastoma patients after autologous stem cell transplantation - cochrane Review. Klin. Padiatr. *228*, 124–129.

Persson, C.U., Von Stedingk, K., Bexell, D., Merselius, M., Braekeveldt, N., Gisselsson, D., Arsenian-Henriksson, M., Pahlman, S., and Wigerup, C. (2017). Neuroblastoma patient-derived xenograft cells cultured in stem-cell promoting medium retain tumorigenic and metastatic capacities but differentiate in serum. Sci. Rep. *7*, 10274.

Perwein, T., Lackner, H., Sovinz, P., Benesch, M., Schmidt, S., Schwinger, W., and Urban, C. (2011). Survival and late effects in children with stage 4 neuroblastoma. Pediatr. Blood Cancer *57*, 629–635.

Pflug, B.R., Pecher, S.M., Brink, A.W., Nelson, J.B., and Foster, B.A. (2003). Increased fatty acid synthase expression and activity during progression of prostate cancer in the TRAMP model. Prostate *57*, 245–254.

Pizer, E.S., Jackisch, C., Wood, F.D., Pasternack, G.R., Davidson, N.E., and Kuhajda, F.P. (1996a). Inhibition of fatty acid synthesis induces programmed cell death in human breast cancer cells. Cancer Res. *56*, 2745–2747.

Pizer, E.S., Pflug, B.R., Bova, G.S., Han, W.F., Udan, M.S., and Nelson, J.B. (2001). Increased fatty acid synthase as a therapeutic target in androgen-independent prostate cancer progression. Prostate *47*, 102–110.

Pizer, E.S., Wood, F.D., Heine, H.S., Romantsev, F.E., Pasternack, G.R., and Kuhajda, F.P. (1996b). Inhibition of fatty acid synthesis delays disease progression in a xenograft model of ovarian cancer. Cancer Res. *56*, 1189–1193.

Pugh, T.J., Morozova, O., Attiyeh, E.F., Asgharzadeh, S., Wei, J.S., Auclair, D., Carter, S.L., Cibulskis, K., Hanna, M., Kiezun, A., et al. (2013). The genetic landscape of high-risk neuroblastoma. Nat. Genet. *45*, 279–284.

Rajbhandari, P., Lopez, G., Capdevila, C., Salvatori, B., Yu, J., Rodriguez-Barrueco, R., Martinez, D., Yarmarkovich, M., Weichert-Leahey, N., Abraham, B.J., et al. (2018). Cross-cohort analysis identifies a TEAD4-MYCN positive feedback loop as the core regulatory element of high-risk neuroblastoma. Cancer Discov. *8*, 582–599.

Ren, P., Yue, M., Xiao, D., Xiu, R., Gan, L., Liu, H., and Qing, G. (2015). ATF4 and N-Myc coordinate glutamine metabolism in MYCN-amplified neuroblastoma cells through ASCT2 activation. J. Pathol. *235*, 90–100.

Reynolds, C.P. (2000). Differentiating agents in pediatric malignancies: retinoids in neuroblastoma. Curr. Oncol. Rep. *2*, 511–518.

Ribeiro, D., Klarqvist, M.D., Westermark, U.K., Oliynyk, G., Dzieran, J., Kock, A., Savatier Banares, C., Hertwig, F., Johnsen, J.I., Fischer, M., Kogner, P., Loven, J., and Arsenian-Henriksson, M. (2016). Regulation of Nuclear Hormone Receptors by MYCN-Driven miRNAs Impacts Neural Differentiation and Survival in Neuroblastoma Patients. Cell Rep. *16*, 979–993.

Rohrig, F., and Schulze, A. (2016). The multifaceted roles of fatty acid synthesis in cancer. Nat. Rev. Cancer *16*, 732–749.

Ruiz-Pérez, M.V., Albihn, A., and Arsenian-Henriksson, M. (2015). Myc. In Encyclopedia of Cancer, M. Schwab, ed. (Springer-Verlag), pp. 117–123.

Ruiz-Perez, M.V., Henley, A.B., and Arsenian-Henriksson, M. (2017). The MYCN protein in health and disease. Genes (Basel) *8*, 113.

Samatar, A.A., and Poulikakos, P.I. (2014). Targeting RAS-ERK signalling in cancer: promises and challenges. Nat. Rev. Drug Discov. *13*, 928–942.

Santos, C.R., and Schulze, A. (2012). Lipid metabolism in cancer. FEBS J. *279*, 2610–2623.

Slade, R.F., Hunt, D.A., Pochet, M.M., Venema, V.J., and Hennigar, R.A. (2003). Characterization and inhibition of fatty acid synthase in pediatric tumor cell lines. Anticancer Res. *23*, 1235–1243.

Snaebjornsson, M.T., Janaki-Raman, S., and Schulze, A. (2020). Greasing the wheels of the cancer machine: the role of lipid metabolism in cancer. Cell Metab. *31*, 62–76.

Srivastava, R.A., Pinkosky, S.L., Filippov, S., Hanselman, J.C., Cramer, C.T., and Newton, R.S. (2012). AMP-activated protein kinase: an emerging drug target to regulate imbalances in lipid and carbohydrate metabolism to treat cardio-metabolic diseases. J. Lipid Res. *53*, 2490–2514.

Thupari, J.N., Pinn, M.L., and Kuhajda, F.P. (2001). Fatty acid synthase inhibition in human breast cancer cells leads to malonyl-CoA-induced inhibition of fatty acid oxidation and cytotoxicity. Biochem. Biophys. Res. Commun. *285*, 217–223.

Unal, E.B., Uhlitz, F., and Bluthgen, N. (2017). A compendium of ERK targets. FEBS Lett. *591*, 2607–2615.

Vazquez-Martin, A., Ropero, S., Brunet, J., Colomer, R., and Menendez, J.A. (2007). Inhibition of Fatty Acid Synthase (FASN) synergistically enhances the efficacy of 5-fluorouracil in breast carcinoma cells. Oncol. Rep. *18*, 973–980.

Wang, C., Ma, J., Zhang, N., Yang, Q., Jin, Y., and Wang, Y. (2015). The acetyl-CoA carboxylase enzyme: a target for cancer therapy? Expert Rev. Anticancer Ther. *15*, 667–676.

Whittle, S.B., Smith, V., Doherty, E., Zhao, S., Mccarty, S., and Zage, P.E. (2017). Overview and recent advances in the treatment of neuroblastoma. Expert Rev. Anticancer Ther. *17*, 369–386.

Yoshida, G.J. (2018). Emerging roles of Myc in stem cell biology and novel tumor therapies (vol 37, pg 173, 2018). J. Exp. Clin. Cancer Res. *37*, 173, https://doi.org/10.1186/s13046-018-0835-y.

Zhou, W., Han, W.F., Landree, L.E., Thupari, J.N., Pinn, M.L., Bililign, T., Kim, E.K., Vadlamudi, A., Medghalchi, S.M., El Meskini, R., et al. (2007). Fatty acid synthase inhibition activates AMP-activated protein kinase in SKOV3 human ovarian cancer cells. Cancer Res. *67*, 2964–2971.

Zirath, H., Frenzel, A., Oliynyk, G., Segerstrom, L., Westermark, U.K., Larsson, K., Munksgaard Persson, M., Hultenby, K., Lehtio, J., Einvik, C., et al. (2013). MYC inhibition induces metabolic changes leading to accumulation of lipid droplets in tumor cells. Proc. Natl. Acad. Sci. U S A *110*, 10258–10263.

Zogovic, N., Tovilovic-Kovacevic, G., Misirkic-Marjanovic, M., Vucicevic, L., Janjetovic, K., Harhaji-Trajkovic, L., and Trajkovic, V. (2015). Coordinated activation of AMP-activated protein kinase, extracellular signal-regulated kinase, and autophagy regulates phorbol myristate acetate-induced differentiation of SH-SY5Y neuroblastoma cells. J. Neurochem. *133*, 223–232.

iScience, Volume *24*

Supplemental Information

Inhibition of fatty acid synthesis induces differentiation and reduces tumor burden in childhood neuroblastoma

María Victoria Ruiz-Pérez, Lourdes Sainero-Alcolado, Ganna Oliynyk, Isabell Matuschek, Nicola Balboni, S.J. Kumari A. Ubhayasekera, Marteinn Thor Snaebjornsson, Kamil Makowski, Kristina Aaltonen, Daniel Bexell, Dolors Serra, Roland Nilsson, Jonas Bergquist, Almut Schulze, and Marie Arsenian-Henriksson

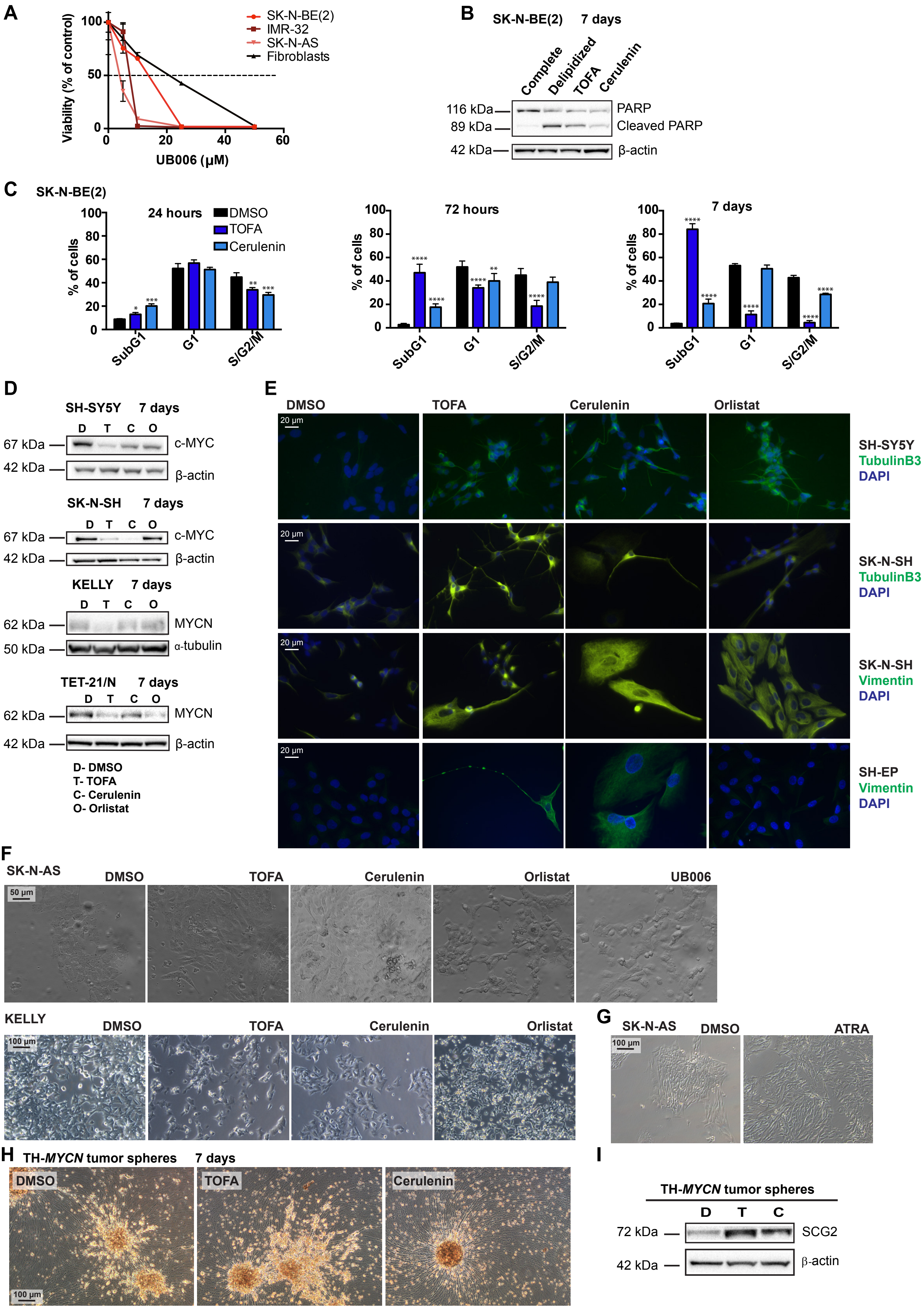

A
Viability (% of control)
UB006 (µM)
SK-N-BE(2)
IMR-32
SK-N-AS
Fibroblasts
B
SK-N-BE(2) 7 days
Complete
Delipidized
TOFA
Cerulenin
116 kDa
89 kDa
42 kDa
PARP
Cleaved PARP
β-actin
C
SK-N-BE(2)
24 hours
72 hours
7 days
DMSO
TOFA
Cerulenin
% of cells
SubG1
G1
S/G2/M
D
SH-SY5Y 7 days
D T C O
67 kDa
42 kDa
c-MYC
β-actin
SK-N-SH 7 days
KELLY 7 days
62 kDa
50 kDa
MYCN
α-tubulin
TET-21/N 7 days
D- DMSO
T- TOFA
C- Cerulenin
O- Orlistat
E
DMSO
TOFA
Cerulenin
Orlistat
20 µm
SH-SY5Y
TubulinB3
DAPI
SK-N-SH
TubulinB3
DAPI
SK-N-SH
Vimentin
DAPI
SH-EP
Vimentin
DAPI
F
SK-N-AS
DMSO
TOFA
Cerulenin
Orlistat
UB006
50 µm
KELLY
100 µm
G
SK-N-AS
DMSO
ATRA
H
TH-MYCN tumor spheres 7 days
I
TH-MYCN tumor spheres
D T C
72 kDa
SCG2
42 kDa
β-actin

Figure S1. Related to Figure 1. Inhibition of *de novo* fatty acid synthesis reduces cell growth and MYC expression, and induces differentiation in neuroblastoma cells

A) Viability, as determined by WST-1 assay, of SK-N-BE(2), IMR-32, SK-N-AS, or human primary fibroblasts treated for 72 h with the indicated concentrations of UB006. Data is presented as mean $\pm$ SD of three independent experiments in % of control. **B)** Western blot analysis of total and cleaved PARP protein in SK-N-BE(2) cells upon the indicated treatments. β-actin was used as loading control. **C)** Cell cycle analysis by propidium iodide-FACS in SK-N-BE(2) cells treated with vehicle or the indicated inhibitors at the specified time points. Data is presented as mean $\pm$ SD of three independent experiments; statistical analysis: t test of each inhibitor compared to vehicle with *, **, ***, **** indicating $p<0.05$, $p<0.01$, $p<0.001$ and $p<0.0001$, respectively. **D)** Western blot analysis of c-MYC or MYCN, as indicated, in the specified cell lines upon fatty acid synthesis inhibition. β-actin or α-tubulin were used as loading controls as indicated. **E)** Immunofluorescence staining of the indicated cell lines treated for seven days as indicated. Blue: DAPI; green: tubulin B3 or vimentin, as indicated. Scale bar indicates 20 µm. **F)** Bright field pictures of KELLY (scale bar indicates 100 µm) or SK-N-AS (scale bar indicates 50 µm) cells treated for seven days as described. **G)** Bright field pictures of SK-N-AS cells treated with vehicle or ATRA for seven days. Scale bar indicates 50 µm. **H)** Bright field pictures of TH-*MYCN* tumor spheres in differentiation conditions, treated with DMSO, TOFA, or Cerulenin for seven days. Scale bar indicates 100 µm. **I)** Western blot analysis of SCG2 in TH-*MYCN* tumor spheres in differentiation conditions, treated with DMSO, TOFA, or Cerulenin for seven days. β-actin was used as loading control. Western blot and microscopic images are representative of at least three independent experiments.

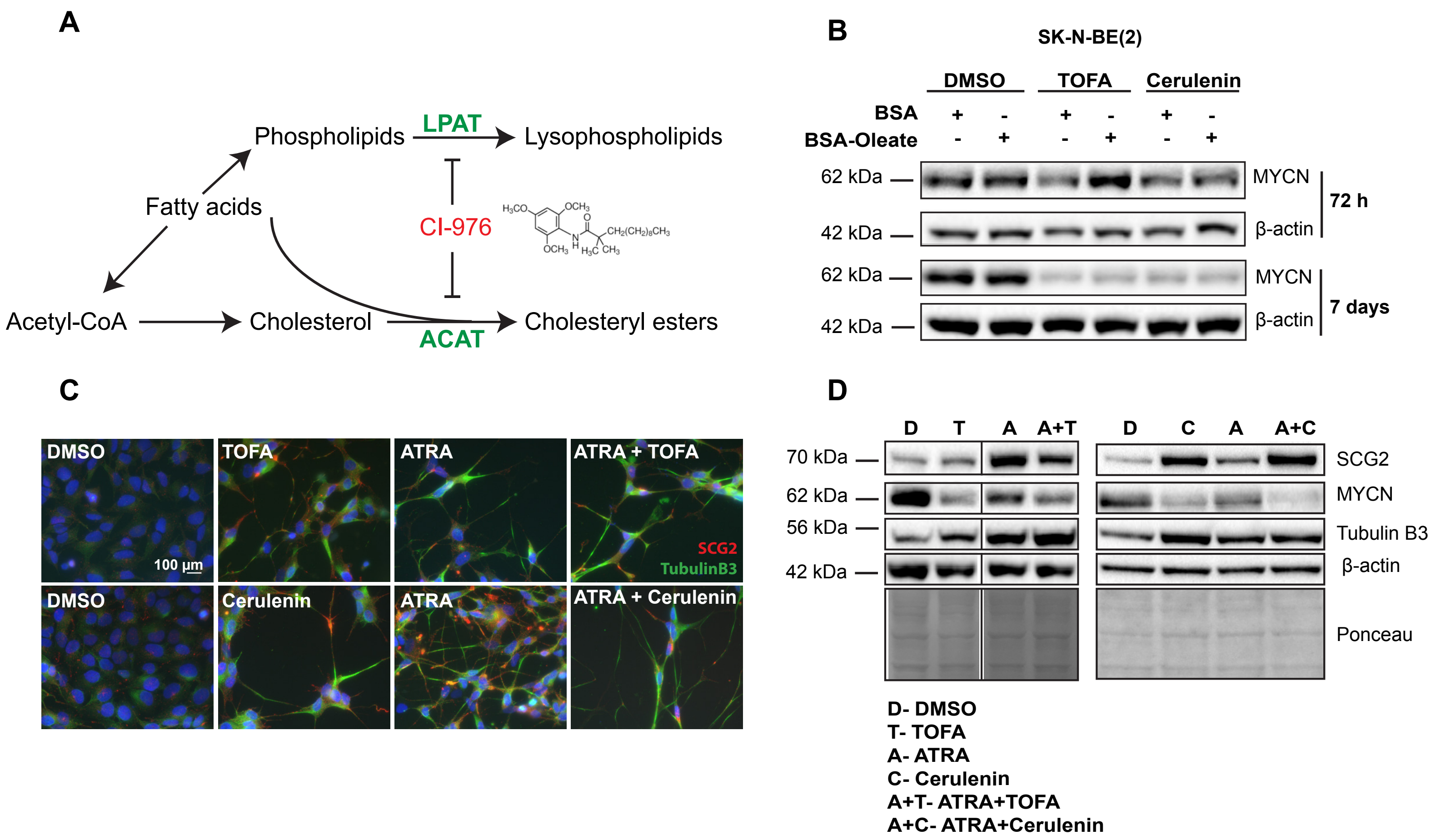
A
Phospholipids
LPAT
Lysophospholipids
Fatty acids
CI-976
Acetyl-CoA
Cholesterol
ACAT
Cholesteryl esters
B
SK-N-BE(2)
DMSO
TOFA
Cerulenin
BSA
BSA-Oleate
62 kDa
42 kDa
MYCN
β-actin
72 h
7 days
C
DMSO
TOFA
ATRA
ATRA + TOFA
100 µm
SCG2
TubulinB3
Cerulenin
ATRA + Cerulenin
D
D T A A+T
D C A A+C
70 kDa
62 kDa
56 kDa
42 kDa
SCG2
MYCN
Tubulin B3
β-actin
Ponceau
D- DMSO
T- TOFA
A- ATRA
C- Cerulenin
A+T- ATRA+TOFA
A+C- ATRA+Cerulenin

Figure S2. Related to Figure 2. Induction of differentiation is not a general response to nutrient starvation, but a specific consequence of diminished fatty acid synthesis and lipid withdrawal

A) Enzymatic reactions inhibited by CI-976. LPAT: lysophospholipid acyltransferase; ACAT: sterol O-acyltransferase. **B)** Western blot analysis of MYCN protein in SK-N-BE(2) cells treated with vehicle, TOFA, or Cerulenin ± BSA/BSA-bound oleate for three or seven days. β-actin was used as loading control. The image is representative of three independent experiments. **C)** Immunofluorescence staining of SK-N-BE(2) cells, treated for seven days with vehicle or the indicated compounds. Blue: DAPI; green: tubulin B3; red: SCG2. Scale bar indicates 100 µm. **D)** Western blot analysis of SCG2, MYCN and Tubulin B3 proteins in SK-N-BE(2) cells treated with vehicle, TOFA, or Cerulenin ± ATRA for seven days. β-actin was used as loading control. The image is representative of three independent experiments.

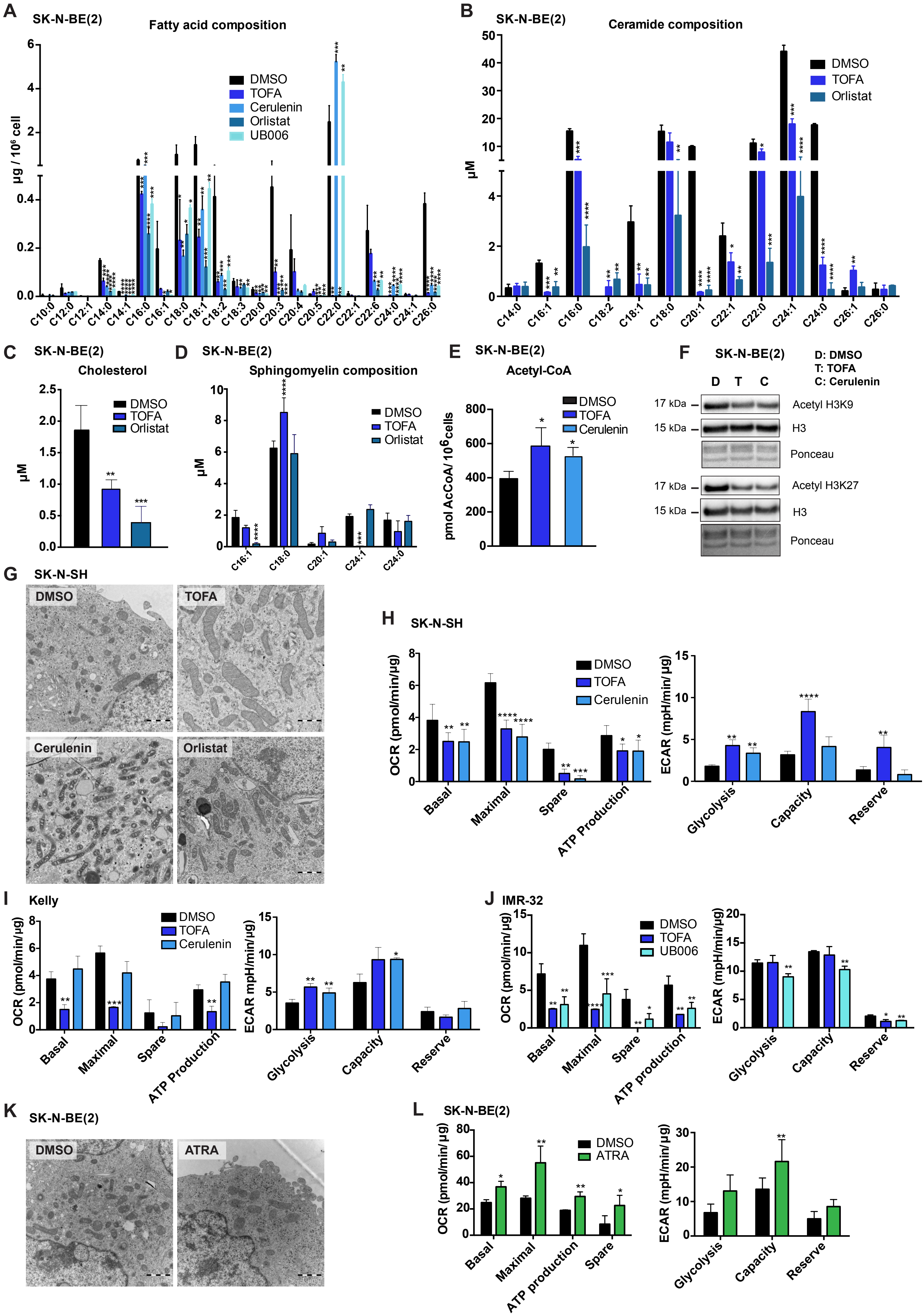

A
SK-N-BE(2)
Fatty acid composition
μg / 10⁶ cell
DMSO
TOFA
Cerulenin
Orlistat
UB006
C10:0
C12:0
C12:1
C14:0
C14:1
C16:0
C16:1
C18:0
C18:1
C18:2
C18:3
C20:0
C20:3
C20:4
C20:5
C22:0
C22:1
C22:6
C24:0
C24:1
C26:0
B
SK-N-BE(2)
Ceramide composition
μM
DMSO
TOFA
Orlistat
C14:0
C16:1
C16:0
C18:2
C18:1
C18:0
C20:1
C22:1
C22:0
C24:1
C24:0
C26:1
C26:0
C
SK-N-BE(2)
Cholesterol
μM
DMSO
TOFA
Orlistat
D
SK-N-BE(2)
Sphingomyelin composition
μM
DMSO
TOFA
Orlistat
C16:1
C18:0
C20:1
C24:1
C24:0
E
SK-N-BE(2)
Acetyl-CoA
pmol AcCoA/ 10⁶cells
DMSO
TOFA
Cerulenin
F
SK-N-BE(2)
D: DMSO
T: TOFA
C: Cerulenin
D T C
17 kDa
Acetyl H3K9
15 kDa
H3
Ponceau
17 kDa
Acetyl H3K27
15 kDa
H3
Ponceau
G
SK-N-SH
DMSO
TOFA
Cerulenin
Orlistat
H
SK-N-SH
OCR (pmol/min/μg)
DMSO
TOFA
Cerulenin
Basal
Maximal
Spare
ATP Production
ECAR (mpH/min/ μg)
Glycolysis
Capacity
Reserve
I
Kelly
OCR (pmol/min/μg)
DMSO
TOFA
Cerulenin
Basal
Maximal
Spare
ATP Production
ECAR mpH/min/μg)
Glycolysis
Capacity
Reserve
J
IMR-32
OCR (pmol/min/μg)
DMSO
TOFA
UB006
Basal
Maximal
Spare
ATP production
ECAR (mpH/min/μg)
Glycolysis
Capacity
Reserve
K
SK-N-BE(2)
DMSO
ATRA
L
SK-N-BE(2)
OCR (pmol/min/μg)
DMSO
ATRA
Basal
Maximal
ATP production
Spare
ECAR (mpH/min/μg)
Glycolysis
Capacity
Reserve

Figure S3. Related to Figure 3. Inhibition of fatty acid synthesis has a broad impact on cellular lipid composition and affects mitochondrial morphology and function

A-D) Abundance of the indicated lipid species in SK-N-BE(2) cells treated for seven days with vehicle or the indicated fatty acid synthesis inhibitors, determined by LC. **A)** Fatty acids. **B)** Ceramides. **C)** Cholesterol. **D)** Sphingomyelins. **E)** Total Acetyl-CoA concentration in SK-N-BE(2) cells treated for seven days with vehicle or the indicated fatty acid synthesis inhibitors. **F)** Western blot analysis of acetyl H3K9, Acetyl H3K27 and total histone 3 (H3) in SK-N-BE(2) cells treated with vehicle, TOFA, or Cerulenin for 24 hours. Ponceau staining was used as loading control. The image is representative of three independent experiments. **G)** Transmission electron microscopy images of representative mitochondria in SK-N-SH cells treated for seven days with vehicle or the indicated fatty acid synthesis inhibitors. Scale bars indicate 1 µm. **H)** Quantification of OCR (left panel) and ECAR (right panel) in SK-N-SH cells treated for 72 h with vehicle or the indicated fatty acid synthesis inhibitors. Data is presented as mean $\pm$ SD of two independent experiments. **I)** and **J)** Quantification of OCR (left panels) and ECAR (right panels) in Kelly and IMR-32 cells, respectively, treated for 72 h with vehicle or the indicated fatty acid synthesis inhibitors. **K)** Transmission electron microscopy images of representative mitochondria in SK-N-BE(2) cells treated for seven days with vehicle or ATRA. Scale bars indicate 1 µm. **L)** Quantification of OCR (left panel) and ECAR (right panel) in SK-N-BE(2) cells treated for 72 h with vehicle or ATRA.

All data, except where indicated, is presented as mean $\pm$ SD of at least three independent experiments; statistical analysis t-test with *, **, ***, **** indicating $p<0.05$, $p<0.01$, $p<0.001$ and $p<0.0001$, respectively. Microscopic images are representative of at least three independent experiments.

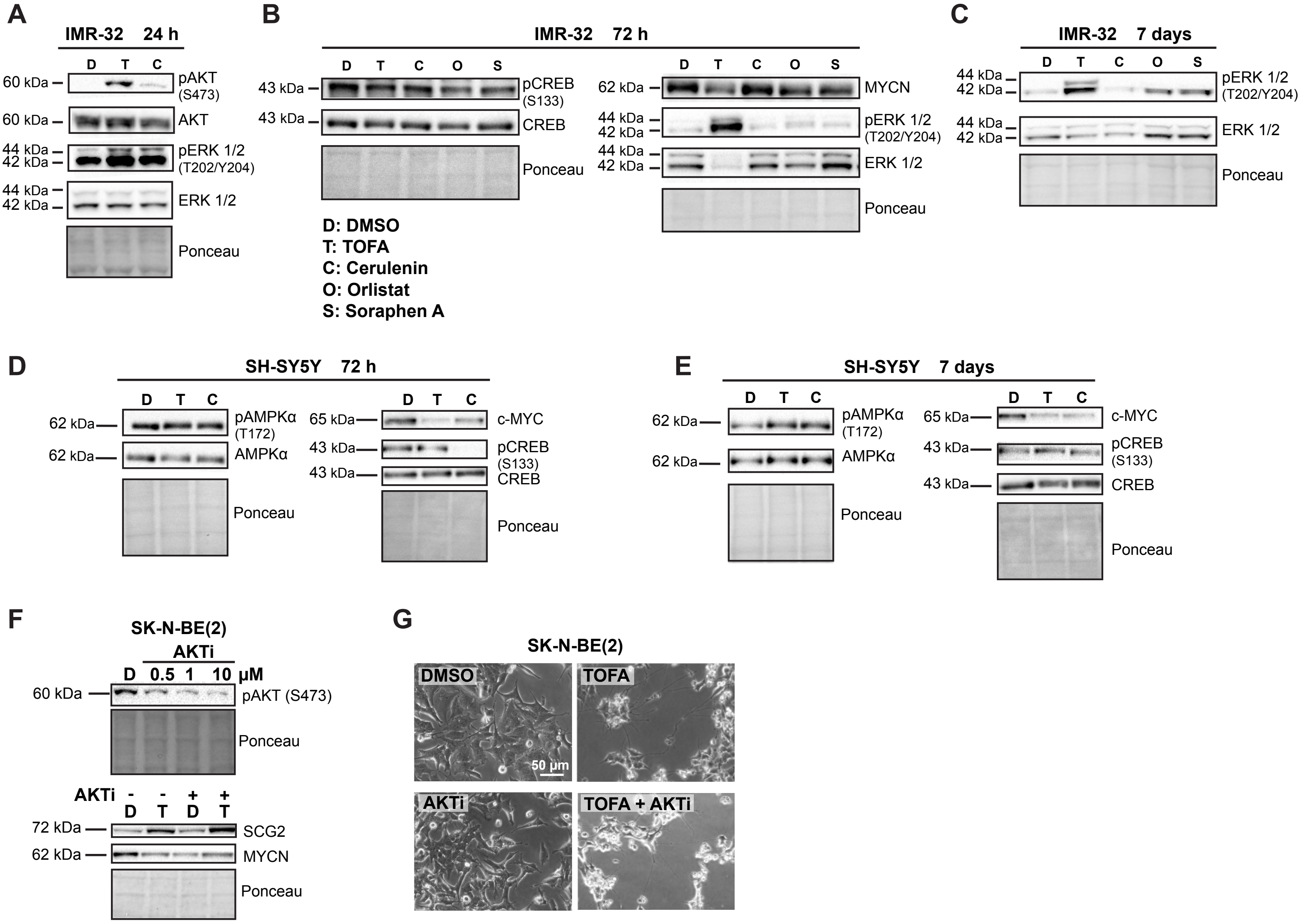
A
IMR-32 24 h
D T C
60 kDa pAKT (S473)
60 kDa AKT
44 kDa 42 kDa pERK 1/2 (T202/Y204)
44 kDa 42 kDa ERK 1/2
Ponceau
B
IMR-32 72 h
D T C O S
43 kDa pCREB (S133)
43 kDa CREB
Ponceau
D: DMSO
T: TOFA
C: Cerulenin
O: Orlistat
S: Soraphen A
62 kDa MYCN
44 kDa 42 kDa pERK 1/2 (T202/Y204)
44 kDa 42 kDa ERK 1/2
Ponceau
C
IMR-32 7 days
D T C O S
44 kDa 42 kDa pERK 1/2 (T202/Y204)
44 kDa 42 kDa ERK 1/2
Ponceau
D
SH-SY5Y 72 h
D T C
62 kDa pAMPKα (T172)
62 kDa AMPKα
Ponceau
65 kDa c-MYC
43 kDa pCREB (S133)
43 kDa CREB
Ponceau
E
SH-SY5Y 7 days
D T C
62 kDa pAMPKα (T172)
62 kDa AMPKα
Ponceau
65 kDa c-MYC
43 kDa pCREB (S133)
43 kDa CREB
Ponceau
F
SK-N-BE(2)
AKTi
D 0.5 1 10 µM
60 kDa pAKT (S473)
Ponceau
AKTi - - + +
D T D T
72 kDa SCG2
62 kDa MYCN
Ponceau
G
SK-N-BE(2)
DMSO
TOFA
50 µm
AKTi
TOFA + AKTi

Figure S4. Related to Figure 4. Induction of differentiation upon fatty acid synthesis inhibition is dependent on ERK signaling

A-C) Western blot analysis of the indicated proteins, in their total forms or phosphorylated in the indicated residues, in IMR-32 cells treated for 24 h (**A**), 72 h (**B**) or seven days (**C**) as indicated. **D-E)** Western blot analysis of the indicated proteins, in their total forms or phosphorylated in the indicated residues, in SH-SY5Y cells treated for 72 h (**D**) or seven days (**E**) with vehicle or the indicated inhibitors. **F)** Western blot analysis of phosphorylated AKT in SK-N-BE(2) cells treated for 24 h with DMSO or AKT inhibitor (AKTi; upper blot) and SCG2 and MYCN proteins in SK-N-BE(2) cells treated for seven days with DMSO (D) and TOFA (T) ± AKT inhibitor (Ai) (lower blot). **G)** Bright field pictures of SK-N-BE(2) cells treated with DMSO or TOFA ± AKT inhibitor (AKTi). Scale bar indicates 50 µm.

Ponceau staining was used as loading control in the Western blots in A-F. Western blot and microscopic images are representative of at least three independent experiments.

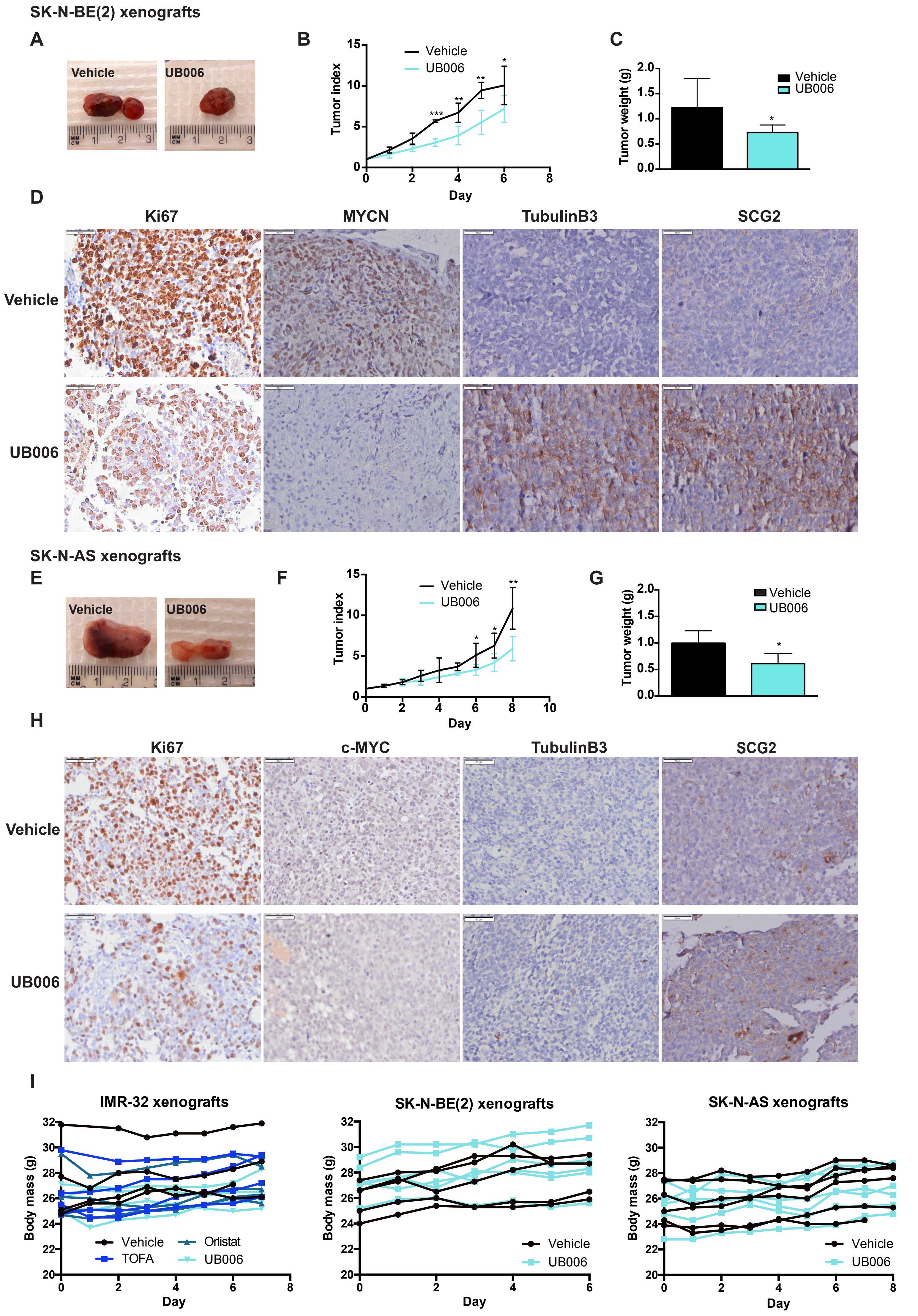

SK-N-BE(2) xenografts
A
Vehicle
UB006
B
Tumor index
Day
Vehicle
UB006
C
Tumor weight (g)
Vehicle
UB006
D
Ki67
MYCN
TubulinB3
SCG2
Vehicle
UB006
SK-N-AS xenografts
E
Vehicle
UB006
F
Tumor index
Day
Vehicle
UB006
G
Tumor weight (g)
Vehicle
UB006
H
Ki67
c-MYC
TubulinB3
SCG2
Vehicle
UB006
I
IMR-32 xenografts
Body mass (g)
Day
Vehicle
Orlistat
TOFA
UB006
SK-N-BE(2) xenografts
Body mass (g)
Day
Vehicle
UB006
SK-N-AS xenografts
Body mass (g)
Day
Vehicle
UB006

Figure S5. Related to Figure 5. Inhibition of fatty acid synthesis reduces neuroblastoma tumor growth and increases differentiation *in vivo*

A) Representative pictures of the SK-N-BE(2) xenograft tumors at the experimental endpoint treated with vehicle or UB006 as indicated. **B)** Tumor index (volume each day / initial volume) of a xenograft model with SK-N-BE(2) cells. NMRI nude mice were treated daily with vehicle (10 % β-cyclodextrin) or 20 mg/kg UB006. n = 5 per group. **C)** Tumor weight at the experiment endpoint. Data is presented as mean ± SD. Statistical analysis: t test with *, **, *** indicating p<0.05, p<0.01 and p<0.001 respectively. **D)** Microscopic images of IHC staining of SK-N-BE(2) xenograft tumors labeled with anti-Ki67, anti-MYCN, anti-Tubulin B3, and anti-SCG2 antibodies. Scales bars indicate 50 µm. **E)** Representative pictures of the SK-N-AS xenograft tumors at the experimental endpoint treated with vehicle or UB006 as indicated. **F)** Tumor index of a xenograft model with SK-N-AS cells. NMRI nude mice were treated daily with vehicle (10 % β-cyclodextrin) or 20 mg/kg UB006. n = 6 per group. **G)** Tumor weight at the experiment endpoint. Data is presented as mean ± SD. Statistical analysis: t test with *, ** indicating p<0.01 and p<0.001, respectively. **H)** Microscopic images of IHC staining of SK-N-AS xenograft tumors labeled with anti-Ki67, anti-c-MYC, anti-Tubulin B3, and anti-SCG2 antibodies. Scales bars indicate 50 µm. **I)** Body weight of each mouse throughout each of the xenograft experiments as indicated.

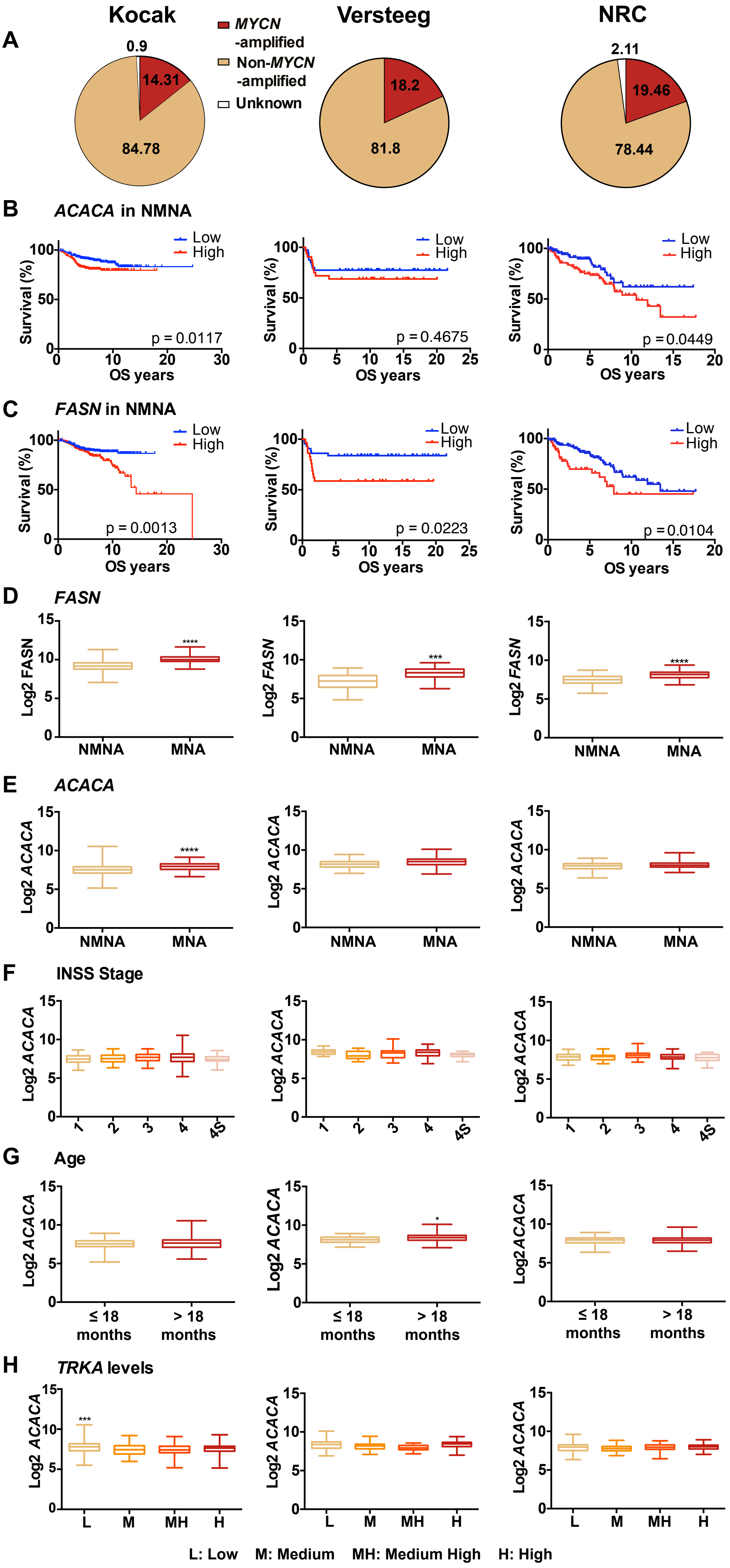

A
Kocak
Versteeg
NRC
MYCN -amplified
Non-MYCN -amplified
Unknown
0.9
14.31
84.78
18.2
81.8
2.11
19.46
78.44
B
ACACA in NMNA
C
FASN in NMNA
Low
High
Survival (%)
OS years
p = 0.0117
p = 0.4675
p = 0.0449
p = 0.0013
p = 0.0223
p = 0.0104
D
FASN
Log2 FASN
NMNA
MNA
E
ACACA
Log2 ACACA
F
INSS Stage
1
2
3
4
4S
G
Age
≤ 18 months
> 18 months
H
TRKA levels
L
M
MH
H
L: Low M: Medium MH: Medium High H: High

Figure S6. Related to Figure 6. Expression of *FASN* and *ACACA* negatively correlates with good prognosis in neuroblastoma

A) Distribution of patients into *MYCN*-amplified or non-amplified categories in the three neuroblastoma datasets (Kocak, Versteeg, and NRC) analyzed in this study. **B)** Kaplan-Meier overall survival (OS) curve of the non-*MYCN*-amplified (NMNA) patients from the three cohorts based on the mRNA expression of *ACACA*. **C)** Kaplan-Meier overall survival (OS) curve of the non-*MYCN*-amplified (NMNA) patients from the three cohorts based on the mRNA expression of *FASN*. **D)** Boxplots of *FASN* expression related to *MYCN*-amplification status. **E)** Boxplots of *ACACA* expression related to *MYCN*-amplification status. **F)** Boxplots of *ACACA* expression related to INSS stage. **G)** Boxplots of *ACACA* expression related to age at the time of diagnosis. **H)** Boxplots of *ACACA* expression related to quartiles of *TRKA* expression.

p values of Mantel-Cox tests are indicated for each Kaplan-Meier Figure in A and B. Statistics on boxplots for *MYCN* status and age, unpaired t test; statistics on boxplots for stage and *TRKA*, one-way ANOVA. * indicating $p<0.05$; ** indicating $p<0.01$, *** indicating $p<0.001$ and **** indicating $p<0.0001$.

Supplemental Table 1. Related to Figure 1. Summary of the cell lines used in this study. General information of cell lines from (Thiele, 1998, ATCC).

**Cells**	***MYCN* status**	**Derived from**
SK-N-BE(2)	Amplified	NB bone marrow metastasis from a two years old male
SK-N-BE(2)sh*MYCN*	Amplified	SK-N-BE(2) with a doxycycline-inducible *shMYCN* RNA (Henriksen et al., 2011)
IMR-32	Amplified	Primary NB from a thirteen months old male
KCN-69n	Amplified	NB bone marrow metastasis from an eleven months old male
KELLY<br>Also called NB19	Amplified	NB bone marrow metastasis from a one year old female
LU-NB-2	Amplified	NB cerebral metastasis from a two years old child (Braekeveldt et al., 2015)
LU-NB-3	Amplified	NB primary adrenal-gland tumor from a two years old child (Braekeveldt et al., 2015)
SK-N-AS	Wild type	NB bone marrow metastasis from a six years old female
SK-N-SH	Wild type	NB bone marrow metastasis from a four years old female
SH-SY5Y	Wild type	SK-N-SH subclone
SH-EP	Wild type	SK-N-SH subclone
TET-21/N	Overexpression	SH-EP overexpressing a doxycycline-repressible *MYCN* gene (Lutz et al., 1996)
TH-*MYCN* tumor spheres	Overexpression	Primary tumors from TH-*MYCN* mice (Ribeiro et al., 2016, Weiss et al., 1997)
Human primary fibroblasts	Wild type	Adult human skin

TRANSPARENT METHODS

EXPERIMENTAL MODELS AND SUBJECT DETAILS

Animal studies

Five-week-old female NMRI-*Foxn1*^{nu} mice purchased from Taconic Biosciences were housed, treated, and analyzed in accordance with the ethical permit number N71/15 approved by the Swedish ethical committee "Stockholms Norra Djurförsöksetiska Nämnd". Mice were housed in specific pathogen free conditions where light, temperature (+21°C), and relative humidity (50-60%) were controlled. Food and water were available *ad libitum*. For xenograft experiments, 10 x 10^6 SK-N-BE(2), IMR-32, or SK-N-AS cells were injected subcutaneously into the right flanks. IMR-32 cells were injected in a 1/3 Matrigel (Sigma-Aldrich) dilution. The other cell lines were prepared in culture medium without supplements. Tumor growth was monitored daily by caliper measurement of three dimensions. Tumor take point was defined as a tumor volume equal to 0.1 cm^3. The animals were then randomized into the treatment groups and received daily *i.p.* injections with either vehicle (10 % 2-(hydroxypropyl)-β-cyclodextrin in H_2O) or 30 mg/kg TOFA, 100 mg/kg Orlistat, or 20 mg/kg UB006. The mice were euthanized after seven to eight days of treatment or after tumors reached 1 cm^3. The procedures for performing all animal experiments were in accordance with the principles and guidelines of Karolinska Institutet and the Swedish law.

TH-*MYCN* tumor spheres

For generation of TH-*MYCN*-derived tumor spheres, tumors from homozygous TH-*MYCN* mice were cut into small pieces and mechanically dissociated. The resulting cell suspension was washed with PBS twice and filtered through a 70 µm nylon membrane. To remove blood cells, the cell suspensions were treated with red blood lysis buffer (Sigma-Aldrich), followed a wash with PBS. For differentiation, the cells were plated at a density of 5 x 10^5 cells/cm^2 on culture dishes coated with poly-D-lysine and laminin and incubated in DMEM/F12 medium (Sigma-Aldrich) supplemented with 2 % B-27 (Fisher Scientific) and 5 ng/ml ciliary neurotrophic factor (R&D Systems) (Ribeiro et al., 2016).

Cell culture

The human NB cell lines SK-N-BE(2), KCN-n69, KELLY, SK-N-AS, SK-N-SH, SH-SY5Y, and SH-EP were grown in MEME (Sigma-Aldrich):Nutrient Mixture F-12 (Gibco) (1:1) medium containing 10 % fetal bovine serum (Hyclone) 1 % non-essential amino acids (NEAA, Sigma-Aldrich), 1 % glutamine (Hyclone) and 1 % penicillin-streptomycin (Hyclone). The SK-N-BE(2)sh*MYCN* cells, expressing a doxycycline inducible *MYCN*-shRNA, and the Tet-21/N cells, expressing a doxycycline-repressible *MYCN* gene, were cultured in the same medium supplemented with 10 % tetracycline-approved FBS (Clontech). Human neuroblastoma IMR-32 cells were grown in low glucose DMEM (Sigma-Aldrich) supplemented as described above. Human primary fibroblasts were grown in DMEM (Gibco) supplemented with 10 % FBS, 1 % glutamine and 1 % penicillin-streptomycin. The PDX-derived cell cultures LU-NB-2 and LU-NB-3 were cultured as previously described (Persson et al., 2017). Briefly, the cells were maintained in a 3:1 mixture of DMEM:F12 supplemented with 40 ng/ml FGF, 20 ng/ml EGF, 1% penicillin-streptomycin and 2% B27 without vitamin A. For differentiation experiments, plates were coated with Biolaminin 521 LN (Biolamina).

The sex and other information of the patients from which the different cell lines were generated is provided in Supplemental Table 1.

Small molecules, inhibitors and treatments

For *in vitro* experiments, TOFA, Cerulenin, Orlistat, ATRA, CI-976, 10058-F4, VIII trifluoroacetate, doxycycline, and 2-(hydroxypropyl)-β-cyclodextrin were from Sigma-Aldrich. SCH772984 was from Santa Cruz Biotechnologies. Delipidized fetal bovine serum was from Pan Biotech. Soraphen A was a kind gift from Professor Rolf Müller (Helmholtz Institute for Pharmaceutical Research Saarland (HIPS), Saarland, Germany). For animal experiments, TOFA was from Cayman Chemicals, and Orlistat from STADA laboratories.

Here UB006 refers to (–)-UB006 enantiomer, (4S,5S)-4-(Hydroxymethyl)-3-methylene-5-octyldihydrofuran-2(3H)-one, and was synthesized as described (Makowski *et al*., 2017).

The concentrations used for each compound were as follows: TOFA (2.5 – 40 µM), Cerulenin (5 – 18 µM), Orlistat (20 – 50 µM), Soraphen A (5 – 10 µM), UB006 (500 nM – 15 µM), ATRA

(0.5 – 10 µM), CI-976 (30 µM), 10058-F4 (30 µM), doxycycline (2 µg/ml), VIII trifluoroacetate (0.5-10 µM), SCH772984 (1 µM).

FASN knock-down with siRNA

SK-N-BE(2) were plated in 6 well plates at a density of 50 000 cells/well and incubated overnight. The next day, the cells were transfected with 30 nM siRNA targeting human *FASN* (s5032, ThemoFisher Scientific) or with a negative control siRNA (4390846, ThemoFisher Scientific), using Lipofectamine RNAiMAX (ThemoFisher Scientific) following the manufacturer's instructions. After 72 hours, cells were either fixed for immunofluorescence or harvested for protein extraction.

METHOD DETAILS

Determination of cell growth and viability

For cell growth curves, cells where seeded in 24 well plates and after the indicated treatments and incubation times, cells were trypsinized, mixed with Trypan Blue (Sigma-Aldrich), and counted in a hemocytometer chamber under an inverted microscope.

For WST-1 viability assay, cells were seeded in 96 well plates and treated as indicated. 10 µl of WST-1 (Roche) were added per well and after 1 h incubation, absorbance at 480 nm was measured in a LUMIstar Omega plate reader (BMG Labtech). At least three independent experiments were performed.

Histone extraction for Western Blot

Cells were harvested and washed twice in cold PBS. Both PBS and subsequent buffers were supplemented with 5 mM sodium butyrate to retain histone acetylation. Cells were re-suspended and lysed for 10 min on ice with gentle stirring, using Triton Extraction Buffer (TEB) (PBS containing 0.5% Triton X 100 (v/v), 2 mM phenylmethylsulfonyl fluoride (PMSF), and 0.02% (w/v) Na_3N) at around 100 000 cells in 100 µl. Lysates were centrifuged at 6 500 x g, 10 min at 4°C to spin down the nuclei. Supernatants were discarded and pellets (nuclei) washed in half the volume of TEB and centrifuged as before. Pellet was re-suspended in 0.2 N HCl. Histones were extracted overnight at 4°C with gentle stirring. Samples were centrifuged 6 500

x g, 10 min at 4°C to pellet debris. The supernatant was collected for protein quantification and Western Blot.

Western Blot

Whole-cell extracts were prepared using RIPA buffer (Sigma-Aldrich) supplemented with Halt Protease and Phosphatase Inhibitor Mixture (Thermo Fisher Scientific); 10-20 µg of protein (2 µg in the case of extracted histones) were resolved in Bolt™ 4-12 % Bis-Tris Plus Gels (Thermo Fisher Scientific) and transferred to nitrocellulose membranes using BioRad Trans-blot Turbo. For probing FASN, 3-8% Tris-acetate gels (Thermo Fisher Scientific) and wet transfer for 3 hours, 70 V were used. For analyzing histones, 18% polyacrylamide gels were used. After blocking with non-fat milk, the membranes were incubated with the corresponding antibodies. The monoclonal antibodies for c-Myc (9E10), MYCN (B8.4.B), β-Actin (C4), and α-Tubulin (DM1A) were from Santa Cruz Biotechnology. The monoclonal antibodies for AMPKα, phospho-AMPKα (40H9), ERK, phospho-ERK, AKT, phospho-AKT (D9E), GSK3β (27C10), phospho-GSK3β, CREB (D76D11), phospho-CREB (1B6), acetyl-histone H3 Lys 9 (C5B11) and acetyl-histone H3 Lys 27 (D5E4) were from Cell Signaling Technology. FASN polyclonal antibody was from Novus. Rabbit polyclonal antibody against histone 3 (H3; ab1791) was from abcam. Incubation with primary antibodies was followed by anti-mouse or anti-rabbit HRP-conjugated secondary antibodies (DAKO). Bands were detected using SuperSignal™ West Pico PLUS Chemiluminescent Substrate (Thermo Fisher Scientific) and imaged in a ChemiDoc XRS+ (Bio Rad). The pictures showed here are representative of at least three independent experiments.

Immunocytochemistry

Cells seeded in glass coverslips were fixed in 4 % buffered PFA (Histolab) and blocked for 1 h at room temperature with blocking solution (5 % goat serum, 0.25 % Triton X-100, 1 % BSA, 0.05 % sodium azide, all from Sigma-Aldrich, in PBS). Primary antibodies (Tubulin B3 from Abcam; TrkA from Millipore, vimentin (V9) from DAKO; SCG2 from Atlas antibodies) diluted in blocking solution were incubated overnight at +4°C. After washing, the slides were incubated for 1 h at room temperature with the appropriate fluorophore-conjugated antibodies (AlexaFluor 568 and 488, Thermo Fisher Scientific). DAPI (1:10,000; Life Technologies) was used for nuclear stain. Pictures were taken on a Zeiss Axiovert 200M microscope with the

Axiovision software, or in an Olympus IX73 microscope with the CellSens Dimensions software. The pictures showed here are representative of three independent experiments.

Immunohistochemistry analysis of tumor sections

For immunohistochemistry (IHC) on IMR-32, SK-N-BE(2), and SK-N-AS xenograft tumors, transverse sections of 5 µm thickness from paraffin-embedded tissues were stained with the EnVision System (Dako) according to the manufacturer's instructions. Briefly, paraffin was removed and samples were re-hydrated by consecutive xylol-alcohol (all from Sigma-Aldrich) gradients. Sample blocking was followed by overnight incubation at +4°C with the following primary antibodies diluted in blocking solution: rabbit anti-Ki67 (1:250, Abcam ab16667), mouse anti-MYCN (1:200, Santa Cruz sc-56729), rabbit anti c-MYC (1:250, Santa Cruz sc-789), mouse anti-tubulin B3 (1:250, Abcam ab7751) or rabbit anti-SCG2 (1:250, Sigma-Aldrich HPA011893). Samples were incubated with HRP-polymer for 20 min, followed by 30 seconds-3 min incubation with DAB working solution. Nuclear staining with Meyer's hematoxylin (Dako) was performed for 2 min. Samples were dehydrated by consecutive alcohol-xylol gradients and mounted with Roti-histokitt mounting medium (Roth). The pictures showed here are representative of three independent experiments.

Phospho-antibody array

To analyze the phosphorylation and activation status of different cancer-related signaling pathways, the Proteome Profiler Human Phospho-Kinase Array Kit (R&D systems) was used, according to the manufacturer's instructions. Briefly, SK-N-BE(2) cells were treated for 24 h with DMSO, TOFA, Cerulenin, or Orlistat, and then washed with PBS and lysed with the kit lysis buffer, followed by protein quantification. 300 µg of protein per cell lysates were incubated with the array membranes overnight at +4°C, followed by incubation with detection antibody cocktail for 2 h at room temperature, 30 min with Streptavidin-HRP and finally Chemi Reagent Mix. Pictures were taken on a ChemiDoc XRS+ imaging system (Bio-Rad).

Extracellular flux assay

XF96 Extracellular Flux Analyzer (Agilent Seahorse Technologies) was used to determine the mitochondrial and glycolytic functions. Using Seahorse 96 well plates, SK-N-BE(2) were plated

at 8,000 cells per well, Kelly, IMR-32, SH-EP, and SK-N-SH cells at 10,000 cells per well, Tet-21/N cells at 6,000 cells per well, in 96-well plates (Agilent Seahorse Technologies) in regular cell culture medium and treated with DMSO or fatty acid synthesis inhibitors for 72 h. The oxygen consumption rate (OCR) and extracellular acidification rate (ECAR) were measured after following treatment scheme. OCR and ECAR were measured 1 h after the cells had been conditioned in the assay medium, pH 7.4, at +37°C in a CO_2-free incubator. Seahorse OCR and ECAR assay kits were used following the manufacturer's instructions. After the experiments were finished, normalization was performed using total protein amount per well. Protein contents were measured by DC protein assay (Bio-Rad). OCR and ECAR values were reported in pmol/min/µg protein and mpH/min/µg protein, respectively. At least three independent experiments were performed.

qPCR

Total RNA was isolated using TRIzol reagent (Invitrogen) and cleaned up with RNeasy kit (Qiagen). cDNA was synthesized using iScript cDNA synthesis kit (Bio-Rad) according to manufacturer's instructions. Real-time qPCR analyses of mRNA expression for MYCN using iTaq universal SYBR Green supermix (BioRad) was carried out on a StepOnePlus Real-Time PCR system (Applied Biosystems) according to manufacturer's instruction. Samples were run in triplicate and normalized to the mRNA levels of β-*2-microglobulin* (*B2M*) which served as internal control. Relative expression was calculated using the ΔΔCT method. Primer sequences are listed below. At least three independent experiments were performed.

B2M (forward): TGCTGTCTCCATGTTTGATGTATC
B2M (reverse): TCTCTGCTCCCCACCTCTAAGT
NPY (forward): TCCAGCCCAGAGACACTGATT
NPY (reverse): AGGGTCTTCAAGCCGAGTTCT
SCG2 (forward): GAAGCCGAATGGATCAGTGGA
SCG2 (reverse): TGGTCTAAGTCAGCCTCTGAG
TH (forward): TCGAGCGCACGAAGTACT
TH (reverse): TGTGAAGGTGTTGAGACGTTTG
FASN (forward): AAGGACCTGTCTAGGTTTG
FASN (reverse): TGGCTTCATAGGTGACTTC

Electron microscopy

Cells were fixed (2.5% (w/v) glutaraldehyde in 0.1 M phosphate buffer, pH 7.4) at room temperature for 30 min, before being scraped off and transferred to microcentrifuge tubes for further fixation and storage at 4°C. Cells were next rinsed in 0.1 M phosphate buffer and centrifuged. Pellets were post fixed in 2 % (w/v) osmium tetroxide in 0.1 M phosphate buffer (pH 7.4) at +4°C for 2 h, dehydrated in ethanol followed by acetone, and embedded in LX-112 (Ladd). Ultrathin sections (~40–50 nm) were cut using a Leica EM UC 6 ultramicrotome (Leica). Sections were contrasted with uranyl acetate followed by lead citrate and examined in a Tecnai 12 Spirit Bio TWIN transmission electron micro- scope (FEI) at 100 kV. Digital images were taken using a Veleta camera (Olympus Soft Imaging Solutions). The pictures shown are representative from three independent experiments.

Cell cycle analysis

Cells were treated for the indicated time points in six well plates, washed with ice-cold PBS, trypsinized, collected in cell culture medium and washed three times by centrifugation. For fixation, cell pellets were immediately resuspended in ice-cold 70 % ethanol and kept at -20°C until the day of the assay. Then, the cells were washed with PBS and incubated at room temperature for 30 min in PBS containing 100 µg/ml RNase A (Sigma-Aldrich) and 40 µg/ml propidium iodide (PI) (Sigma-Aldrich). PI signal in the single cell suspension was recorded using a FACScan instrument. At least three independent experiments were performed.

Analysis of Free Fatty Acids by Ultra Performance Supercritical Fluid Chromatography-Tandem Mass Spectrometry

Free fatty acids and internal standard mixture (heptadecanoic acid, deuterated palmitoleic acid and deuterated docosahexaenoic acid) were extracted with 0.5 ml of the organic solvent mixture, isopropanol: n-heptane: 1 M hydrochloric acid (40:10:1 v/v/v) for 15 minutes. Samples were kept at room temperature for 10 min followed by the addition of 0.5 ml of water. Plasma lipids were extracted into 1 ml of n-heptane. During the extraction, the lipids were protected against oxidation by the addition of 0.05 mg/ml butylated hydroxytoluene (BHT) to the organic solvent mixture. The n-heptane layer was dried under N_2, and was reconstituted in 200 µl of MeOH prior to the analysis.

The analysis of FFA was performed on a supercritical fluid chromatography (Waters ACQUITY® UPC2™) coupled with tandem mass spectrometry (XEVO® TQS). In brief, the separation of FFA was carried out with a Viridis HSS C18 SB 1 column (3.0 mm x100 mm, 1.8 µm) (Waters) using a mobile phase of CO_2 and methanol containing 10 mM ammonium acetate as the modifier with a gradient elution. The total run time was 3 min. Methanol with 0.5 % water was used as the make-up solvent at a flow rate of 0.2 ml/min. The mass spectrometer was operated with a unit mass resolution in ESI negative mode and generated abundant $[M-H]^-$ ions at optimal collision energy and cone voltage for specific fatty acid parent/parent ion pairs. The capillary voltage was 2.5 kV, source temperature was +150°C, and desolvation temperature was +500°C with a dwell time of 0.003 sec. The cone and desolvation gas flow rate were 150 and 800 L/h, respectively. All data were acquired and processed using Mass Lynx™ 4.1 software (Waters) and the data acquisition range was *m/z* 50-600. The linearity for the fatty acids over the range of 5-5,000 ng/ml with correlation coefficients (r^2) of 0.9989-0.9998 with sougate buffer. The limits of quantification were 0.001 - 0.003 µg/sample. The quantification was accomplished using corresponding internal standards for saturated, monounsaturated, and polyunsaturated fatty acids.

Analysis of Free Cholesterol by Ultra Performance Supercritical Fluid Chromatography-Tandem Mass Spectrometry

Lipid extraction was performed with 1ml heptane. The extracted heptane fractions were evaporated under gentle stream of nitrogen. The dried samples were re-dissolved in 100 µl of the derivatization reagent, consisting of 2-methyl-6-nitrobenzoic acid anhydride (100 mg), 4-dimethylaminopyridine (30 mg), picolinic acid (80 mg), pyridine (1.5 ml), and triethylamine (0.2 ml). Samples were incubated at +80°C for 60 min and picolinyl ester of cholesterol was extracted with 1 ml of heptane by vortexing the mixture for 5 min, followed by centrifuging at 4,000 rpm for 5 min.

Cholesterol picolinyl ester was analyzed with Supercritical Fluid Chromatography (Waters ACQUITY® UPC^2™, Milford, MA) coupled with tandem mass spectrometry (XEVO® TQ-S). In brief, the separation of cholesterol derivative was carried out with a Viridis BEH column (100 mm, 3.0 mm, 1.7 µm at +40°C (Waters)) and 0.1 % formic acid in methanol as the co-solvent, at a flow rate of 1.2 ml/min. The total time for the analysis was 4 min. The desolvation gas was

nitrogen, and the collision gas was argon (0.25 ml/min). The data acquisition range was *m/z* 50-700. Cholesterol picolinyl ester was ionized in the ESI probe with cone voltage of 30 V to form sodium adduct of molecular ion and deuterated internal standards were 514 and 522. The molecular ions were subsequently fragmented into a [picolinic acid + Na]$^+$ ion of *m/z* = 146 and 153, respectively. Multiple reaction monitoring (MRM) of the transition 514/146 was used for the quantification as the most intense transition. Data were acquired and analyzed with Waters MassLynx v4.1 software. Finally, the quantification was accomplished using corresponding deuterated internal standard of cholesterol d8.

Duplicate analyses of each sample were performed and the average values were reported (CV <3 %). The linearity of the method was evaluated over a range of concentrations (0.1 – 10,000 ng/ml) and the correlation coefficients (R^2) were 0.998. The limit of quantification was 0.01 ng/ml.

Measurements of ceramides and sphingomyelins species by liquid chromatography tandem mass spectrometry

Cell pellets were fortified with internal standards for endogenous ceramides (C12 ceramide) and sphingomyelins (C12 sphingomyelin) prior to extraction. Lipid extraction was carried out with a mixture of ice-cold chloroform–methanol (2:1). Subsequently, samples were sonicated and then incubated for 12 hours at +48°C. Samples were cooled down to room temperature, and chloroform: water (1:2, v/v) mixture was added. Samples were mixed to avoid formation of an emulsion. Samples were then centrifuged at 2,000 g for 10 min and the upper layer was carefully removed. The remaining bottom layer was dried under nitrogen stream. Samples were re-suspended in ethanol. Stock solutions of lipid standards/internal standards (Avanti Polar Lipids) were prepared to achieve a final concentration of 1 µg/mL. UPLC column (Kinetex C18, 2.1 × 100 mm id. 2.6 µm) was purchased from Phenomenex (Torrance). The column was equipped with a Security Guard Ultra Cartridge. The UPLC was Acquity Waters (Waters) and was run at 200 µl/min. The extracted samples were injected into the pre-column followed by the analytical column and delivered to the mass spectrometer. A gradient elution was used for separating the lipid analytes. The buffer (A) was 10 mM ammonium acetate, 0.1% formic acid in water and the other buffer (B) was 10 mM ammonium acetate, 0.1% formic acid in acetonitrile/isopropanol (4:3, v/v). The separation was achieved at room temperature (approximately +20°C). The sample vials were kept at +8 °C in a cooled auto sampler.

Separation was started at 65 % B (2 min), then up to 75 % in 0.5 min and followed to 100 % in 15 min and held for 5 min. Equilibration was then down to 65 % B in 0.1 min and held there for 10 min before the injection of the next sample. The lipid extracts were analyzed by ABI 3200 Q trap mass spectrometer (Applied Biosystems). At the end of each injection, a start signal was sent to the MS to start data acquisition. The MS was run multiple reactions monitoring (MRM) mode with the transitions obtained from (Bielawski et al., 2006). Duplicate analyses of each sample were performed and the average values were reported for both ceramides and sphingomyelin (CV < 11 %). The linearity of the method was evaluated over a range of concentrations for ceramides and sphingomyelin of 0.1 - 100 µM and 0.1-30 µM, respectively. The correlation coefficients (r2) were 0.998 for both analytes. The limit of quantification was 0.01 µM for both ceramides and sphingomyelin.

Metabolic tracing

SK-N-BE(2) cells were cultured as described above and treated with DMSO or 40 µM 10058-F4 for 72 h, at which point the medium was changed to labelling medium (glucose-, glutamine-, and phenol-red free DMEM, Sigma-Aldrich) containing 25 mM [$U^{13}C$]-glucose or 2 mM [$U^{13}C$]-glutamine (both from Cambridge Isotopes Laboratories). The cells were then cultured for further 48 h. For mass spectrometry (MS) analysis, cells were harvested in 250 µL Methanol/H_2O (80/20, v/v) with added standards (40 µM tridecanoic acid and ibuprofen) 30 µL 0.2 M HCl, 2x 100 µL $CHCl_3$, 2x 100 µL water was then added with vortexing in between. The suspension was then centrifuged at 16,000 g for 5 min, the lower lipid phase was then washed with synthetic polar phase (CH_3Cl/Methanol/H_2O, 58/33/8, v/v/v) and evaporated to dryness under N_2 at +45°C. For saponification lipids were resuspended in Methanol/H_2O (80/20, v/v) containing 0.3 M KOH, heated at +80°C for 1 h and washed twice with 0.5 mL hexane. After addition of 50 µL formic acid, fatty acids were subsequently extracted twice with 0.5 mL hexane and evaporated to dryness under N_2 at +45°C. For LC/MS analysis the lipid phase residue was dissolved in 100 µl isopropyl alcohol. For LC/MS analysis of fatty acids 5 µl of each sample was applied to a C8 column (Acclaim RSLC C8 column, 2.2 µm particles, 50 × 2.1 mm, Thermo Fisher Scientific) (at +40°C), with mobile phase buffer A consisting of 0.1% formic acid in CH_3CN/H_2O (10/90, v/v), and solvent B consisting of 0.1% formic acid in CH_3CN/H_2O (90/10, v/v). The flow rate was maintained at 350 µL/min and eluent was directed to the ESI source of the QE-MS from 3 min to 27 min after sample injection. MS parameters

for LC/MS: Sheath gas, 30; spray voltage, 2.6 kV. Capillary temperature +320°C, aux gas heater temperature: +120°C and S-lens voltage was 55. A full scan range from 150 to 460 (*m/z*) in negative ion mode was used. The resolution was set at 70,000. The maximum injection time was 100 ms with an AGC-target of 1E6 ions. Peaks corresponding to the calculated ion masses (MIM-H^+ ± 2 mMU) were integrated using TraceFinder software (Thermo Fisher Scientific). The equipment used for LC/MS analysis was a Thermo Fisher Scientific Dionex Ultimate 3000 UHPLC system hyphenated with a Q Exactive mass spectrometer (QE-MS) equipped with a HESI probe (Thermo Fisher Scientific). UPLC-precolumn: Acclaim C8 column (5 µm particles, 10 × 2 mm) (Thermo Fisher Scientific). UPLC-column: Acclaim RSLC C8 column (2.2 µm particles, 50 × 2.1 mm) (Thermo Fisher Scientific).

QUANTIFICATION AND STATISTICAL ANALYSIS

Statistical analysis

All *in vitro* and *in vivo* experiments were analyzed by Student's t-test comparing vehicle-treated to inhibitor-treated conditions. All data is presented as mean ± standard deviation (SD) with *, **, ***, **** indicating p<0.05, p<0.01, p<0.001, and p<0.0001, respectively.

Analysis of patient data

For survival and correlation analyses, three neuroblastoma patient cohorts with published RNA sequencing expression data from 649 (Kocak *et al*., 2013), 88 (Molenaar *et al*., 2012), and 283 (Rajbhandari *et al*., 2018; referred to as NRC) patients were used. Clinical and expression data of the genes of interest were extracted from the R2 platform (https://hgserver1.amc.nl/) and analyzed using Excel and GraphPad Prism software. High and Low *FASN* and *ACACA* were defined by the median, while *SCG2* and *TRKA* levels were presented as quartiles of gene expression levels, where Low = Q1, Medium = Q2, Medium-High = Q3, and High = Q4. When only two conditions were compared, the data was analyzed by Student's t-test. Significance of overall survival (OS) curves was analyzed by Mantel-Cox test. When more than three conditions were compared, the data was analyzed by one-way ANOVA, with *, **, ***, **** indicating p<0.05, p<0.01, p<0.001, and p<0.0001, respectively.

KEY RESOURCES TABLE

REAGENT or RESOURCE	SOURCE	IDENTIFIER
Antibodies		
c-MYC (9E10)	Santa Cruz Biotechnology	Cat# sc-40<br>RRID: AB_627268
c-MYC (A-14)	Santa Cruz Biotechnology	Cat# sc-789<br>RRID: AB_631274
MYCN (B8.4.B)	Santa Cruz Biotechnology	Cat# sc-53993<br>RRID: AB_831602
MYCN (NCM II 100)	Santa Cruz Biotechnology	Cat# sc-56729<br>RRID: AB_2266882
β-Actin (C4)	Santa Cruz Biotechnology	Cat# sc-47778<br>RRID: AB_2714189
α-Tubulin (DM1A)	Santa Cruz Biotechnology	Cat# sc-32293
AMPKα	Cell Signaling Technology	Cat #2532<br>RRID: AB_330331
p-AMPKα (Thr172)	Cell Signaling Technology	Cat #2535<br>RRID: AB_331250
ERK1/2	Cell Signaling Technology	Cat #4695<br>RRID: AB_390779
p-ERK1/2	Cell Signaling Technology	Cat #9101<br>RRID: AB_331646
AKT (pan) (C67E7)	Cell Signaling Technology	Cat #4691<br>RRID: AB_915783
p-AKT (Ser473)	Cell Signaling Technology	Cat #4060<br>RRID: AB_2315049
GSK3β (27C10)	Cell Signaling Technology	Cat #9315<br>RRID: AB_490890
p-GSK3β (Ser9)	Cell Signaling Technology	Cat #9336<br>RRID: AB_331405
CREB (D76D11)	Cell Signaling Technology	Cat #4820<br>RRID: AB_1903940
p-CREB (Ser133)	Cell Signaling Technology	Cat #9196<br>RRID: AB_331275
Acetyl-Histone H3 Lys9 (C5B11)	Cell Signaling Technology	Cat# 9649<br>RRID: AB_823528
Acetyl-Histone H3 Lys27 (D5E4)	Cell Signaling Technology	Cat# 8173<br>RRID: AB_10949503
Tubulin B3	Abcam	Cat# ab7751<br>RRID: AB_306045
Ki67	Abcam	Cat# ab16667<br>RRID: AB_302459
Histone H3	Abcam	Cat# ab1791<br>RRID: AB_302613
TRKA	Millipore	Cat# 06-574<br>RRID: AB_310180
Vimentin (V9)	Dako	Cat# GA630<br>RRID: AB_2827759
SCG2	Sigma-Atlas Antibodies	Cat# HPA011893<br>RRID: AB_1856656
FASN	Novus	Cat# NB400-114<br>RRID: AB_10002181

Proteome Profiler Human Phospho-Kinase Array Kit	R&D Systems	Cat# ARY003C
Chemicals, Peptides, and Recombinant Proteins		
Seahorse XF glycolysis stress test kit	Agilent	Cat# 103020-100
Seahorse XF Mitostress kit	Agilent	Cat# 103015-100
Seahorse XF Base medium	Agilent	Cat# 103334-100
Seahorse FluxPak	Agilent	Cat# 102416-100
[$U^{13}C$]-glucose	Cambridge Isotopes Laboratories	Cat# CLM-1822-H
[$U^{13}C$]-glutamine	Cambridge Isotopes Laboratories	Cat# CLM-1822-H
ATRA	Sigma-Aldrich	Cat# R2625
TOFA	Sigma-Aldrich	Cat# T6575<br>CAS 54857-86-2
Cerulenin	Sigma-Aldrich	Cat# C2389
Orlistat	Sigma-Aldrich	Cat# O4139
10058-F4	Sigma-Aldrich	Cat# F3680
VIII trifluoroacetate	Sigma-Aldrich	Cat# A6730
CI-976	Sigma-Aldrich	Cat# C3743<br>CAS 114289-47-3
SCH772984	Santa Cruz Biotechnologies	Cat# SCH772984<br>CAS 942183-80-4
Soraphen A	Professor Rolf Müller, Helmholtz Institute, Saarland, Germany	CAS 122547-72-2
UB006	Professor Dolors Serra, University of Barcelona, Spain	Makowski et al. 2017.
Lipofectamine RNAiMAX	ThemoFisher Scientific	Cat# 13778075
Critical Commercial Assays		
RNA extraction RNeasy kit	Qiagen	Cat# 74104
iScript cDNA synthesis kit	BioRad	Cat# 1708891
iTaq Universal SYBR Green Supermix	BioRad	Cat# 172-5124
Proteome Profiler Human Phospho-Kinase Array Kit	R&D systems	Cat# ARY003C
EnVision G/2 Doublestain System Rabbit/Mouse	Dako	Cat# K536111-2
Experimental Models: Cell Lines		
SK-N-BE(2)	ATCC	Cat# CRL-2271
Human primary fibroblasts	ATCC	Cat# PCS-201-012
IMR-32	ATCC	Cat# CCL-127
SK-N-AS	ATCC	Cat# CRL-2137
SK-N-SH	ATCC	Cat# HTB-11
SH-SY5Y	ATCC	Cat# CRL-2266
KELLY	ECACC	Cat# 92110411
SK-N-BE(2)sh*MYCN*	Professor Christer Einvik, Arctic University of Norway, Tromsö, Norway	Henriksen et al. 2011

SH-EP	Professor Sven Påhlman, Lund University, Sweden	
KCN-69n	Professor Sven Påhlman, Lund University, Sweden	
TET-21/N	Professor Manfred Shwab, DKFZ, Heidelberg, Germany	Lutz et al. 1996
TH-*MYCN* tumor spheres	This paper	Ribeiro et al. 2016
LU-NB-2	Professor Daniel Bexell, Lund University, Sweden	Persson et al. 2017
LU-NB-3	Professor Daniel Bexell, Lund University, Sweden	Persson et al. 2017
Experimental Models: Organisms/Strains		
Female NMRI-Foxn1^{nu} mice	Taconic	Cat# NMRINU-F
Oligonucleotides		
B2M	Sigma-Aldrich	TGCTGTCTCCATGTTTGATGTATC / TCTCTGCTCCCCACCTCTAAGT
NPY	Sigma-Aldrich	TCCAGCCCAGAGACACTGATT / AGGGTCTTCAAGCCGAGTTCT
SCG2	Sigma-Aldrich	GAAGCCGAATGGATCAGTGGA / TGGTCTAAGTCAGCCTCTGAG
TH	Sigma-Aldrich	TCGAGCGCACGAAGTACT / TGTGAAGGTGTTGAGACGTTTG
siRNA against human *FANS*	ThemoFisher Scientific	Cat# s5032
Negative control siRNA	ThemoFisher Scientific	Cat# 4390846
Software and Algorithms		
Seahorse Wave Controller Software	Instrument software by Agilent	https://www.agilent.com/en/products/cell-analysis/cell-analysis-software/instrument-software/wave-controller-2-4
ImageLab	Instrument Software by Bio-Rad	https://www.bio-rad.com/en-se/product/image-lab-software?ID=KRE6P5E8Z

GraphPad Prism 6	GraphPad	https://www.graphpad.com/scientific-software/prism/
R2: Genomics Analysis and Visualization Platform	Dr. Jan Koster, Amsterdam University, The Netherlands	https://hgserver1.amc.nl/cgi-bin/r2/main.cgi
MassLynx TM 4.1 Mass Spectrometry Software	Instrument Software by Waters	https://www.waters.com/waters/en_SE/MassLynx-Mass-Spectrometry-Software
Adobe Illustrator CS6	Adobe	www.adobe.com
Adobe Photoshop CS6	Adobe	www.adobe.com

SUPPLEMENTAL REFERENCES

ATCC. *ATCC Cell Lines* [Online]. Available: https://www.lgcstandards-atcc.org/en/Products/Cells_and_Microorganisms/Cell_Lines.aspx.

Bielawski, J., Szulc, Z. M., Hannun, Y. A. & Bielawska, A. 2006. Simultaneous quantitative analysis of bioactive sphingolipids by high-performance liquid chromatography-tandem mass spectrometry. *Methods,* 39**,** 82-91.

Braekeveldt, N., Wigerup, C., Gisselsson, D., Mohlin, S., Merselius, M., Beckman, S., Jonson, T., Borjesson, A., Backman, T., Tadeo, I., Berbegall, A. P., Ora, I., Navarro, S., Noguera, R., Pahlman, S. & Bexell, D. 2015. Neuroblastoma patient-derived orthotopic xenografts retain metastatic patterns and geno- and phenotypes of patient tumours. *Int J Cancer,* 136**,** E252-E261.

Henriksen, J. R., Haug, B. H., Buechner, J., Tomte, E., Lokke, C., Flaegstad, T. & Einvik, C. 2011. Conditional expression of retrovirally delivered anti-MYCN shRNA as an in vitro model system to study neuronal differentiation in MYCN-amplified neuroblastoma. *BMC Dev Biol,* 11**,** 1.

Lutz, W., Stohr, M., Schurmann, J., Wenzel, A., Lohr, A. & Schwab, M. 1996. Conditional expression of N-myc in human neuroblastoma cells increases expression of alpha-prothymosin and ornithine decarboxylase and accelerates progression into S-phase early after mitogenic stimulation of quiescent cells. *Oncogene,* 13**,** 803-12.

Persson, C. U., Von Stedingk, K., Bexell, D., Merselius, M., Braekeveldt, N., Gisselsson, D., Arsenian-Henriksson, M., Pahlman, S. & Wigerup, C. 2017. Neuroblastoma patient-derived xenograft cells cultured in stem-cell promoting medium retain tumorigenic and metastatic capacities but differentiate in serum. *Sci Rep,* 7.

Ribeiro, D., Klarqvist, M. D., Westermark, U. K., Oliynyk, G., Dzieran, J., Kock, A., Savatier Banares, C., Hertwig, F., Johnsen, J. I., Fischer, M., Kogner, P., Loven, J. & Arsenian Henriksson, M. 2016. Regulation of Nuclear Hormone Receptors by MYCN-Driven miRNAs Impacts Neural Differentiation and Survival in Neuroblastoma Patients. *Cell Rep,* 16**,** 979-93.

Thiele, C. 1998. Neuroblastoma Cell Lines. *In:* MASTERS, J. (ed.) *Human Cell Culture.* Lancaster, UK.: Kluwer Academic Publishers.

Weiss, W. A., Aldape, K., Mohapatra, G., Feuerstein, B. G. & Bishop, J. M. 1997. Targeted expression of MYCN causes neuroblastoma in transgenic mice. *EMBO J,* 16**,** 2985-95.
